# Extensive Differences in Gene Expression Between Symbiotic and Aposymbiotic Cnidarians

**DOI:** 10.1534/g3.113.009084

**Published:** 2013-12-24

**Authors:** Erik M. Lehnert, Morgan E. Mouchka, Matthew S. Burriesci, Natalya D. Gallo, Jodi A. Schwarz, John R. Pringle

**Affiliations:** * Department of Genetics, Stanford University School of Medicine, Stanford, California 94305; † Department of Ecology and Evolutionary Biology, Cornell University, Ithaca, New York 14850; ‡ Biology Department, Vassar College, Poughkeepsie, New York 12604

**Keywords:** anemone, dinoflagellate, innate immunity, metabolic compartmentation, symbiosis

## Abstract

Coral reefs provide habitats for a disproportionate number of marine species relative to the small area of the oceans that they occupy. The mutualism between the cnidarian animal hosts and their intracellular dinoflagellate symbionts provides the nutritional foundation for coral growth and formation of reef structures, because algal photosynthesis can provide >90% of the total energy of the host. Disruption of this symbiosis (“coral bleaching”) is occurring on a large scale due primarily to anthropogenic factors and poses a major threat to the future of coral reefs. Despite the importance of this symbiosis, the cellular mechanisms involved in its establishment, maintenance, and breakdown remain largely unknown. We report our continued development of genomic tools to study these mechanisms in *Aiptasia*, a small sea anemone with great promise as a model system for studies of cnidarian–dinoflagellate symbiosis. Specifically, we have generated *de novo* assemblies of the transcriptomes of both a clonal line of symbiotic anemones and their endogenous dinoflagellate symbionts. We then compared transcript abundances in animals with and without dinoflagellates. This analysis identified >900 differentially expressed genes and allowed us to generate testable hypotheses about the cellular functions affected by symbiosis establishment. The differentially regulated transcripts include >60 encoding proteins that may play roles in transporting various nutrients between the symbiotic partners; many more encoding proteins functioning in several metabolic pathways, providing clues regarding how the transported nutrients may be used by the partners; and several encoding proteins that may be involved in host recognition and tolerance of the dinoflagellate.

Coral reefs comprise only a small part of the world’s ocean environment but are habitats for a disproportionately large fraction of all marine species. Corals are able to produce the massive and biologically rich reef habitats despite growing in nutrient-poor waters because of the energy acquired through their mutualistic symbiosis with dinoflagellates in the genus *Symbiodinium*. These unicellular algae inhabit the symbiosomes (phagosome-derived vacuoles) of gastrodermal cells in corals and other cnidarians (Figure 1) and transfer up to 95% of their photosynthetically fixed carbon to the host (Muscatine *et al.* 1984). Reef-building corals are declining worldwide due largely to anthropogenic causes, which include pollution, destructive fishing practices, and increasing sea-surface temperatures (De’ath *et al.* 2012). Such stresses can lead to coral “bleaching”, in which the algae lose their photosynthetic capacity and/or are lost altogether by the host. In severe cases, bleaching can result in the death of the host. The threat of bleaching is particularly alarming because many corals already live near the upper limits of their thermal tolerances, and most climate-change models predict that these tolerances will be exceeded frequently in the coming decades, leading to widespread coral death and a resulting loss of the reef habitats (Hughes *et al.* 2003).

**Figure 1:**
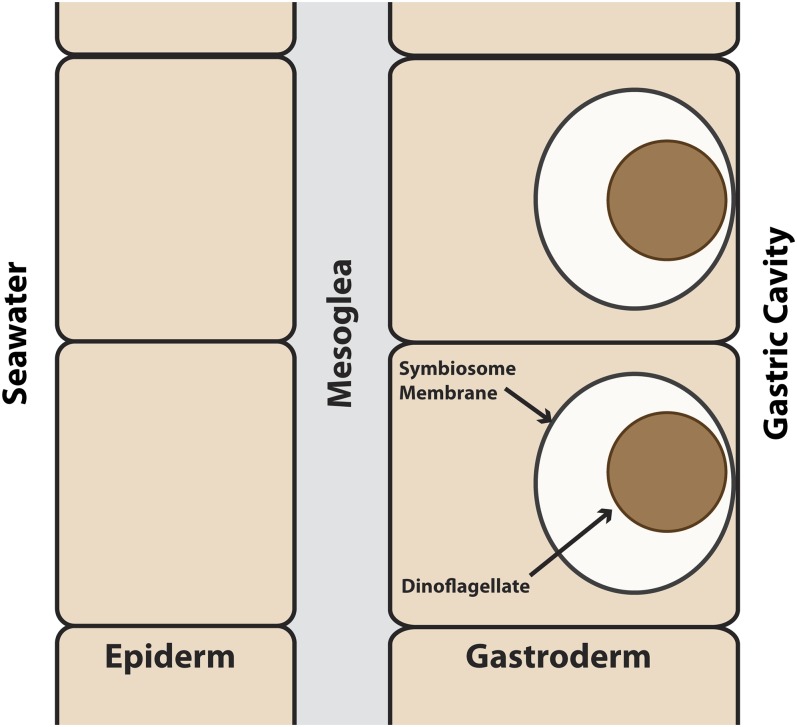
The spatial organization of cnidarian–dinoflagellate symbiosis. A simplified schematic diagram of a section of cnidarian body wall is shown; note that the drawing is not to scale (*e.g.*, the dinoflagellate typically fills most of the symbiosome). The two major tissue layers are the epiderm, which faces the outside sea water and lacks both dinoflagellate symbionts and direct access to food in the gastric cavity, and the gastroderm, which faces the gastric cavity and may contain dinoflagellate symbionts in some of its cells. These two cell layers are separated by the largely acellular mesoglea. After phagocytosis by a host gastrodermal cell, the dinoflagellate resides within a “symbiosome” (believed to be derived from a phagosome that does not fuse with lysosomes) and transfers fixed carbon to the host.

Despite the great ecological importance of cnidarian–dinoflagellate symbioses, little is known about the cellular and molecular mechanisms by which these relationships are established, maintained, or disrupted (Davy *et al.* 2012). This situation has resulted in part from the difficulties inherent in studying corals directly (Weis *et al.* 2008). Thus, we and others have turned to the small sea anemone *Aiptasia*, which is normally symbiotic with dinoflagellates closely related to those found in corals (Lajeunesse *et al.* 2012) but offers many experimental advantages (Weis *et al.* 2008). In particular, *Aiptasia* lacks the calcareous skeleton that hinders biochemical and microscopic analyses of corals, grows rapidly by asexual reproduction under standard aquarium conditions to form large clonal populations, can be induced to spawn and produce larvae throughout the year in the laboratory (S. F. Perez and J. R. Pringle, unpublished data), and (importantly for this study) can be maintained indefinitely in an aposymbiotic (bleached) state so long as it is fed regularly (Schoenberg and Trench 1980).

The intracellular localization of the dinoflagellate (Figure 1) raises some key questions about regulation of the symbiosis. First, how does the host recognize, take up, and maintain appropriate symbionts without generating a deleterious immune response that could result in a failure of algal uptake, digestion of the algae after uptake, or apoptosis of the host cells? Second, what metabolites do the two organisms exchange across the symbiosome membrane, and how? It seems likely that the symbiotic state involves both transporters and regulation of metabolic pathways that are distinct from those found in aposymbiotic animals. Third, what changes in transport occur at other membranes? For example, although both gastrodermal and epidermal cells in aposymbiotic anemones presumably excrete ammonium as a toxic waste product, as do other aquatic invertebrates (Wright 1995), at least some of that ammonium must be redirected to the dinoflagellates in symbiotic anemones (Pernice *et al.* 2012). Particularly intriguing questions are how the epidermal tissue layer is nourished (as it lacks both dinoflagellate symbionts and direct access to food particles in the gastric cavity) and whether the nature and mechanisms of this nourishment change upon the establishment of symbiosis.

To investigate these questions, we used RNA-Seq to generate an extensive, annotated transcriptome assembly for symbiotic *Aiptasia*. This transcriptome was then used as a reference to compare global transcript abundances between symbiotic and aposymbiotic anemones. Previous studies have identified few genes that were differentially expressed between the two states, possibly because of the insensitivity of the technologies used, the small fraction of cells that contain dinoflagellates, and/or a lack of probes for the relevant genes. In contrast, we identified nearly 1000 genes with significant expression differences, many of which were large. Many of these expression differences suggest interesting and testable biological hypotheses.

## Materials and Methods

### *Aiptasia* strain and culture

All animals were from clonal population CC7 (Sunagawa *et al.* 2009), which in spawning experiments typically behaves as a male (S. F. Perez and J. R. Pringle, unpublished data). For experiments performed at Stanford, the stock cultures were grown in a circulating artificial sea water (ASW) system at ∼25° with 20 to 40 µmol photons m^−2^ s^−1^ of photosynthetically active radiation on an ∼12-hr light/12-hr dark (12L:12D) cycle and fed freshly hatched brine shrimp nauplii approximately twice per week. To generate aposymbiotic anemones, animals were placed in a separate polycarbonate tub and subjected to several repetitions of the following process: cold-shocking by addition of 4° ASW and incubation at 4° for 4 hr, followed by 1–2 days of treatment at ∼25° in ASW containing the photosynthesis inhibitor Diuron (Sigma-Aldrich D2425) at 50 µM (lighting approximately as noted above). After recovery for several weeks in ASW at ∼25° in the light (as noted above) with feeding (as noted above, with water changes on the days following feeding), putatively aposymbiotic anemones were inspected by fluorescence microscopy to confirm the complete absence of dinoflagellates (whose bright chlorophyll autofluorescence is conspicuous when they are present).

For experiments performed at Cornell, anemones were grown in incubators at 25° in ASW in 1-liter glass bowls and fed (as noted above) approximately three times per week. Symbiotic anemones were kept on a 12L:12D cycle at 18 to 22 µmol photons m^−2^ s^−1^ of photosynthetically active radiation. Aposymbiotic animals were generated by exposing anemones under the same lighting and feeding regimen to 50 µM Diuron in ASW, with daily water changes, for ∼30 d or until the anemones were devoid of algae, as confirmed by fluorescence microscopy. After bleaching, aposymbiotic anemones were maintained in the dark for ∼2 yr (with feeding as noted above) before experimentation.

### Experimental design

Three separate experiments were performed using somewhat different conditions (Table 1). For experiment 1 (RNA-Seq), both symbiotic and aposymbiotic anemones were held at 27° on a 12L:12D cycle, with feeding and water changes as noted above, for 1 month before sampling to allow them to acclimate. The aposymbiotic anemones were checked immediately before sampling by fluorescence microscopy to ensure that they were still symbiont-free. Anemones were collected ∼2 d after the last feeding and ∼5 hr into the light period. Each of three biological replicates per condition consisted of two to five pooled anemones (for a total of ∼35 mg wet weight); samples were stored in RNALater (Ambion AM7021) at −20° until processing.

**Table 1:** Summary of experimental conditions

**Experiment***^a^*	**Site**	**Purpose**	**Light (µmol photons m^−2^ s^−1^)**	**Temperature (°)**	**Feeding schedule**
1, Apo	Stanford	Gene expression*^b^*	25 (12L:12D)	27	Every 2 d
1, Sym	Stanford	Transcriptome assembly and gene expression*^c^*	25 (12L:12D)	27	Every 2 d
2, Apo	Cornell	Gene expression*^d^*	0	25	Unfed 2 wk
2, Sym	Cornell	Transcriptome assembly and gene expression*^e^*	18–22 (12L:12D)	25	Unfed 2 wk
3, Apo	Cornell	RT-qPCR	18–22 (12L:12D)	25	Every 2 d
3, Sym	Cornell	RT-qPCR	18–22 (12L:12D)	25	Every 2 d

a Apo, aposymbiotic anemones; Sym, symbiotic anemones.

b Approximately 49 million 36-bp single-end reads (accession number SRR612167).

c Approximately 200 million 101-bp paired-end reads (accession number SRR610288) and 51 million 36-bp single-end reads (accession number SRR612166).

d Approximately 80 million 101-bp paired-end reads (accession number SRR612165).

e Approximately 83 million 101-bp paired-end reads (accession number SRR696732).

For experiment 2 (RNA-Seq), both symbiotic and aposymbiotic anemones were starved for 2 wk before sampling. Symbiotic anemones were maintained at 25° on a 12L:12D cycle, whereas aposymbiotic anemones were maintained at 25° in constant dark. Anemones were collected 9 hr into the light period of the symbiotic anemones. Four symbiotic or eight aposymbiotic anemones (∼50 mg wet weight in each case) were pooled in each of four biological replicates per treatment, flash-frozen in liquid nitrogen, and held at −80° until processing.

For experiment 3 (RT-qPCR), both symbiotic and aposymbiotic anemones were maintained at 25° on a 12L:12D cycle with feeding every 2 d, followed by water changes; samples were collected 2 d after the last feeding and 6 hr into the light period. Collection and storage of anemones were as in experiment 2.

### RNA isolation and sequencing

In experiment 1, total RNA was extracted from whole anemones using the RNAqueous-4PCR Kit (Ambion AM1914) following the manufacturer’s instructions. The RNA-integrity number (RIN) of each sample was determined using an Agilent 2100 Bioanalyzer, and only samples with a value of nine or more were used. Approximately 3 µg total RNA were processed (including a poly-A^+^-selection step) using the TruSeq RNA Sample Prep Kit (Illumina FC-122-1001) following the manufacturer’s instructions to produce indexed libraries. The resulting libraries were pooled based on their indices (as described in the kit instructions), and clustering and sequencing (both 101-bp paired-end reads and 36-bp single-end reads) were performed by the Stanford Center for Genomics and Personalized Medicine using an Illumina HiSeq 2000 sequencer.

In experiments 2 and 3, total RNA was extracted using the ToTALLY RNA Total RNA Isolation Kit (Ambion AM1910) following the manufacturer’s instructions, except that the RNA was precipitated using 0.1 volume of 3 M sodium acetate and 4 volumes of 100% ethanol. The resulting RNA was purified using the RNA Clean and Concentrator-25 Kit (Zymo Research R1017). For RNA-Seq, the RIN of each sample was verified to be nine or more using an Agilent 2100 Bioanalyzer, and ∼4 µg total RNA per sample were processed using the TruSeq Kit (as noted above) to produce indexed libraries. The resulting libraries were pooled into eight samples per lane, and clustering and sequencing (101-bp paired-end reads) were performed by the Cornell Life Sciences Core Laboratory Center using an Illumina HiSeq 2000 sequencer. Processing of samples for RT-qPCR is described below.

### Read filtering and transcriptome assembly and annotation

Transcriptome assembly used all of the 101-bp paired-end reads obtained from symbiotic anemones at both Stanford and Cornell (Table 1; reads available through the NCBI Short-Read Archive). Before assembly, the reads were processed as follows: (1) reads of <60 bp or containing ≥1 N were discarded; (2) any read for which <25 of the first 35 bases had quality scores >30 was discarded; and (3) reads were trimmed to the first position for which a sliding 4-bp window had an average quality score of <20. The remaining read-pairs were then processed using FLASH (Magoc and Salzberg 2011) to join reads whose ends overlapped by ≥10 bp with no mismatches. Finally, adapter sequencers were removed using cutadapt with the default settings (Martin 2011).

The processed reads were assembled in three sets because of memory constraints. Each set was assembled using an additive-multiple-*k*-mer approach (*k*-mers of 51, 59, 67, 75, 83, 91) with the Velvet/Oases assembler (Velvet version 1.1.07 and Oases version 0.2.02) (Zerbino and Birney 2008; Schulz *et al.* 2012) and merged using the Oases merge function with a *k*-mer of 27. The final outputs of each assembly were merged with one another by again using the Oases merge function. Near-identical contigs (≥99% identical over the length of the shorter contig) were merged using UCLUST version 5.2.32 (Edgar 2010). To cluster alternative transcripts from the same gene (and presumably also transcripts from highly similar paralogs), UCLUST was used again, as follows. Contigs were aligned locally in both directions and clustered together if the alignment consisted of ≥20% of the total length of each contig and the sequence was ≥99% identical over the alignment. These parameters were chosen because they produced valid clusters on a test dataset from zebrafish in 93% of cases (with the remaining cases being mostly the near-identical paralogs common in teleosts because of genome duplication) (E. Lehnert and B. Benayoun, unpublished results).

To assign putative functional roles to the transcripts, we aligned them to the SwissProt protein database and the NCBI nonredundant protein database (nr) using the blastx program from the standalone BLAST 2.2.25+ software suite with an E-value cutoff of 1e−5 (Camacho *et al.* 2009). The results of the alignment to SwissProt were imported using the Blast2GO software package and used to assign Enzyme Codes and Gene Ontology (GO) terms to the predicted proteins (Ashburner *et al.* 2000; Conesa *et al.* 2005).

### Classification of contig origin using a transcript-sorting algorithm and alignment of genomic reads

To classify contigs into those derived from *Aiptasia*, those from the dinoflagellate symbionts, and those from other aquarium organisms that might have been in the gastric cavities or associated with the mucous coats of the isolated anemones, we developed the machine-learning program TopSort, which uses support vector machines to classify transcripts as cnidarian, dinoflagellate, fungal, or bacterial (Burriesci 2011). The basic principle of TopSort is that if there are N features for each element in a dataset, each element can be represented as a point defined by these features in N-dimensional hyperspace. If classes of elements are distinguishable by the N features, then there should be an N-1-dimensional hyperplane that cuts the space such that one class can be separated from the others. If elements are clustered such that they are separable by another shape in N-space (*e.g.*, a hypersphere), then an appropriate transform of the hyperspace to another space will make them separable by a hyperplane. The features used for TopSort were GC content, amino acid and codon biases (where a strong BLAST hit allowed a reliable prediction of reading frame), phylogenetic classification of the top five best BLAST hits to the nr database (scoring each hit as cnidarian, non-cnidarian animal, dinoflagellate, non-dinoflagellate alveolate, plant, fungus, bacteria, or none-of-the-above), and best BLAST hit to a custom database composed of the sequences of known origin that were not chosen for either the training or test set. BLAST hits to the species from which the training and test sequence sets were derived were discarded to avoid the potential development of a classifier that was highly accurate on the test and training sets but useless for a novel dataset.

To build the training and test sets and the custom database, we used publicly available sequences for the cnidarians *Nematostella vectensis* and *Hydra magnipapillata*; the dinoflagellates *Alexandrium tamarense*, *A. catenella*, *A. ostenteldii*, *A. mitum*, *Karlodinium micrum*, *Karenia brevis*, and *Symbiodinium* strain KB8 (clade A); the fungi *Saccharomyces cerevisiae*, *Schizosaccharomyces pombe*, *Aspergillus niger*, and *Neurospora crassa*; and the bacteria *Escherichia coli* and *Salmonella enterica* (see Supporting Information, File S1 for accession numbers). We also included contigs from an earlier aposymbiotic *Aiptasia* transcriptome (Lehnert *et al.* 2012) that had ≥30 reads mapping to them from the aposymbiotic libraries produced during experiment 1 of this study, as well as a large set of contigs from axenically cultured clade B *Symbiodinium* strain SSB01 (Xiang *et al.* 2013; T. Xiang and A. Grossman, personal communication). Sequences from each of the four phylogenetic groups were binned by length (150–300, 300–500, and >500 bp), and equal numbers of sequences from each phylogenetic group were selected randomly from each bin (1076 for 150–300 bp; 2000 for both 300–500; and >500 bp) to make the training set, whereas equivalent numbers were selected to make the test set. The remaining sequences from each phylogenetic group were assigned to the custom database.

In addition, we tested the assembled contigs for alignment to *Aiptasia* genomic DNA sequences. We isolated genomic DNA from aposymbiotic *Aiptasia* and obtained approximately 101 Gb of untrimmed sequence reads from six separate libraries (accession numbers SRR646474 and SRR606428). We aligned the genomic read-pairs to our contigs and obtained the mean count of read-pairs for each contig from the six libraries. A previous test had shown that only 20 of ∼60,000 contigs derived from cultured axenic *Symbiodinium* strain SSB01 (Xiang *et al.* 2013; T. Xiang and A. Grossman, personal communication) had any *Aiptasia* genomic reads mapping to them. However, this clade B strain may have many sequence differences from the clade A strain found in CC7 anemones, so it seemed conceivable that low levels of *Symbiodinium* in the putatively aposymbiotic anemones from which the genomic DNA was derived might lead to misclassification of dinoflagellate transcripts as cnidarian. We determined that 15,499 of the 23,794 contigs classified as dinoflagellate by TopSort had zero genomic reads mapping to them, whereas the median of the mean read counts for the contigs classified by TopSort as cnidarian was ∼200. Thus, we chose a mean read count of 10 as the cut-off to classify a contig as cnidarian by genomic evidence.

### Expression analysis by RNA-Seq

The 36-bp reads (experiment 1) were trimmed as described above. With the 101-bp paired-end reads (experiment 2), the forward reads were shortened to 36 bp for expression analysis and then trimmed as described above. Reads were aligned using bwa (Li and Durbin 2009) to the representative contigs (*i.e.*, the longest contig in each cluster produced by UCLUST; see above). A read was counted toward the total for each contig if it aligned with no errors or gaps to a unique region of the transcriptome. The R package DESeq was used to call contigs as differentially expressed if the false-discovery-rate–adjusted *P* ≤ 0.1 (Anders and Huber 2010).

### Expression analysis by RT-qPCR

RNA was extracted and purified as described above, treated with DNase using the TURBO DNA-free kit (Ambion AM1907) following the manufacturer’s instructions, and diluted to a concentration of 200 ng/µl. cDNA was then synthesized using the GoScript Reverse Transcriptase System (Promega) following the manufacturer’s instructions. Primers (Table S2) were designed using Primer Quest (Integrated DNA Technologies) for 29 contigs with a variety of read counts and expression patterns; four of these contigs had previously been identified as appropriate internal reference standards as described below. The predicted product sizes of 110–238 bp were confirmed by agarose-gel electrophoresis after conventional PCR amplification. Primer efficiencies were determined using Real-time PCR Miner (Zhao and Fernald 2005) and ranged from 90% to 100%. The RT-qPCR products were also sequenced (Cornell Life Sciences Core Laboratory Center), and all matched the expected product identities.

To quantify transcript levels, we used a ViiA 7 thermocycler (Applied Biosystems) with reaction conditions as follows: 12.5 µl of 2× Power SYBR Green Master Mix (Applied Biosystems), 200 nmol of each primer, and 18 ng of cDNA in a total volume of 25 µl. Each sample and a no-template control was run in duplicate with thermocycler parameters of 95° for 10 min, 40 cycles of 95° for 15 sec and 60° for 60 sec, and a subsequent dissociation curve to confirm the absence of nonspecific products. To confirm the absence of genomic-DNA contamination, a pool of all eight RNA samples (see above) was used as template in a separate reaction as described above except omitting the reverse-transcriptase. Real-time PCR Miner was used to calculate the critical threshold (C_T_) of each gene from the raw fluorescence data.

To identify reliable reference standards to use for qPCR normalization, we evaluated six housekeeping genes that appeared to be plausible candidates and have been used for this purpose in previous studies of cnidarian gene expression (File S1). Briefly, the expression levels of these genes were tested across a variety of experimental conditions (*e.g.*, heat shock and cold shock) in both aposymbiotic and symbiotic anemones and evaluated for stability of expression using the software geNorm (Vandesompele *et al.* 2002). Based on this analysis, the genes encoding 60*S* ribosomal protein L11, 40*S* ribosomal protein S7, NADH dehydrogenase subunit 5, and glyceraldehyde-3-phosphate dehydrogenase were selected as standards. The stabilities of these genes in experiment 3 were confirmed using geNorm before calculating a normalization factor from the geometric mean of their expression values (Vandesompele *et al.* 2002) (Table S1). The expression levels of all 29 genes were then normalized via the normalization factor, and relative expression values were calculated using the following equation: 1/(1 + Primer Efficiency)^C_T_. Log_2_ fold-changes in expression in symbiotic relative to aposymbiotic anemones were then calculated as the quotients of the relative expression values from the aforementioned equation. The R software package was used to perform correlations between log_2_ fold-change data from qPCR and RNA-Seq experiment 1.

### Bayesian phylogenetic analysis

Alignments of Npc2 proteins were generated using the MUSCLE software with its default parameters (Edgar 2004). The alignments were inspected to identify regions conserved in all proteins and optimized manually over the conserved regions. We then generated a consensus phylogeny using MrBayes 3.1.2 with the following settings: prset aamodelpr = mixed and lset rates = invgamma (Ronquist and Huelsenbeck 2003). Two separate runs were performed to ensure that identical consensus trees emerged regardless of starting conditions. The runs were terminated after 50 million generations with the average SD of split frequencies ≤0.005.

### Unbiased screening for functional groups among the differentially expressed genes

As one approach to identifying genes involved in the symbiosis, we used the Database for Annotation, Visualization, and Integrated Discovery (DAVID) version 6.7 (Dennis *et al.* 2003; Huang *et al.* 2008). This program performs Fisher exact tests to determine biological processes (based on GO terms) that are significantly overrepresented among differentially expressed transcripts relative to the background transcriptome. The Functional Annotation Clustering method was used, which clusters groups of similar biological processes and provides an enrichment score representative of the −log geometric mean of the *P* values of the individual processes. Clusters were considered significantly enriched when the enrichment score was >1.3 (corresponding approximately to *P* < 0.05).

## Results

### Sequencing and assembly of the transcriptome of symbiotic *Aiptasia*

We isolated total RNA from a clonal population of symbiotic anemones raised under nonstressful culture conditions, enriched for poly-A^+^ RNA, and used this RNA to synthesize paired-end Illumina libraries, from which we obtained a total of ∼345 million pairs of reads containing ∼70 Gb of sequence. The raw reads were trimmed and processed as described in *Materials and Methods*, leaving ∼228 million pairs of reads and ∼45 Gb of sequence. These reads were assembled in three batches using Velvet/Oases and a multiple-*k*-mer approach (see *Materials and Methods*). The resulting assemblies were merged using the Oases merge option, and redundant contigs (≥99% identical over the length of the shorter contig) were collapsed using UCLUST, yielding an initial set of 140,945 contigs with lengths of 102 to 32,510 bp.

To estimate the number of genes represented by these contigs and to choose a representative contig for each gene, we clustered contigs with good alignments (≥99% identical over ≥20% of the length of the shorter contig). This resulted in 52,717 clusters, and the longest contig from each was taken as representative for further analysis (see File S4 for the full set of 52,717 representative contigs). Although 31,014 clusters contained only a single contig, 19,380 contained two to nine contigs, and 2323 clusters contained 10 or more contigs, with a largest cluster of 230 contigs (see *Discussion*).

### Classification of contigs using TopSort and comparison to genomic sequence

It is difficult or impossible to obtain animal RNA without contamination by RNA from the intracellular dinoflagellate symbionts and (although presumably in much smaller amounts) from other organisms in the nonsterile aquarium system. To address this issue, we developed the TopSort support-vector-machines algorithm to classify contigs as putatively of cnidarian, dinoflagellate, bacterial, or fungal origin. Sequences of reliably known origin were used to create training and test sets, and each contig was scored on several metrics (see *Materials and Methods*). After training on the training set, the accuracy of TopSort on the test set was ∼95% for contigs of 150–300 bp and >99% for contigs of >300 bp, for an overall error rate of 2–3%.

We used TopSort to classify the 52,717 representative contigs in our dataset (Table 2, column B). As expected, most contigs were classified as cnidarian or dinoflagellate. However, the 2–3% error rate of TopSort with the test dataset suggested that hundreds of the putatively cnidarian contigs might actually be dinoflagellate contigs that had been misclassified, which would be a significant problem for subsequent analyses of gene-expression differences between symbiotic and aposymbiotic animals. Thus, we also aligned reads from *Aiptasia* genomic-DNA sequence libraries to the transcriptome (see *Materials and Methods*). Approximately 94% of the contigs classified by TopSort as cnidarian had supporting genomic evidence, as compared to only ∼5% of contigs classified by TopSort as noncnidarian (Table 2, columns C–E). These results validated the performance of TopSort in initial classification and yielded a set of 26,219 high-confidence *Aiptasia* contigs (henceforth referred to as “cnidarian”), on which we have focused for our further analyses to date. In the remainder of this article, we also use the term “dinoflagellate” to refer to the 22,668 contigs classified as dinoflagellate by TopSort and lacking matches to *Aiptasia* genomic DNA, and we refer to contigs for which the classifications by TopSort and genomic match conflicted as “ambiguous.”

**Table 2:** Assignment of contigs to species of origin^a^

**A**	**B**	**C**	**D**	**E**
**Type of Organism**	**No. by TopSort****^*b*^**	**No. With Genomic Evidence*****^c^***	**No. Without Genomic Evidence*****^c^***	**False-Positive Rate (%)*****^d^***
Cnidarian	28,026	26,219	1807	6.4
Dinoflagellate	23,794	1126	22,668	4.7
Fungi	166	18	148	10.8*^e^*
Bacteria	731	185	546	25.3*^e^*

a The full list of assignments of individual contigs to their species of origin is provided in File S5.

b See text.

c Genomic evidence was defined as ≥10 paired-end reads aligning from *Aiptasia* genomic DNA libraries prepared from aposymbiotic anemones (see *Materials and Methods*).

d Classified as cnidarian by TopSort but lacking genomic evidence or classified as noncnidarian by TopSort but with apparent matches to *Aiptasia* genomic DNA.

e Many of these are presumably transcripts from contaminants that were present on the surfaces or in the gastric cavity of the anemones from which the genomic DNA was prepared. However, the high rate of apparent false-positives among the putatively bacterial and fungal sequences probably also reflects Bayes’ rule, whereby the ratio of false-positives to true-positives is high when the *a priori* probability of a true-positive is low.

### Characterization and annotation of transcriptome

The cnidarian contigs ranged in size up to >32 kb, with a median of 1644 bp, whereas the dinoflagellate contigs had somewhat smaller maximum and median sizes (Table 3). The remaining contigs (“Other” in Table 3) had a size distribution similar to those of the cnidarian and dinoflagellate contigs. It is therefore unlikely that the failure to classify these contigs as cnidarian or dinoflagellate was simply attributable to their being shorter than average and thus more difficult to annotate by BLAST or to align to genomic reads.

**Table 3:** Size distributions of the representative contigs

**Parameter**	**Cnidarian**	**Dinoflagellate**	**Other*****^a^***
No. of contigs	26,219	22,668	3830
Median contig size (bp)	1644	1144	1474
Mean contig size (bp)	2227	1355	1789
Minimum contig size (bp)	106	108	102
Maximum contig size (bp)	32,510*^b^*	20,508	18,089
Total length of contigs (Mb)	58	31	7

a Includes both the contigs classified as “ambiguous” (see text) and those classified as fungal or bacterial.

b Some of the very long contigs may represent hairpin or chimeric assemblies, a possibility that we have not explored in detail. However, the predicted product of the 32,510-bp contig actually aligns to a portion of titin, a muscle protein that is the largest known protein (Labeit and Kolmerer 1995) and ranges in size from 27,000 to 33,000 amino acids in humans, depending on the splice isoform (Labeit and Kolmerer 1995; Bang *et al.* 2001).

To assign putative functions to the representative cnidarian and dinoflagellate contigs, we used blastx to align them to the SwissProt and NCBI nr databases, retaining only alignments with E-values ≤1e-5. Of the 26,219 cnidarian contigs, 16,373 (62%) had such alignments to 9386 distinct accession numbers in SwissProt (Table 4). In contrast, of the 22,668 dinoflagellate contigs, only 7895 (∼35%) had such alignments to 5054 distinct accession numbers (Table 4). Similar numbers were obtained by aligning sequences to the nr database (Table 4). Using Blast2GO with its default cutoff of 1e−3, we assigned GO terms based on the SwissProt annotations. We were able to assign 10,521 distinct GO terms to cnidarian sequences and 5747 distinct GO terms to algal sequences.

**Table 4:** Summary of alignments to SwissProt and nr databases

**Classification**	**No. of Contigs**	**No. (%) of Contigs Aligned to SwissProt*****^a^***	**No. (%*****^b^*****) of Distinct Accessions**	**No. (%) of Contigs Aligned to nr Database*****^a^***	**No. (%****^b^****) of Distinct Accessions**
Cnidarian	26,219	16,373 (62)	9386 (57)	19,259 (74)	11,593 (60)
Dinoflagellate	22,668	7895 (34)	5054 (64)	11,184 (49)	7789 (70)

a Alignments with E-value ≤1e-5.

b As % of all alignments.

To investigate why there were so few distinct accession numbers relative to the numbers of representative contigs, we examined the distributions of contigs per accession number (Table 5). In both the cnidarian and dinoflagellate cases, ∼76% of accession numbers annotated only one representative contig, and another ∼14% annotated two representative contigs (as might occur with a duplicated gene or two sufficiently different alleles of the same gene). In contrast, some accession numbers were hit by much larger numbers of representative contigs (Table 5). Although there are several possible explanations for such cases (including the existence of extended gene families, complex alternative splicing, and/or somatic differentiation), we suspect that most reflect a failure of contigs derived from the same gene to cluster with the algorithm used, perhaps because of repeat structures within the genes. In any case, if we assume (as a worst-case scenario) that all such cases result from such failures to cluster, and that the failure rate was identical between the successfully annotated and unannotated contigs, then we can infer that the numbers of “unigenes” (sequences derived from distinct genes) present in our dataset are ∼14,500 for *Aiptasia* and ∼14,000 for *Symbiodinium*, representing substantial fractions of the total gene numbers expected from information on other eukaryotes (see *Discussion*).

**Table 5:** Distribution of representative contigs among accession numbers*^a^*

**No. of Contigs With Best Blast Hit to a Given Accession Number**	**No. of Accession Numbers (Cnidarian)**	**No. of Accession Numbers (Dinoflagellate)**
1	7108	3874
2	1332	703
3–5	643	361
6–10	190	85
11–25	89	25
26–50	14	3
>50*^b^*	10	3
Total	9386	5054

a Analysis performed to investigate why there were so few distinct BLAST hits relative to the numbers of representative contigs. See text for details.

b The largest numbers were 187 (cnidarian) and 71 (dinoflagellate).

### Identification of differentially expressed transcripts

To compare gene expression in symbiotic relative to aposymbiotic anemones, we performed two RNA-Seq experiments using somewhat different conditions (see *Materials and Methods* and Table 1). In each experiment, we identified many transcripts that appeared to be differentially expressed, including many in which the changes in abundance were five-fold or more (Table 6, columns B and C). Although the two experiments identified many of the same genes, there were also differences that probably reflect both the noise inherent in such analyses and actual differences in expression attributable to the different experimental conditions. However, we hypothesized that any genes involved directly in the maintenance of symbiosis (*e.g.*, genes encoding proteins found specifically in the symbiosome) would show similar expression differences in both experiments. Therefore, we identified these contigs (Table 6, column D) and focused on them in subsequent analyses.

**Table 6:** Differential expression of cnidarian contigs*^a^*

**A**	**B**	**C**	**D**	**E**
	**No. of Contigs**		**Shared (%*****^d^******^,^******^e^*****)**	
**Contig Behavior*****^b^***	**Experiment 1*****^c^******^,^******^d^***	**Experiment 2*****^c^***	**Total**	**Unannotated**
Upregulated	1109	3093	456 (41)	53 (5)
Upregulated ≥5-fold	138	631	79 (57)	17 (12)^f^
Downregulated	1036	2905	464 (45)	48 (5)
Downregulated ≥5-fold	23	388	6 (26)	4 (17)

a Classified as cnidarian by TopSort and confirmed by genomic match (see Table 2).

b Expression in symbiotic relative to aposymbiotic anemones. In all cases shown, the difference in expression was significant at a false-discovery-rate-adjusted *P* ≤ 0.1.

c For experimental conditions, see *Materials and Methods* and Table 1.

d Complete lists of the 2145 differentially expressed contigs from experiment 1 and the 920 contigs that were differentially expressed in both experiments are provided in the Supporting File S2 and File S3 (note that each spreadsheet contains multiple sheets).

e The percentage in each case is the number shared divided by the number from experiment 1.

f Four of these 17 contigs had ORFs that were >100 codons in length.

Although our further analyses to date have also focused on transcripts with convincing annotations by blastx, it is important to note that 101 of the cnidarian transcripts that appeared to be differentially expressed in both experiments, including 21 with expression changes of five-fold or more, could not be annotated at this time (Table 6, column E). Fifty-two of these transcripts (including four of the 21 with expression changes of five-fold or more) contained apparent open reading frames with ≥100 codons. Identifying the functions of these unknown proteins may be critical to understanding the structural and biochemical bases of the symbiosis.

To evaluate the reliability of the RNA-Seq data, we also performed an RT-qPCR experiment using culture conditions similar to those of experiment 1 (Table 1). We tested 29 contigs that exhibited a range of fold-changes and read counts, including some that were of particular biological interest (Table S1). To assess the overall agreement between the RNA-Seq and RT-qPCR experiments, we determined the Spearman rank correlation coefficient of the log_2_ fold-change for all contigs, excluding those that had apparently infinite changes in expression (*i.e.*, were only found in either symbiotic or aposymbiotic anemones). The correlation coefficient of 0.96 (*P* = 3e−14) showed a strong correlation between the RNA-Seq and RT-qPCR datasets.

In what follows, we discuss several sets of cnidarian genes whose differential expression suggests testable biological hypotheses.

### Genes involved in metabolite transport

Given the intimate relationship between the symbiotic partners, transporters involved in moving metabolites between compartments seem likely to be of special importance in maintaining the symbiosis. To identify such transporters, we screened the differentially expressed transcripts associated with the GO term “P:transport” for those encoding putative transporters of small molecules. Although the GO annotation of *Aiptasia* is incomplete, we were able to identify 48 upregulated and 18 downregulated transcripts encoding putative transporters and transport-related proteins (Table S3). We focus in what follows on the 15 such proteins that were most highly upregulated in symbiotic anemones (Table 7).

**Table 7:** Transporters and transport-related proteins that were strongly upregulated in symbiotic anemones*^a^*

**Line**	**Fold-change*****^b^***	**Fold-change*****^c^***	**Locus/Transcript**	**Best Blast Hit**	**UniProt Accession No.**	**Blast-hit E-Value**
1	11	6.3	86800/1	Human facilitated glucose transporter (GLUT8)	Q9NY64	9e−89
2	3.7	ND	11708/1	Human facilitated glucose transporter (GLUT8)	Q9NY64	1e−88
3	∞	ND	36456/1	Rabbit Na^+^/(glucose/*myo*-inositol) transporter 2	Q28728	3e−104
4	5.8	ND	45451/1	*Drosophila* lipid droplet surface-binding protein 2	Q9VXY7	2e−08
5	28	3.7	77179/1	Human scavenger receptor class B member 1	Q8WTV0	9e−65
6	44	57	125065/1	*Drosophila* organic cation (carnitine) transporter	Q9VCA2	6e−35
7	600	26	102514/1^d^	Human Npc2 cholesterol transporter	P61916	2e−14
8	∞	29	58798/1	Bovine Na^+^-dependent and Cl^−^-dependent taurine transporter	Q9MZ34	1e−169
9	4.9	6.2	95114/1	Mouse aromatic-amino-acid transporter 1	Q3U9N9	3e−65
10	6.9	ND	12006/1	*Xenopus* GABA and glycine transporter	Q6PF45	8e−60
11	4.3	ND	84720/1	Fish (*Tribolodon*) carbonic anhydrase II	Q8UWA5	2e−36
12	13	2.2	65589/1	Sheep aquaporin-5	Q866S3	8e−37
13	4.3	ND	2130/2	Pig aquaporin-3	A9Y006	1e−68
14	130	ND	60777/1	Zebrafish NH_4_^+^ transporter rh type b	Q7T070	3e−98
15	5.9	7.0	70728/1	*C. elegans* NH_4_^+^ transporter 1 (AMT1-type)	P54145	6e−72

a Putative small-molecule transporters and some proteins of related function are listed in the order of their discussion in the text.

b By RNA-Seq (see Table S3). The arithmetic mean of the values from experiments 1 and 2 is shown except for transcript 77179/1 (line 5). ∞, expression was not detected in aposymbiotic animals. Transcript 77179/1 was detected in aposymbiotic anemones in experiment 1 but not in experiment 2, giving a nominal ∞-fold change in expression in that experiment. However, because the normalized read counts in both experiments were rather low, and because the possible involvement of the 77179/1-encoded protein in lipid metabolism makes it possible that its expression level was affected by the starvation conditions used in experiment 2, we indicate here the more conservative value from experiment 1 alone.

c By qPCR (see Table S1); ND, not determined.

d Encoding putative protein Npc2D (Figure 2 and Figure S1).

#### Transport of photosynthetically fixed carbon and other organic metabolites:

Among the transcripts strongly upregulated in symbiotic anemones were two (Table 7, lines 1 and 2) that encode proteins closely related (∼39% identify in amino acid sequence) to the mammalian facilitative glucose transporter GLUT8, which localizes to the endosome membrane (Augustin *et al.* 2005). This localization depends on a dileucine motif near the N-terminus, and indeed dileucines are present at amino acids 32–33 and 26–27 of the two *Aiptasia* Glut8 proteins. One or both of the *Aiptasia* Glut8 proteins thus are likely to be involved in the transport of photosynthetically produced glucose across the symbiosome membrane into the host cytoplasm (see *Discussion*). However, it should also be noted that the transcript encoding a predicted Na^+^-glucose/*myo*-inositol co-transporter was detected only in symbiotic anemones (Table 7, line 3), and a transcript encoding a related protein was also upregulated 2.2-fold (Table S3, line 26). Interestingly, a transcript encoding a third member of this protein class was strongly downregulated in symbiotic anemones (Table S3, line 65).

Lipids may also be an important energy currency in symbiotic animals (see further discussion below), and in this regard, it is interesting that the transcripts for a putative lipid-droplet surface-binding protein (potentially involved in the mobilization of stored fats for transport), a protein similar to scavenger receptor class B member 1 (related to CD36-type fatty acid transport proteins), and a putative carnitine transporter (potentially involved in entry of fatty acids into mitochondria for degradation) were all strongly upregulated in symbiotic animals (Table 7, lines 4–6). In the last regard, it should also be noted that the transcripts for several putative acyl-carnitine transferases were also upregulated in symbiotic anemones (Table S3, lines 25, 43, and 44).

Also dramatically upregulated was the transcript for a member (Npc2D) of the Npc2 protein family (line 7 in Table 7 and Figure S1A). In mammalian and *Drosophila* cells, Npc2 binds cholesterol in the lumen of the endosome and lysosome and transfers it to Npc1, a transmembrane protein that exports the cholesterol to other intracellular locations (Frolov *et al.* 2003; Sleat *et al.* 2004; Huang *et al.* 2007; Infante *et al.* 2008). Consistent with a previous study of the anemone *Anemonia viridis* (Ganot *et al.* 2011), we identified multiple transcripts encoding Npc2-like proteins in the *Aiptasia* transcriptome and in the transcriptomes of three other cnidarians. A multiple-sequence alignment and Bayesian phylogenetic analysis identified two subclades, suggesting that there has been at least one duplication event in the Anthozoan lineage (Figure 2A). One subclade (including the Npc2A proteins of both *A. viridis* and *Aiptasia*) clustered with the canonical Npc2 proteins found in most animals (including mammals and *Drosophila*), whereas the second subclade contained both the *A. viridis* (Sabourault *et al.* 2009; Ganot *et al.* 2011) and *Aiptasia* Npc2D proteins that are upregulated during symbiosis. Strikingly, all of the proteins in this second subclade have sequence alterations at conserved positions in the sterol-binding site (Figure 2B). Mutations to alanine at these positions are known to disrupt cholesterol binding in human cells (Ko *et al.* 2003; Wang *et al.* 2010), raising interesting questions about the roles of these proteins in symbiotic cnidarians (see *Discussion*).

**Figure 2:**
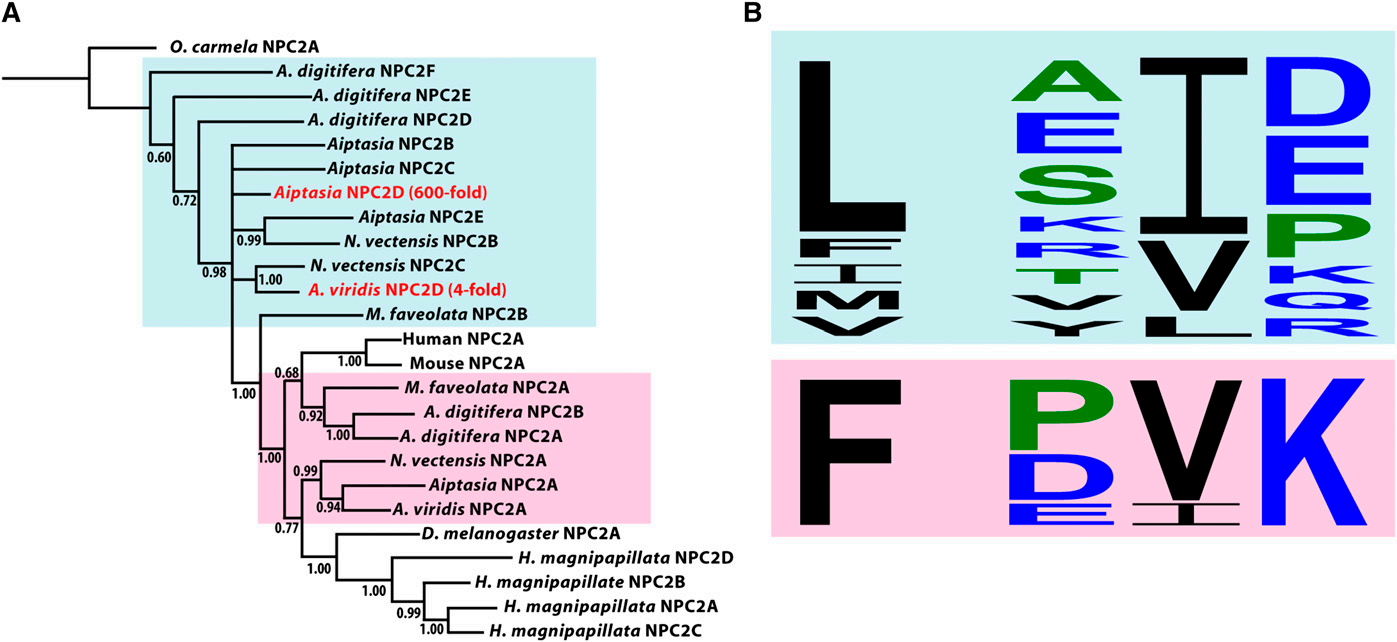
Npc2-like proteins that putatively do or do not have the ability to transport cholesterol. (A) Consensus phylogenetic tree constructed from alignments (Figure S1B) of 25 Npc2-like proteins (see *Materials and Methods*). *Oscarella carmela*, a sponge, served as the outgroup, and the single human Npc2 protein, the single mouse Npc2 protein, and one of the eight *Drosophila melanogaster* Npc2 proteins were included in the analysis. The cnidarian sequences included are from two corals (*Acropora digitifera* and *Montastraea faveolata*), three anemones (*Aiptasia* sp., *Nematostella vectensis*, and *Anemonia viridis*), and a hydrozoan (*Hydra magnipapillata*). The Npc2-encoding transcripts found to be upregulated in symbiotic anemones, which fall outside the clade containing the mammalian and *Drosophila* sequences, are shown in red with their fold-changes (Table 7, line 4; Ganot *et al.* 2011). Light blue and pink shading indicate the groups of anthozoan proteins in the cladogram to which the sequence displays in (B) correspond. Numbers indicated the bootstrap values for the branches indicated. (B, lower) Amino acids highly conserved in animal (including some cnidarian) Npc2 proteins and thought to be involved in cholesterol binding (see text and Figure S1A). The mammalian proteins both have the sequences F…PVK, and the *Drosophila* Npc2A sequence is F…PVL. (B, upper) The variety of amino acids found at the corresponding positions in members of the other protein clade. The differentially regulated *Aiptasia* and *A. viridis* Npc2D proteins both have the sequence L…SID.

Other putative organic-metabolite transporters also showed large changes in expression. In particular, a transcript encoding a putative taurine transporter was detected only in symbiotic anemones (Table 7, line 8), whereas the transcripts for an aromatic-amino-acid transporter and a GABA/glycine transporter were upregulated 4.9-fold and 6.9-fold, respectively (Table 7, lines 9 and 10). Interestingly, taurine has been reported to comprise ∼35% of the amino-acid pool in symbiotic *Aiptasia* (Swanson and Hoegh-Guldberg 1998), although its specific functions are not well-understood. Determining the intracellular localization of the transporter identified here could provide insight into the possible function(s) of taurine. The transcripts for other putative amino-acid transporters also showed significant differences in expression between symbiotic and aposymbiotic anemones (Table S3, lines 16, 28, 30, 34, 39, 40, 45, 52, and 55), suggesting that the establishment of symbiosis produces profound changes in amino-acid transport and metabolism (see *Discussion*).

#### Transport of inorganic nutrients:

CO_2_ is an excreted waste product for animals such as aposymbiotic anemones, but it is required for photosynthesis when dinoflagellate symbionts are present. It may not require specific transporters if it can diffuse freely across cellular membranes. However, to maintain a high concentration of inorganic carbon in the symbiosome, the host may need to convert CO_2_ to the less freely diffusing bicarbonate anion (Bertucci *et al.* 2013). We identified one carbonic-anhydrase gene that was upregulated 4.3-fold in symbiotic anemones (Table 7, line 11), while a second gene was downregulated three-fold (Table S3, line 63). In addition, it is not clear that CO_2_ diffuses sufficiently rapidly through the relevant membranes to support efficient photosynthesis, and some studies have suggested that aquaporins may play a role in facilitating this diffusion (Kaldenhoff 2012; Uehlein *et al.* 2012). Thus, it is of interest that that we found two aquaporins to be upregulated 13-fold and 4.3-fold in symbiotic anemones (Table 7, rows 12 and 13).

Although aposymbiotic anemones, like other aquatic animals, excrete excess (and potentially toxic) ammonium produced by amino acid breakdown (Wright 1995), symbiotic anemones need to supply nitrogen to their dinoflagellates (Pernice *et al.* 2012). Thus, it was not surprising that we found differentially expressed genes encoding ammonium transporters. These genes were in both of the two major families found in animals: a “rhesus-like” gene and an “AMT-like” gene were upregulated 130-fold and 5.9-fold, respectively, in symbiotic anemones (Table 7, rows 14 and 15), suggesting that they might be involved with ammonium supply to the dinoflagellate, whereas another rhesus-like transporter was downregulated 2.9-fold (Table S3, row 61), suggesting that it might be involved in ammonium excretion.

The host must also supply other inorganic nutrients to the dinoflagellates. For example, phosphate and sulfate must be translocated across the symbiosome membrane either as the inorganic ions or as parts of some organic metabolites. In this regard, it is of interest that we found the genes for two putative inorganic-phosphate transporters to be upregulated approximately two-fold in symbiotic anemones, a gene for a putative UDP-sugar transporter to be upregulated 2.7-fold, and a gene for a putative sulfate transporter to be upregulated 3.1-fold (Table S3, lines 19, 22, 27, and 42). In addition, although it is not clear why, zinc is apparently absorbed to a greater extent by symbiotic than aposymbiotic anemones (Harland 1990), with increased concentrations in both animal and dinoflagellate, and we found genes for three putative zinc transporters, in two different families, to be upregulated 1.7-fold to 2.6-fold (Table S3, rows 23, 36, and 46).

### Genes controlling certain metabolic pathways

To explore the integration of metabolite transport with the overall regulation of metabolic pathways, we looked for the presence and coordinated regulation of genes encoding the enzymes of particular pathways that we hypothesized might be involved in the animal’s response to the presence of a symbiont. For these analyses, we used the full transcriptome but only the expression data from RNA-Seq experiment 1, because the starvation of the anemones in experiment 2 seemed likely to have had a strong effect on the expression of metabolic-pathway genes.

#### Lipid metabolism:

**Figure 3:**
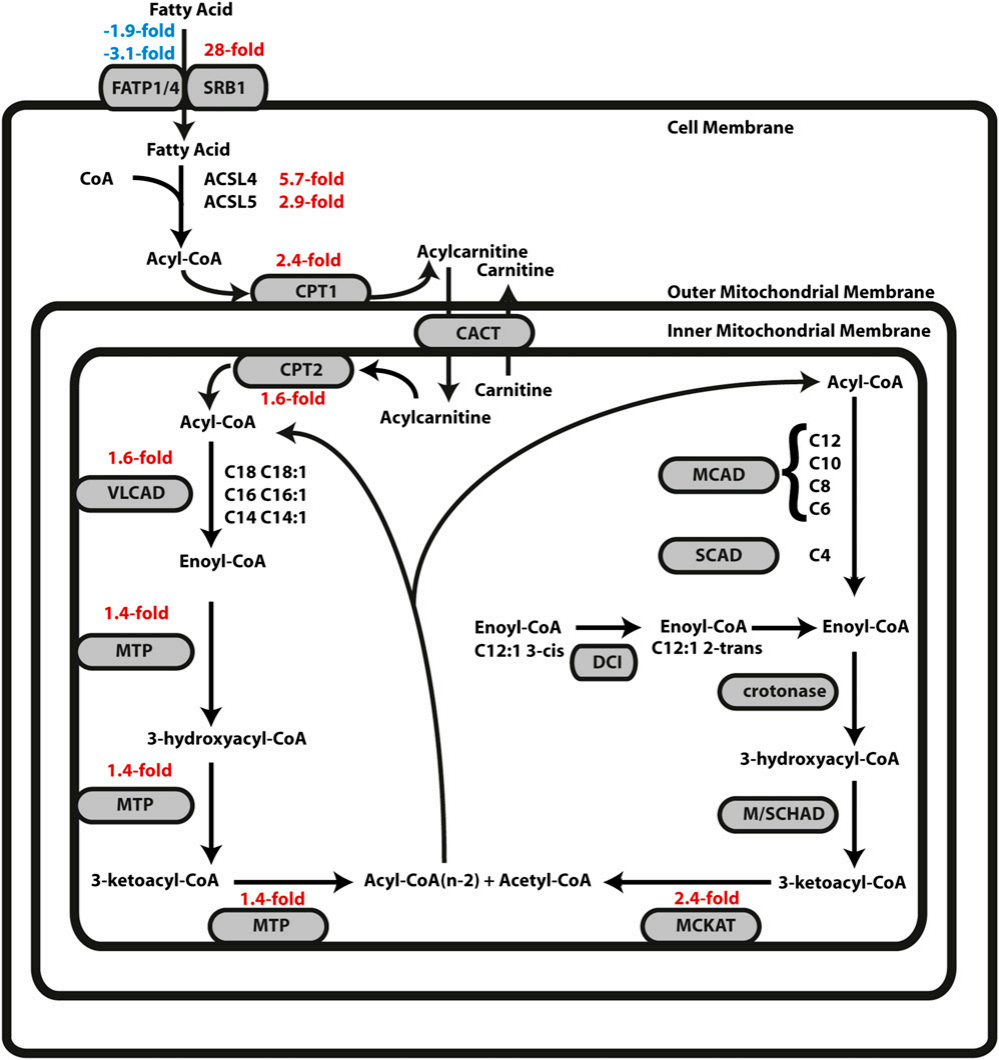
Expression changes of genes governing β-oxidation of fatty acids. The diagram (adapted from Houten and Wanders 2010) shows the localization of proteins involved in fatty-acid transport and β-oxidation in relation to the membranes of the mitochondrion and cell (as known from other animal cells). Statistically significant expression changes from RNA-Seq experiment 1 are shown when applicable; upregulation in symbiotic relative to aposymbiotic anemones is shown by positive/red numbers, and downregulation is shown by negative/blue numbers. Scavenger receptor class B member 1 (SRB1; CD36-related protein) and FATP1/4, possible fatty-acid transporters at the cell surface; ACSL4 and ACSL5, enzymes that convert free fatty acids to fatty acyl-CoA esters; CPT1, CPT2, and CACT, proteins involved in transporting fatty acyl-CoA esters across the mitochondrial membranes; VLCAD, MTP, MCAD, SCAD, M/SCHAD, and MCKAT, enzymes responsible for β-oxidation; DCI, converts fatty acids with double bonds starting at odd-numbered positions to fatty acids with double bonds starting at even-numbered positions; crotonase, hydrates double bonds that start at even-numbered positions. See Table S4, lines 14–30, for full protein names, UniProt accession numbers, and transcript numbers.

There appear to be systematic changes in lipid metabolism between symbiotic and aposymbiotic anemones. Four genes encoding enzymes involved in fatty-acid synthesis (acetyl-CoA carboxylase, a fatty-acid elongase, and ∆^5^- and ∆^6^-fatty acid desaturases) were upregulated 3.5-fold to 6.2-fold (Table S4, lines 1–4), and at least nine genes encoding proteins putatively involved in lipid storage or its regulation were also differentially regulated (Table S4, lines 5–13). In addition, many genes involved in β-oxidation of fatty acids were upregulated in symbiotic anemones (Figure 3 and Table S4, lines 17–21, 23, 24, and 30). Although some of the fold-changes were not large, the consistency is striking, and gastrodermal and epidermal cells may well differ in their expression patterns in ways that obscure the full extent of the changes in a particular cell population (see *Discussion*). Finally, although the glyoxylate cycle (which allows cells to achieve a net synthesis of longer carbon chains from two-carbon units such as those derived by β-oxidation) is not generally present in animal cells, we identified genes putatively encoding its two key enzymes, isocitrate lyase and malate synthase (Table S4, lines 31 and 32), consistent with a previous report of the presence of this cycle in cnidarians (Kondrashov *et al.* 2006). Although the malate-synthase transcript showed no statistically significant differential expression, the isocitrate-lyase transcript was upregulated 3.9-fold in symbiotic anemones. Interestingly, we did not see significant upregulation of the genes encoding the enzymes responsible for metabolizing medium-chain and short-chain fatty acyl-CoA (MCAD, SCAD, crotonase, and M/SCHAD in Figure 3 and Table S4, lines 25–28), suggesting that the metabolic change accompanying the establishment of symbiosis primarily involves long-chain and/or very-long-chain fatty acids.

#### Amino-acid metabolism and the SAM cycle:

**Figure 4:**
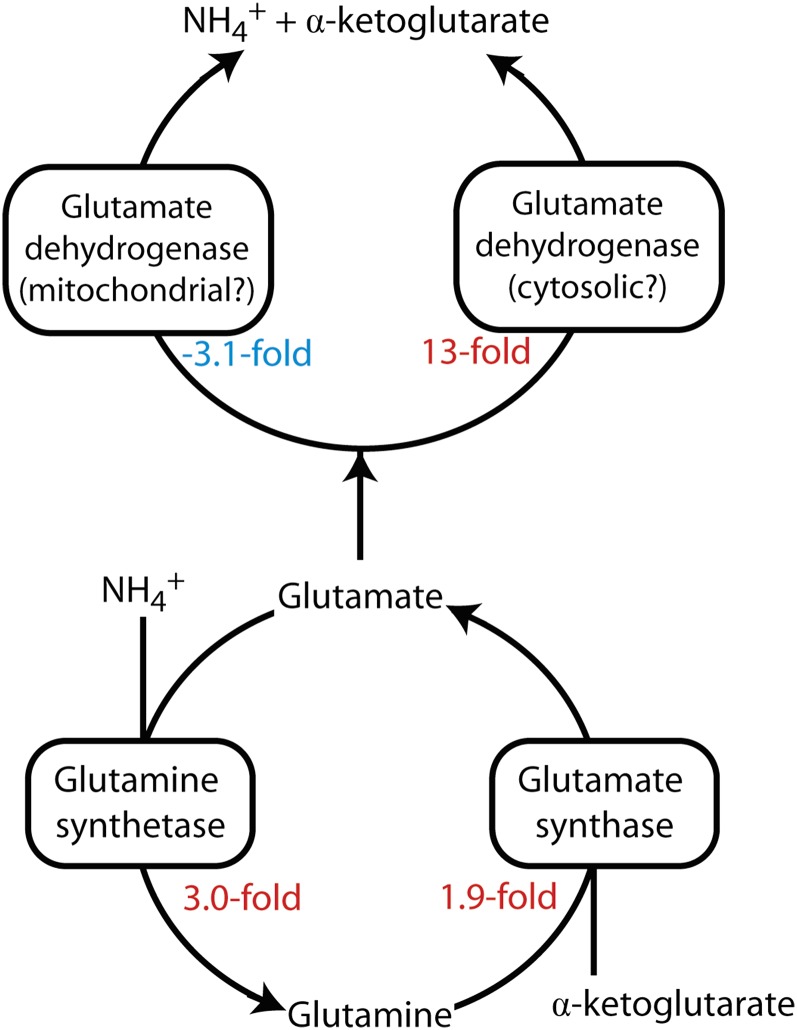
Expression changes of genes governing glutamine and glutamate metabolism. Upregulation in symbiotic relative to aposymbiotic anemones is shown by positive/red numbers, and downregulation is shown by negative/blue numbers. For UniProt and transcript numbers, see Table S5, lines 1–4. The possible localizations of the glutamate dehydrogenases are discussed in the text.

Consistent with previous observations (Wang and Douglas 1998), we found that the transcripts for both a glutamine synthetase and an NADPH-dependent glutamate synthase were upregulated in symbiotic anemones (Figure 4), suggesting that the epidermal cells, the gastrodermal cells, or both synthesize glutamate via a complete GS-GOGAT cycle (Miflin and Habash 2002) rather than (or in addition to) simply obtaining it from the dinoflagellate. We also identified both upregulated and downregulated transcripts encoding glutamate dehydrogenases (Figure 4), which normally catabolize glutamate to α-ketoglutarate and ammonium in animal cells (where the concentrations of ammonium are typically too low to allow the reverse reaction to proceed effectively). The subcellular localization program WoLF PSORT (Horton *et al.* 2007) predicts that the downregulated and upregulated enzymes should localize to the mitochondria and cytosol, respectively, consistent with a previous report that corals contain both mitochondrial and cytosolic glutamate dehydrogenases (Dudler *et al.* 1987). It seems likely that these initially rather puzzling observations (upregulation of one glutamate dehydrogenase and downregulation of another; upregulation of enzymes both of glutamate synthesis and of glutamate breakdown) reflect the differing metabolic needs of different cell types, and/or of different compartments within the same cells, in symbiotic anemones.

**Figure 5:**
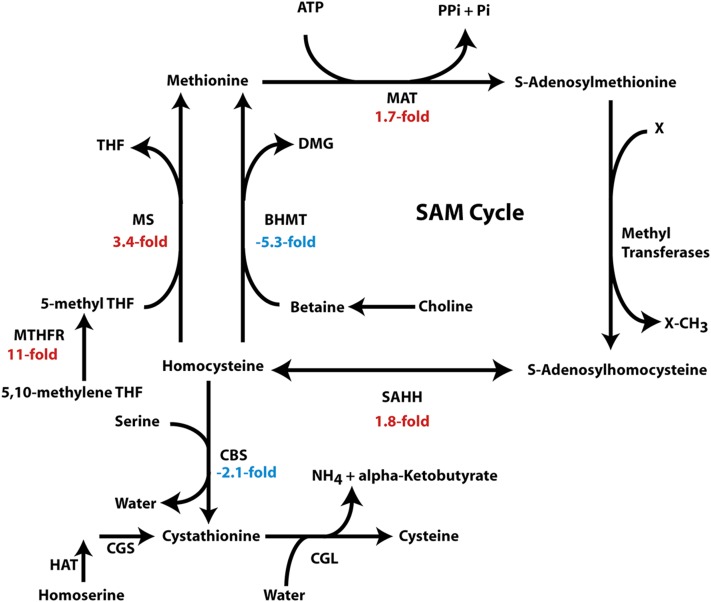
Expression changes of genes governing the metabolism of sulfur-containing amino acids and the *S*-adenosylmethionine (SAM) cycle. Upregulation in symbiotic relative to aposymbiotic anemones is shown by positive/red numbers, and downregulation is shown by negative/blue numbers. For full names of enzymes, UniProt accession numbers, and transcript numbers, see Table S5, lines 7, 8, 10–14, 18, and 19. THF, tetrahydrofolate; DMG, dimethylglycine.

We also observed multiple changes in the expression of genes governing the metabolism of sulfur-containing amino acids and the *S*-adenosylmethionine (SAM) cycle (Figure 5). Based on the failure to find a gene encoding cystathionine β-synthase (CBS) in the *A. digitifera* genome, it was hypothesized that cysteine is an essential amino acid in cnidarians that must be obtained directly from either prey or the symbiont (Shinzato *et al.* 2011). However, we found an *Aiptasia* transcript encoding a CBS (best BLAST hit, rabbit CBS, Q9N0V7; E-value 9e−166), suggesting that anthozoans resemble other animals in their ability to synthesize cysteine from methionine. The CBS transcript was downregulated 2.1-fold in symbiotic anemones, perhaps reflecting a decreased need for cysteine synthesis in the host because it is being supplied directly by the dinoflagellate. Conceivably in more obligately symbiotic corals, the enzyme is never needed and the gene has been lost altogether. It should also be noted that cysteine synthesis via the CBS pathway is a drain on the homocysteine pool, which otherwise remains available for the synthesis of methionine and the SAM cycle. The concordant upregulation of four genes encoding enzymes of the SAM cycle (Figure 5) suggests that it may assume an increased importance in symbiotic animals, although the very wide range of possible methylation targets makes it difficult to guess at the precise biological significance of this regulation. The apparent switch of pathways used for synthesis of methionine from homocysteine (Figure 5) may also reflect an alteration in the kinetics and/or localization of the SAM cycle.

Interestingly, despite the presence of a CBS, we could not find a gene(s) encoding aspartokinase or homoserine dehydrogenase in the transcriptomes of symbiotic or aposymbiotic *Aiptasia* or in the *A. digitifera* genome (Shinzato *et al.* 2011) (Table S5, lines 15–17), implying that anthozoans would be unable to achieve a net synthesis of homoserine (and hence of homocysteine and other sulfur-containing amino acids) from central metabolic intermediates. If confirmed, this would be consistent with the situation in other animals (in which methionine is an amino acid essential in the diet) (Guedes *et al.* 2011) but surprisingly inconsistent with labeling results indicating synthesis of methionine by starved aposymbiotic anemones (Wang and Douglas 1999). A related puzzle is that the *Aiptasia* transcriptome and the *A. digitifera* genome appear to contain genes encoding both a homoserine *O*-acetyltransferase and a cystathionine γ-synthase, which would allow the synthesis of cystathionine from homoserine (Figure 5), but not a cystathionine β-lyase, which in many microorganisms is responsible for the synthesis of homocysteine from cystathionine (Table S5, lines 18–20). Further studies will be needed to resolve these issues.

These questions about methionine and cysteine metabolism raised a broader question about the degree to which the amino-acid–biosynthetic capabilities of anthozoans resemble those of better-characterized animals, in which 12 of the 20 amino acids needed for protein synthesis cannot be synthesized from central-pathway intermediates and so must be obtained (directly or indirectly) from the diet. To address this question, we asked if the elements of amino-acid–biosynthetic pathways were present in the *Aiptasia* transcriptome. As expected, it appears that *Aiptasia* should be able to synthesize the eight generally nonessential amino acids from intermediates in the central metabolic pathways (Table S5, lines 1, 2, 22–36). In addition, like other animals, they should be able to synthesize arginine from ornithine via the urea cycle (Table S5, lines 37–42), although a net synthesis of ornithine and arginine would not be possible because of the apparent lack of either an acetylglutamate kinase or an ornithine acetyltransferase (Table S5, lines 43 and 44). Similarly, although the *Aiptasia* transcriptome revealed genes encoding various enzymes involved in interconversions within other groups of amino acids, key enzymes needed to synthesize these groups of amino acids from central-pathway intermediates appear to be missing (Table S5, lines 15–17, 45–79). Thus, *Aiptasia*, like other animals, apparently must obtain 12 amino acids (or their amino-acid precursors) from their food, their dinoflagellate symbionts, or both. This conclusion is generally compatible with radiolabeling studies suggesting that leucine, isoleucine, valine, histidine, lysine, phenylalanine, and tyrosine are all translocated from the dinoflagellates to the host (Wang and Douglas 1999).

### Genes potentially involved in host tolerance of dinoflagellates

To take an unbiased approach to the identification of other genes that might be involved in maintenance of the symbiosis, we used the DAVID program to identify biological processes (based on GO terms) that were significantly overrepresented among the differentially expressed transcripts (see *Materials and Methods*). Among the groups of genes identified in this way were three that are potentially involved in the animal host’s tolerance of the symbiotic dinoflagellates.

#### Response to oxidative stress:

**Figure 6:**
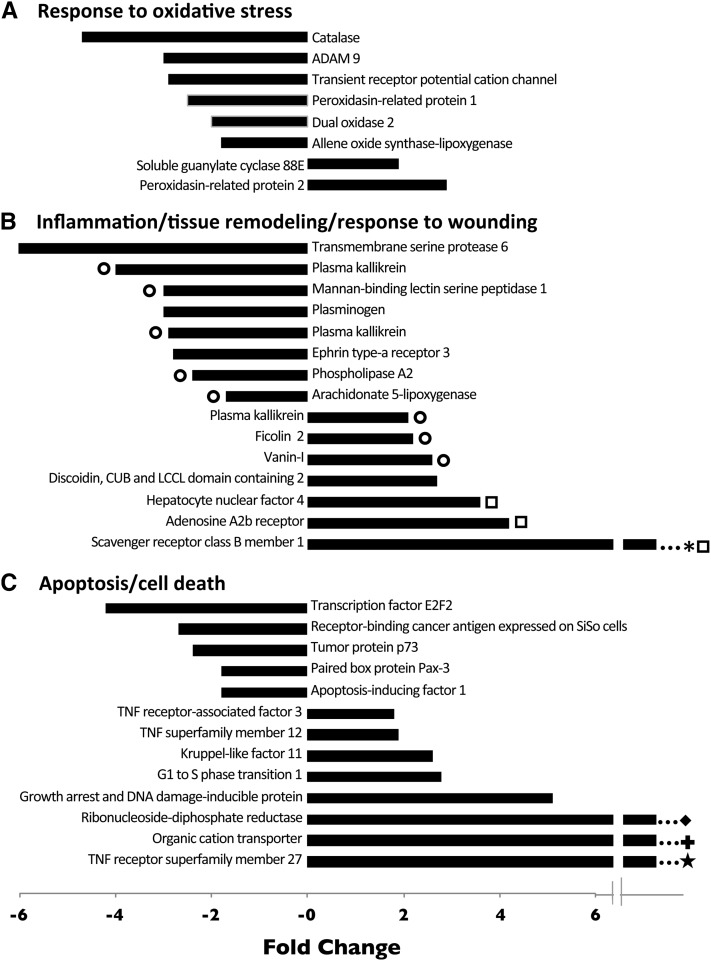
Expression changes of genes with functions that may relate to host tolerance of the symbiont. Functionally related groups of genes (by GO term assignments) that were significantly enriched among the differentially expressed genes relative to the background transcriptome were identified as described in the text. Fold-changes are shown as expression in symbiotic anemones relative to that in aposymbiotic anemones. ○, putatively pro-inflammatory; □, putatively anti-inflammatory; *, highly upregulated (28-fold in experiment 1 and detected only in symbiotic animals in experiment 2; see also Table S3, footnote b); ◆, 12-fold change; **+**, 44-fold change; ★, 60-fold change. For additional details, see Table S6.

Because the presence of an intracellular photosynthetic symbiont presumably imposes oxidative stress on an animal host (see *Discussion*), it was quite surprising that of the eight differentially expressed genes identified under this GO term, six (including a catalase gene) were actually downregulated in symbiotic animals (Figure 6A). Moreover, one of the two upregulated genes encodes a predicted guanylate cyclase, which may have many functions unrelated to oxidative stress. The other upregulated gene is one of a pair encoding distinct *Aiptasia* proteins (Figure S2A) that had a human peroxidasin as their top blastx hit (Table S6, section A). However, a function for these proteins in coping with oxidative stress is doubtful for two reasons. First, the second gene is downregulated in symbiotic animals (Figure 6A). Second, despite the blastx results, neither of the *Aiptasia* proteins contains the peroxidase domain found in canonical peroxidasins with peroxidase activity (Nelson *et al.* 1994) (Figure S2, A and B)

#### Inflammation, tissue remodeling, and response to wounding:

Of the 15 differentially expressed genes associated with this cluster of GO terms, eight were downregulated and seven were upregulated in symbiotic animals (Figure 6B). Despite this heterogeneity, a suggestive pattern was observed in which genes encoding proteins whose homologs are considered pro-inflammatory were mostly downregulated, whereas the three genes encoding proteins whose homologs are considered anti-inflammatory were all upregulated (Figure 6B and Table S6). The pattern appears even stronger when it is noted that one of the three upregulated genes with a putatively pro-inflammatory function encodes just one of at least three distinct *Aiptasia* plasma-kallikrein homologs (Figure S2C), the other two of which are downregulated (Figure 6B), and that the upregulated ficolin may function specifically in recognition of *Symbiodinium* (Logan *et al.* 2010) rather than just as a general activator of the complement innate-immunity pathway. Thus, a downward modulation of the host’s inflammatory response may contribute to allowing the persistence of dinoflagellate symbionts.

#### Apoptosis and cell death:

Of the 13 differentially expressed genes associated with this pair of GO terms, five were downregulated in symbiotic animals but eight were upregulated, including several with large fold-changes in expression (Figure 6C). Thus, it seems possible that an increased activity of cell-death pathways may be required for the animal to cope with the presence of the symbiotic dinoflagellate even under conditions considered to be nonstressful (see *Discussion*).

## Discussion

To explore the cellular and molecular basis of the cnidarian–dinoflagellate symbiosis, we undertook a global analysis of the transcriptomes of symbiotic and aposymbiotic *Aiptasia*. This study has yielded (1) extensive transcriptome assemblies that should be of great value for a wide variety of future studies; (2) testable hypotheses about changes in metabolism and metabolite transport that may occur in the host on symbiosis establishment; and (3) novel information and hypotheses about genes that may be involved in symbiont recognition and tolerance by the host. The transcriptomes also provide a reference for future studies of gene expression under other conditions such as exposure to various stresses.

### Transcriptome assembly and annotation

We have sequenced, assembled, and partially characterized the transcriptomes of both a clonal stock of symbiotic *Aiptasia* and the endogenous clade A symbionts present in that stock; this same stock was also used in previous, less extensive transcriptome studies (Sunagawa *et al.* 2009; Lehnert *et al.* 2012). The animal and algal transcripts were separated bioinformatically using the TopSort algorithm, which we developed for this purpose, and comparisons to genomic sequence obtained from fully aposymbiotic animals. Although the assemblies appear to be of high quality overall, there remain some areas where improvements could be made. For example, it remains unclear both why reads from some genes assembled into multiple contigs that clustered based on regions of nucleotide identity (up to 230 contigs in a cluster) and why a few accession numbers have so many representative transcripts aligning to them. Because it seems unlikely that alternative splicing and gene duplications alone could explain the magnitude of the effects observed, we presume that they result from some combination of these factors and the inherent complexities of *de novo* transcriptome assembly. For example, two of the contig clusters with the most members encode putative actins and olfactory C proteins. In most animals, actins are encoded by families of genes and expressed at high levels, whereas olfactory C proteins are members of very large gene families. Highly abundant transcripts may have some error-containing reads that recur with sufficient frequency that they are assembled into distinct contigs, and gene families with many members could generate chimeric contigs if they have identical subsequences that are longer than the *k*-mers used for assembly. In addition, templates derived from more than one gene may arise during the reverse-transcription or PCR steps of library preparation; the resulting fusion reads may lead to incorrect assembly of contigs, as well as increase the proportion of such contigs substantially when the Oases merge function is used [from 3.6% to 12.2% in one reported case (Chu *et al.* 2013)]. The question about accession numbers can perhaps be explained by similar mechanisms. One approach that might help with these problems would be to assemble the reads initially with a greater *k*-mer length and coverage cutoff to obtain fewer misassemblies for the most abundant transcripts and gene families, remove the reads that map to these transcripts, and then assemble the remaining lower-coverage reads with less stringent thresholds.

The assemblies could also be improved by investigating more closely the contigs for which the species of origin could not be determined with the methods used to date. To this end, the performance of TopSort could probably be improved in one or more of several ways. First, it could be retrained with a new dataset that includes transcriptome or genome sequence from an axenic clade A *Symbiodinium* strain (to improve recognition of contigs from the clade A strain resident in *Aiptasia* stock CC7). Second, it could be extended such that it assigns contigs not just to the four groups used to date (cnidarian, dinoflagellate, fungi, and bacteria) but also to other groups (such as diatoms and ciliates) that may be present in nontrivial amounts in the guts and/or mucous layers of the anemones; this would also require retraining with a dataset that included unequivocal sequences from those groups. Third, the classification metrics could be extended to include other sequence features found to be specific to the phyla of interest [such as the spliced leader sequences thought to be present in many dinoflagellate transcripts (Zhang *et al.* 2007)]. In addition to improving the performance of TopSort, its assignments could also be tested further using alignment to transcriptome or genome sequence from a clonal, axenic *Symbiodinium* strain (preferably of clade A), essentially as we have already done using *Aiptasia* genome sequence. Ultimately, however, some contigs may remain ambiguous in assignment until assembled genomes of both partner organisms are available for alignment.

Several additional issues will require further investigation as the relevant resources become available. First, the numbers of unigenes found here for both symbiotic partners (∼14,000 by conservative estimate) are significantly less than those expected for the full genomes. For *Symbiodinium*, this probably reflects, at least in part, the existence of many genes that are expressed at significant levels only in free-living and/or stressed organisms. For *Aiptasia*, it presumably reflects the absence in the current transcriptome of genes that are expressed at significant levels only in specialized and nonabundant cell types (*e.g.*, in nerve and muscle), at other stages in development (*e.g.*, in embryos and larvae), or under other environmental conditions. Second, only 62% and 74% of representative *Aiptasia* transcripts could be annotated using the SwissProt and nr databases, respectively, and these numbers were even lower (35% and 49%) for *Symbiodinium*. This incomplete annotation presumably reflects the poor representation in the databases of genes and proteins unique to these relatively understudied organisms, as well as the great phylogenetic distance of the dinoflagellates from more intensively studied groups. Finally, the *Symbiodinium* transcriptome reported here has not yet been analyzed in depth. However, we should soon be able to use this transcriptome to make interesting comparisons of gene expression in this *Symbiodinium* strain growing in culture *vs. in hospite*, in this strain after exposure to various stressors, and in different *Symbiodinium* strains grown in this same host, as well as to make informative comparisons to the genomic and transcriptomic sequences that are currently becoming available for other *Symbiodinium* types (Bayer *et al.* 2012; Shoguchi *et al.* 2013).

### Differential expression of animal genes

Based on their consistent behavior in two separate RNA-Seq experiments performed under somewhat different conditions, ≥920 genes appear to have significantly different expression between symbiotic and aposymbiotic anemones, including ≥85 for which there are changes in expression of five-fold or more (Table 6). These findings show the value of a comprehensive analysis of differential expression, because previous studies using microarrays, EST counts, and measurements of protein abundance had found much smaller numbers of differentially expressed genes (Weis and Levine 1996; Kuo *et al.* 2004, 2010; Barneah *et al.* 2006; Rodriguez-Lanetty *et al.* 2006; Ganot *et al.* 2011; Yuyama *et al.* 2011). We have focused our more detailed analyses to date on several groups of genes whose differential expression suggests interesting hypotheses about the biology of the symbiosis.

### Genes controlling metabolism and transport in gastrodermal and epidermal cells

Given the intimate relationship between the symbiotic partners, we were not surprised to observe substantial changes in the expression of many genes encoding proteins involved in small-molecule metabolism and transport. We present here some speculative but testable hypotheses about how these changes might reflect the establishment and maintenance of the symbiosis.

#### Glucose transport within gastrodermal cells:

**Figure 7:**
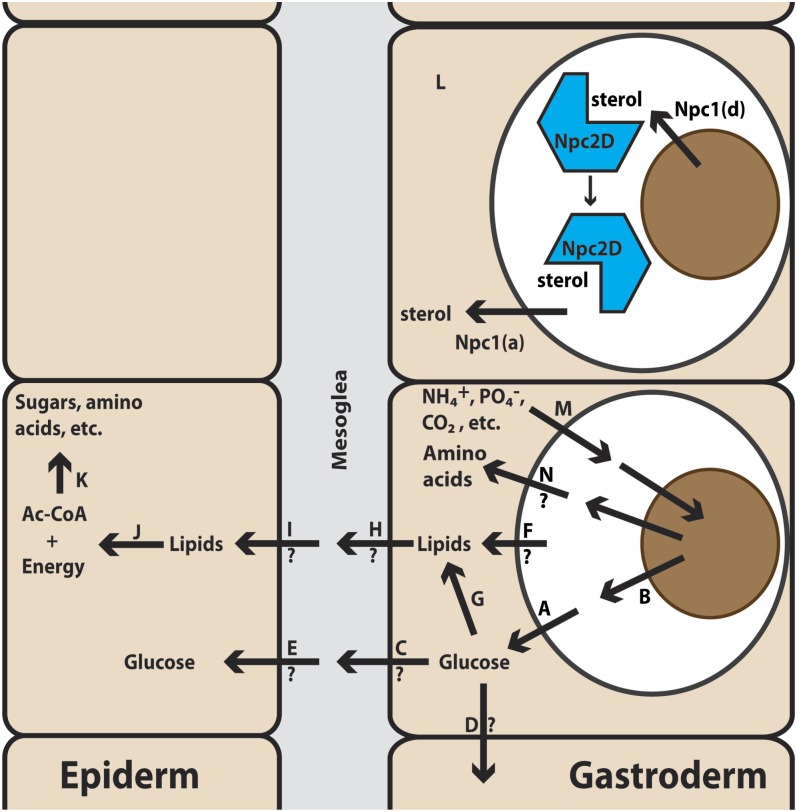
Summary of hypotheses about metabolism and metabolite transport as suggested by the gene expression data and previously available information. Letters “A” through “N” are for reference in the text. Thick arrows across membranes indicate transporters hypothesized to be present in those membranes; thick arrows within cells, metabolic pathways hypothesized to be important in those cells; thin arrow, presumed diffusion of Npc2-sterol complexes; Npc1(a), the anemone-derived Npc1-like protein described in the text; Npc1(d), a presumed but as-yet-unidentified sterol transporter produced by the dinoflagellate and present in its plasma membrane; ?, hypotheses that we consider to be more problematic. Not shown because of their potential complexity are the other changes in inorganic-nutrient transport (*e.g.*, a damping of NH_4_^+^ and CO_2_ excretion across the apical plasma membranes of the gastrodermal cells) that are likely to occur upon the onset of symbiosis. All of these hypotheses should be testable through a combination of experiments including protein localization by immunofluorescence and/or cell fractionation, studies of separated gastrodermal and epidermal cell layers, sterol-binding experiments on Npc2 proteins, and others. See text for additional details.

Because glucose appears to be the major form in which fixed carbon is transferred from the dinoflagellate to the host (Whitehead and Douglas 2003; Burriesci *et al.* 2012), it was not surprising to find the transcripts for three presumed glucose transporters among those highly upregulated in symbiotic animals (Table 7, lines 1–3). Because mammalian GLUT8 is localized to the endosome membrane (Augustin *et al.* 2005), it is likely that one or both of the *Aiptasia* GLUT8 orthologs localize to the symbiosome membrane (Figure 7A), a hypothesis that should be readily testable by immunolocalization studies once appropriate antibodies are available. The putative Na^+^-glucose/*myo*-inositol co-transporter might also be involved in glucose transport across the symbiosome membrane. It should be noted that there must also be a *Symbiodinium* protein that transports large amounts of glucose into the symbiosome lumen (Figure 7B); it should be possible to identify the corresponding gene(s) among those expressed differentially in *Symbiodinium* cells growing *in hospite* relative to those growing in culture.

#### Possible glucose transport between cells:

To our knowledge, there is currently no information regarding whether and how the gastrodermal cells provide energy to the epidermal cells, cells in the mesoglea, and gastrodermal cells that lack dinoflagellates and/or access to nutrients from the gastric cavity, and regarding whether these modes of nourishment change on establishment of symbiosis. Nourishment of the epidermal cells is a major issue because these cells neither contain dinoflagellates nor have direct access to food, but they presumably require large amounts of energy for maintenance, reproduction, nematocyst replacement, and mucus production [which is extensive and has been reported to consume as much as 40% of the energy available to corals (Crossland *et al.* 1980)]. Thus, one or more of the upregulated glucose transporters might be found in the basolateral membranes of the gastrodermal cells (Figure 7, C and D), the basolateral membranes of the epidermal cells (Figure 7E), or both. These questions should be resolvable by immunolocalization experiments and/or experiments in which gene expression is evaluated in separated tissue layers (Ganot *et al.* 2011).

#### Fatty-acid metabolism and transport:

Our previous study of the transfer of fixed carbon from the dinoflagellates to the host was more comprehensive in its evaluation of polar than of nonpolar compounds (Burriesci *et al.* 2012). Thus, it is conceivable that the upregulation of genes encoding proteins of fatty-acid metabolism and transport reflects a significant role of fatty acids in this transfer (Figure 7F). Importantly, however, (1) we saw upregulation of genes of both fatty-acid synthesis and fatty-acid breakdown (suggesting that gastrodermal and epidermal cells may be behaving differently) and (2) in most animals, high levels of glucose (such as those expected in the cytoplasm of gastrodermal cells harboring dinoflagellates) inhibit β-oxidation and stimulate fatty-acid synthesis (Randle 1998). Thus, we think it more likely that gastrodermal cells synthesize fatty acids from the glucose provided by the dinoflagellates during the day (Figure 7G), store them in neutral fats or wax esters (Crossland *et al.* 1980; Harland *et al.* 1993), and subsequently mobilize them to serve as an energy supply at night and/or for transfer into the mesoglea (Figure 7H) and thence into the epidermal cells (Figure 7I). The epidermal cells would metabolize the fatty acids by β-oxidation (Figure 7J) to provide energy and acetyl-CoA building blocks. The apparent upregulation of the glyoxylate cycle (Figure 7K) (see *Results*) would be explained by the need of the epidermal cells to synthesize carbohydrates, amino acids, and other compounds from acetyl-CoA. Testing of these hypotheses should be possible by immunolocalization of the relevant proteins, *in situ* mRNA hybridization, and/or examination of gene expression in separated gastrodermal and epidermal tissue layers.

#### Transport of sterols as building blocks and/or symbiosis signals:

A previous study showed that the anemone *A. viridis* has at least two genes encoding Npc2-like proteins, one of which is in a subfamily distinct from that of the mammalian and *Drosophila* Npc2 proteins and was upregulated in symbiotic gastrodermal tissue (Ganot *et al.* 2011). We have confirmed and extended these findings by showing that *Aiptasia* and several other cnidarians also contain multiple genes encoding Npc2-like proteins. In a phylogenetic analysis, one of the *Aiptasia* proteins clustered with the mammalian and *Drosophila* proteins and shares with them key residues implicated in cholesterol binding. Four other *Aiptasia* proteins, including the one whose transcript is massively upregulated in symbiotic anemones (Npc2D), belong to a separate subfamily that also contains the upregulated *A. viridis* Npc2D; members of this subfamily do not share the residues implicated in cholesterol binding (Figure 2B). In contrast, we identified only one *Aiptasia* gene encoding an unequivocal Npc1-like protein; its expression did not change between aposymbiotic and symbiotic animals. These observations suggest the following speculative model (Figure 7L). A *Symbiodinium*-encoded sterol transporter (*e.g.*, an Npc1-like protein) is present in the dinoflagellate plasma membrane and passes one or more dinoflagellate-synthesized sterols to the *Aiptasia*-encoded Npc2D, which is localized specifically to the symbiosome lumen. Npc2D in turn passes the sterol(s) to the *Aiptasia*-encoded Npc1 in the symbiosome membrane, which passes them to a sterol-carrier protein in the cytosol. If the same Npc1 protein functions in different membranes, in concert with all of the different Npc2 partners, and in other cell types as well as gastrodermal cells containing dinoflagellates, then this could explain why its transcript is not significantly upregulated upon the onset of symbiosis.

The model to this point is agnostic about what sterols might be transferred, and to what end(s), but these are also important questions. The membranes of *Aiptasia*, like those of other animals, presumably contain cholesterol as an essential component. This cholesterol could be obtained from food, by *de novo* synthesis, or by modification of one or more of the distinctive, non-cholesterol sterols (dinosterol, gorgosterols) produced in large quantities by many dinoflagellates (but not by other marine algae that have been investigated) (Giner and Wikfors 2011). Based on its sequence (Figure 2B), *Aiptasia* Npc2A seems the most likely to be involved in cholesterol traffic *per se*, whereas Npc2D (and Npc2B, Npc2C, and Npc2E: Figure 2A) might all be involved in traffic of various other dinoflagellate-produced sterols. The latter might serve only as precursors of cholesterol, but a more intriguing possibility is that one or more of these molecules serves as the signal that a symbiosis-compatible dinoflagellate is present in the phagosome/symbiosome.

Although the model of Figure 7L is speculative, it is important to note that its major features should be testable by experiments that include (1) localization to the gastrodermal and/or epidermal cell layers of the expression of the several genes, (2) protein localization by immunofluorescence and/or cell fractionation, (3) using purified, labeled sterols to test the binding specificities of bacterially expressed Npc2 proteins (Ko *et al.* 2003), and (4) tests of the abilities of exogenously added Npc2 proteins to complement the loss-of-cholesterol-transport phenotype in *NPC2*-knockout human cells (Ko *et al.* 2003). It is also worth noting that if this model is correct, Npc2D proteins would become an invaluable marker for the isolation of intact (*i.e.*, nonruptured) symbiosomes.

#### Transport and metabolism of inorganic nutrients and the coordination of nitrogen and carbon metabolism:

It is clear that symbiotic cnidarians must transport CO_2_, NH_4_^+^, and other inorganic nutrients across the symbiosome membrane—and indeed concentrate at least some of these materials within the symbiosome lumen—to provide their resident dinoflagellates with these essential building blocks (Pernice *et al.* 2012; Bertucci *et al.* 2013). Thus, at least some of the many inorganic-nutrient transporters and transport-related proteins that are upregulated in symbiotic anemones (see *Results*) are presumably localized to the symbiosome membrane or lumen (Figure 7M). However, it seems virtually certain that the onset of symbiosis also induces changes in inorganic-nutrient transport across the various plasma-membrane domains. For example, aposymbiotic anemones presumably excrete CO_2_ and NH_4_^+^ across the apical membranes of both epidermal and gastrodermal cells, whereas symbiotic anemones presumably reduce such excretion at least from the gastrodermal cells, and may even achieve a net uptake of both compounds from the environment. Although the possibilities are too numerous and too complicated for ready depiction in Figure 7, determining the cellular (gastrodermal, epidermal, or both) and intracellular (symbiosome membrane, apical plasma membrane, basolateral plasma membrane, and/or other) localizations of the differentially regulated transporters and transport-related proteins should begin to answer many of these questions with a clarity that has not been possible before.

The changes in NH_4_^+^ movement upon symbiosis establishment also bear on the probable linkage between carbon and nitrogen metabolism. In this regard, two non-mutually exclusive models have been put forward. The “nitrogen-recycling model” focuses on the possibility that continued host catabolism of amino acids produces NH_4_^+^ that is supplied to the dinoflagellate, which in turn releases amino acids for use by the host (Figure 7N) (Cates and McLaughlin 1976; Szmant-Froelich and Pilson 1977; Wang and Douglas 1998). In contrast, the “nitrogen-conservation model” focuses on the possibility that the fixed carbon provided by the dinoflagellate leads to a suppression of host amino-acid catabolism and therefore of the generation of NH_4_^+^ to be used by the dinoflagellate or excreted (Rees 1986; Rees and Ellard 1989; Wang and Douglas 1998). Our data provide support for aspects of both models. In particular, we observed upregulation of the genes of the GS-GOGAT cycle (the first dedicated step of NH_4_^+^ assimilation in animals) in symbiotic anemones, indicating that the release of metabolites from the dinoflagellate promoted synthesis, rather than catabolism, of some amino acids. (*The simultaneous upregulation of a presumably catabolic glutamate dehydrogenase might be taken as countervailing evidence, but this observation is difficult to interpret without knowing in which cell type(s) and cytoplasmic compartment(s) this upregulation occurs.) Meanwhile, we also obtained strong support for previous evidence that cnidarians, like other animals, can only synthesize eight of the 20 amino acids found in proteins from intermediates of the central metabolic pathways. The remaining 12 amino acids must thus be obtained either from food or from the dinoflagellates, and our observation that the host genes for at least nine amino-acid transporters are upregulated in symbiotic anemones (see *Results*) suggests strongly that the dinoflagellates indeed contribute to the host'samino acid supply. It should be informative to determine the localizations and amino-acid specificities of these host-encoded transporters as well as to identify any amino-acid transporters expressed differentially by *Symbiodinium* during growth *in hospite* (because it is unlikely that free-living dinoflagellates would export amino acids into the surrounding sea water).

### Recognition and tolerance of dinoflagellate symbionts by the host

Establishment and maintenance of the mutualistic relationship also require that the host recognize and tolerate the dinoflagellate symbionts. Thus, it was not surprising that an unbiased screen for functional groups that were enriched among the differentially expressed genes revealed three groups that might be involved in these processes, as discussed below.

#### Response to oxidative stress:

Both *a priori* logic and considerable experimental evidence support the view that possession of an intracellular photosynthetic symbiont imposes oxidative stress on the host, particularly under conditions in which chloroplast damage may result in enhanced production of reactive oxygen species (ROS) (D’Aoust *et al.* 1976; Lesser 1997, 2006; Venn *et al.* 2008). If not detoxified, ROS can damage DNA, proteins, and lipids (Lesser 2006), and it is widely believed that ROS production under stress is the major trigger of symbiosis breakdown during bleaching (Lesser 1997, 2006, 2011; Jones *et al.* 1998; Downs *et al.* 2002; Venn *et al.* 2008; Weis 2008). Surprisingly, however, our results provide no support for these views. Although a catalase gene is expressed at high levels in symbiotic anemones (Sunagawa *et al.* 2009, and see the RNA-Seq read counts provided in File S2 and File S3), as it is in other animals (Kodzius *et al.* 2004), both it and most of the other differentially regulated genes in this GO category were actually downregulated in symbiotic animals, and the two that were upregulated do not seem likely to be involved in ROS detoxification (see *Results*). Previous studies of other species of anemones have also found host genes thought to be involved in ROS detoxification (copper/zinc superoxide dismutase and glutathione S-transferase) to be downregulated in symbiotic relative to aposymbiotic individuals (Rodriguez-Lanetty *et al.* 2006; Ganot *et al.* 2011). Although other studies have indicated that symbiotic cnidarians have higher superoxide-dismutase activities than their aposymbiotic counterparts (Dykens and Shick 1982; Furla *et al.* 2005), it was not determined whether the enzyme was of host or dinoflagellate origin. Thus, it is possible that the hosts are protected from ROS by symbiont-generated antioxidants and can reduce the expression of their own enzymes (Rodriguez-Lanetty *et al.* 2006). These other studies, like ours, were conducted under conditions thought to be nonstressful, and it is possible that a different picture would emerge under stressful conditions. In that regard, however, we have also recently observed that bleaching under heat stress can occur rapidly in the dark, when photosynthetically produced ROS cannot be present (Tolleter *et al.* 2013).

#### Inflammation, tissue remodeling, and response to wounding:

Inflammation is a protective tissue response to injury or pathogens that serve to destroy, dilute, and/or wall off both the injurious agent and the injured tissue (Sparks 1985). In invertebrates, including anthozoans, the inflammation-like response involves both cellular and humoral aspects, including the infiltration of immune cells such as amoebocytes and granular cells (Patterson and Landolt 1979; Mydlarz *et al.* 2008; Palmer *et al.* 2008), phagocytosis and/or encapsulation of foreign material (Patterson and Landolt 1979; Olano and Bigger 2000; Petes *et al.* 2003; Mydlarz *et al.* 2008), and the production of cytotoxic molecules such as ROS, nitric oxide, lysozyme, antimicrobial peptides, and intermediates of the phenoloxidase cascade (Hutton and Smith 1996; Perez and Weis 2006; Mydlarz *et al.* 2008; Palmer *et al.* 2008). Our data suggest that the establishment of symbiosis is associated with an overall attenuation of the inflammatory response (see *Results*), presumably to allow the dinoflagellate to co-exist peacefully with the host rather than being attacked as a harmful invader. Other studies also support this conclusion and suggest that it may be a general feature of the means by which animal hosts accommodate symbiotic microbes. For example, when symbiotic and aposymbiotic *Aiptasia* were challenged with bacterial lipopolysaccharide (LPS), the former produced much less nitric oxide than did the latter (Detournay *et al.* 2012), and in two hard-coral species with inflammatory-like responses, dinoflagellate densities were lower in the “inflamed” than in the adjacent healthy tissues (Palmer *et al.* 2008). Similarly, successful colonization of squid light organs by symbiotic bacteria is associated with an irreversible attenuation of host nitric-oxide production (Davidson *et al.* 2004; Altura *et al.* 2011).

Of particular interest because of its massive upregulation in symbiotic anemones is the gene encoding scavenger receptor B class member 1 (SRB1); upregulation of SRB1 was also observed previously in symbiotic individuals of the anemone *Anthopleura* (Rodriguez-Lanetty *et al.* 2006). SRB1 is a member of the CD36 protein family and is a transmembrane cell-surface glycoprotein that has been implicated in multiple functions, including lipid transport (Figure 2, Table 7, and associated text), cell adhesion, wound healing, apoptosis, and innate immunity (Areschoug and Gordon 2009). Perhaps of most interest is its role in *Plasmodium* infection, because the apicomplexan parasites are a sister taxon to the dinoflagellates (Baldauf 2003). SRB1 has been shown to boost host hepatocyte permissiveness to *Plasmodium* infection, promote parasite development by acting as major lipid provider, and enable adhesion between *Plasmodium*-infected and uninfected erythrocytes, thus allowing for movement of parasites between host cells (Adams *et al.* 2005; Rodrigues *et al.* 2008; Yalaoui *et al.* 2008). It is possible that SRB1 has similar functions in the cnidarian–dinoflagellate symbiosis, and further investigation of its function by protein localization and knockdown of function should be highly informative.

#### Apoptosis and cell death:

The possible roles of apoptosis and necrotic cell death in the breakdown of symbiosis under stress have been investigated (Dunn *et al.* 2002, 2004, 2007; Richier *et al.* 2006; Ainsworth *et al.* 2011; Kvitt *et al.* 2011; Pernice *et al.* 2011; Tchernov *et al.* 2011), and a role for apoptosis in the post-phagocytic selection of compatible symbionts has also been suggested (Dunn and Weis 2009). However, the possible role of apoptosis in maintenance of a stable symbiotic relationship has not been addressed experimentally. Others have suggested that apoptosis might contribute to the dynamic equilibrium between host and symbiont cell growth and proliferation that is presumably necessary to ensure a stable relationship (Muscatine and Pool 1979; Fitt 2000; Davy *et al.* 2012), and our observation that 13 apoptosis/cell death–related genes were differentially expressed (some massively so) in symbiotic relative to aposymbiotic anemones is broadly consistent with this possibility. However, the complexity of the apoptotic pathways and the fact that a single protein can have either pro-apoptotic or anti-apoptotic function depending on its localization and/or the presence or absence of other specific signals makes it impossible to draw firm conclusions from gene expression data alone.

Nonetheless, it is worth noting the potential role of tumor-necrosis factor (TNF) family members and their associated proteins, which are prominent regulators of cell survival, proliferation, and differentiation in both vertebrates and invertebrates (Neumann *et al.* 2013). We found a TNF-family ligand, a TNF receptor, a receptor-associated factor, and the functionally related “growth-arrest and DNA-damage–inducible protein” all to be upregulated in symbiotic anemones (1.9-fold, 60-fold, 1.8-fold, and 5.1-fold, respectively). These proteins are capable of inducing caspase-dependent apoptosis by at least two different pathways (Zhang *et al.* 1999; Sinha and Chaudhary 2004; Burkly *et al.* 2007; Sabour Alaoui *et al.* 2012), as well as of activating the multifunctional NFκB and MAPK pathways (Sinha and Chaudhary 2004; Burkly *et al.* 2007), so that they may coordinate multiple biological processes to regulate symbiotic stability. Interestingly, genes encoding TNF receptors and receptor-associated proteins were also prominent among the genes found to be upregulated in corals living under chronic mild heat stress (Barshis *et al.* 2013).

## Supplementary Material

Supporting Information
