# Supplementary material for: Extensive Differences in Gene Expression Between Symbiotic and Aposymbiotic Cnidarians
Source: G3 (Bethesda). 2013 Dec 24;4(2):277–95. doi: 10.1534/g3.113.009084 (PMC3931562; doi:10.1534/g3.113.009084)
Supplement: Supporting Information [file supp_4_2_277__index.html]

Extensive Differences in Gene Expression Between Symbiotic and Aposymbiotic Cnidarians — Supporting Information 

# Extensive Differences in Gene Expression Between Symbiotic and Aposymbiotic Cnidarians

## Supporting Information for Lehnert *et al.*, 2013

**Files in this Data Supplement:**

- Supporting Information - Figures S1 and S2, Tables S1-S6, and Files S1-S5 (PDF, 2.1 MB)
- Figure S1 - Alignments of Npc2 sequences from *Aiptasia* and other organisms (PDF, 1.4 MB)
- Figure S2 - Distinct but related genes whose products may be involved in host tolerance of the symbiont (PDF, 553 KB)
- Table S1 - Correlation between RNA-Seq and RT-qPCR measurements of differential gene expression in symbiotic relative to aposymbiotic anemones (PDF, 351 KB)
- Table S2 - Primer sequences and product sizes for RT-qPCR data (PDF, 333 KB)
- Table S3 - Transport-related genes showing differential expression in symbiotic relative to aposymbiotic anemones (PDF, 388 KB)
- Table S4 - Lipid-metabolism genes showing differential expression in symbiotic relative to aposymbiotic anemones (PDF, 359 KB)
- Table S5 - Presence or absence in the *Aiptasia* transcriptome of genes encoding the enzymes involved in the synthesis of particular amino acids (PDF, 485 KB)
- Table S6 - Genes potentially involved in host tolerance of the symbiont that are differentially expressed between symbiotic and aposymbiotic anemones (PDF, 463 KB)
- Supporting References - (PDF, 346 KB)
- File S1 - Supporting Materials and Methods (PDF, 487 KB)
- File S2 - Expression Data Experiment 1 (.xlsx, 730 KB)
- File S3 - Expression Data Shared (.xlsx, 346 KB)
- File S4 - Transcriptome data (.zip, 29.4 MB)
- File S5 - Predicted species of origin of each contig (.zip, 914 KB)
