# Supplementary material for: Extensive Differences in Gene Expression Between Symbiotic and Aposymbiotic Cnidarians
Source: G3 (Bethesda). 2013 Dec 24;4(2):277–95. doi: 10.1534/g3.113.009084 (PMC3931562; doi:10.1534/g3.113.009084)
Supplement: Supporting Information [file supp_g3.113.009084_009084SI.pdf]

G3 Genes | Genomes | Genetics

Extensive Differences in Gene Expression between Symbiotic and Aposymbiotic Cnidarians

Erik M. Lehnert[*,#], Morgan E. Mouchka[§,#], Matthew S. Burriesci[*], Natalya D. Gallo[*], Jodi A. Schwarz[†], John R. Pringle[*]

[*]Department of Genetics, Stanford University School of Medicine, Stanford, CA 94305
[§]Department of Ecology and Evolutionary Biology, Cornell University, Ithaca, NY 14850
[†]Biology Department, Vassar College, Poughkeepsie, NY 12604
# Equal contributions.

Corresponding author:
John R. Pringle
Department of Genetics, MC-5120
300 Pasteur Drive
M-322 Alway Building
Stanford University School of Medicine
Stanford, CA 94305
Telephone: (650) 723-8523
Email: jpringle@stanford.edu

DOI: 10.1534/g3.113.009084

A Full-length alignments of *Aiptasia* and other Npc2 proteins

```
Aiptasia Npc2D       MKPAISLALVVIIAAVTSIQAMKLKFKDCGSQVGE--IVSLDVTPCTSDPCSLKRGGTNA
Anemonia Npc2D       MKFLVLLLCLQIIWSLS--EARKLSFKDCGSKVGK--LVSFDLSPCSQDPCIIKR-GSNA
Human Npc2           MRFLAATFLLLALSTAA--QAEPVQFKDCGSVDGV--IKEVNVSPCPTQPCQLSK-GQSY
Drosophila Npc2A     MLRYAVIACAALVVF-----AGALEFSDCGSKTGKFTRVAIEGCDTTKAECILKR-NTTV
Aiptasia Npc2A       ---------------------------------------------------MKRK-ELKS
Anemonia Npc2A       MAKFFLIACMLYVLSLA--GAEVVDFDDCSGGKGKGEIEKLEIIPCPTQPCQLKK-GSKV

Aiptasia Npc2D       TVTINFKPHEQVTQSKIYVYAIIGIIPIPLPIPNPDACTGHGLTCPLASGKDVELVVKQS
Anemonia Npc2D       TGTVTFIPSEEVTSSKVYMYAIIGFIPVPLPLPNTDGCKGYGLTCPLKSGKPDELVFSHS
Human Npc2           SVNVTFTSNIQSKSSKAVVHGILMGVPVPFPIPEPDGCKS-GINCPIQKDKTYSYLNKLP
Drosophila Npc2A     SFSIDFALAEEATAVKTVVHGKVLGIEMPFPLANPDACVDSGLKCPLEKDESYRYTATLP
Aiptasia Npc2A       SVKRTFIPHENVTDAESSVHGKVMGFWVPFPLPNAHACKDSGVKCPLVAGSKYEYSSTLD
Anemonia Npc2A       QIKVTFVPHEDLTEATSVVHGEIGGFPVPFPLPNSNCCKDSGLTCPLKAGQKYVYTSALD

Aiptasia Npc2D       IDSTFPAGKVTVKAELKDQVQNNVLCGEVTLTLM---
Anemonia Npc2D       IDSTFPAGTVTLKGELKDQEENNIFCGKISLTLQ---
Human Npc2           VKSEYPSIKLVVEWQLQDDKNQSLFCWEIPVQIVSHL
Drosophila Npc2A     VLRSYPKVSVLVKWELQDQDGADIICVEIPAKIQ---
Aiptasia Npc2A       IKSAYPAISVVVKWQLQDGKGQDLYCFEVSAKIVS--
Anemonia Npc2A       VKSEYPAIKVVVKWEMQDKDNNDVFCFKVATQIVS--
```

B Multiple-sequence alignment used to generate Npc2 phylogenetic tree

```
A_digitifera_Npc2E            -KNCT--KNDDVTVESLDIN---PCSE-EP---CIFHK-GSTVSVTVAF-TPLEEVKSGE
A_digitifera_Npc2F            -KNCA--SRKYALPLKVAIN---PCTK-QP---CTLHP-GKKASIAVVV-KPLVTIRRGT
O_carmela_Npc2a               -SNCTSNPGPSTLGKTVNVTAVPPCDT-AP---CVVHQ-GESLNVTVTF-VPNVAIENFT
A_digitifera_Npc2D            -QTC---DKPSGRLNSVDVT---PCNG-NP---CVFKR-GTNETITVTF-TPNEVVSKGK
Aiptasia_Npc2E                -KDC---GSKGATIVRLDIS---PCEE-EP---CNFKT-GTTVTGTLTF-VAKEYFTSGR
N_vectensis_Npc2B             -RDC---GSQEGEIVGMDIS---PCDS-EP---CVLKR-GTSVDGSLTF-IPHEDLKRAK
Aiptasia_Npc2B                VVVL---VVVVGIVVVVDVD---QCTSDDP---CSLKR-GTNVTSTATM-IPLEEVTQAT
Aiptasia_Npc2C                -TDC---GSYLGEIHSLEVN---PCTS-DP---CVLKR-GDNMTSVISF-TPHEQVSAAK
Aiptasia_Npc2D                -KDC---GSQVGEIVSLDVT---PCTS-DP---CSLKRGGTNATVTINF-KPHEQVTQSK
N_vectensis_Npc2C             -QDC---GSKKGELISVDLT---PCSS-DP---CVIKR-GANASGVITF-IPHEVVTSSK
A_viridis_Npc2D               -KDC---GSKVGKLVSFDLS---PCSQ-DP---CIIKR-GSNATGTVTF-IPSEEVTSSK
M_faveolata_Npc2B             -ANCSDVTALEGKLISVDLT---PCPS-QP---CVFHK-GTNVTATIKF-SPEEMVTDGT
D_melanogaster_Npc2A          -SDC---GSKTGKFTRVAIE---GCDT-TK-AECILKR-NTTVSFSIDF-ALAEEATAVK
M_faveolata_Npc2A             -ADC---GSL-AKINFVDVS---PCVM-EP---CELKK-GTNESIEIQF-IPNSNITEGK
A_digitifera_Npc2B            -RDC---GNKELSPAQVIIT---PCPA-EP---CQLKK-GVNESVEVIF-KPTEVVTSAK
A_digitifera_Npc2A            -SYI---GSKESSISQVIVT---PCPA-EP---CQLKK-GVNESIEVIF-KPGEVVTSSK
Human_Npc2a                   -KDC---GSVDGVIKEVNVS---PCPT-QP---CQLSK-GQSYSVNVTF-TSNIQSKSSK
Mouse_Npc2A                   -KDC---GSKVGVIKEVNVS---PCPT-DP---CQLHK-GQSYSVNITF-TSGTQSQNST
Aiptasia_Npc2A                ------------------------------------K-ELKSSVKRTF-IPHENVTDAE
N_vectensis_Npc2A             -KDCSG-GKGEGEIVELDIS---PCPT-QP---CTLHK-GTTVSVNITF-VPHVTLDSGK
A_viridis_Npc2A               -DDCSG-GKGKGEIEKLEII---PCPT-QP---CQLKK-GSKVQIKVTF-VPHEDLTEAT
H_magnipapillata_Npc2D        -QNC---GHLDSNTI-VSIT---PCEK-EP---CTLVR-GSNATLEIQF-KAKHFSKQLK
H_magnipapillata_Npc2B        -KPC----DMSSTVGDVAIS---PCDK-QP---CAFQR-GGSANIEISF-TAAKDADKLT
H_magnipapillata_Npc2A        -KKCTS-PASSAVIGDVIIT---PCDS-LP---CSFKR-GGSGNIKINF-QATKNNSELT
H_magnipapillata_Npc2C        -KKCSS-PASSAVVGDVVIS---PCDN-QP---CQFIR-GGNANIQIHF-QAKKDNSNIT

A_digitifera_Npc2E            LSVDAI-AFGHRLP-M--VRKE--NICEG--HGVT-----CPLEKGKKQTFTINQKVERY
A_digitifera_Npc2F            LELYGIHWLGIKFP-LS-VPNP--DICHG--YGTR-----CPMIANSRVVLSISQTLPSF
O_carmela_Npc2A               VVVHAS-VGIIHVP-YP-VTDP--NGCDTAVTGVT-----CPLKANVAVEWHHSFSVPSI
A_digitifera_Npc2D            ILLYAK-LVLGWIE-LS-LRNP--NICEG--YGLK-----CPLAKGVREELSVTERVPQV
Aiptasia_Npc2E                VKAYAV-IEGVDLP-LP-IPT---DACQG--YGLT-----CPINNGQTANFVIKQEIQAD
N_vectensis_Npc2B             LSAHAI-IDKLPLP-LP-IPS---DACQG--YGLS-----CPVDSGVKSMFKIHQAIESE
Aiptasia_Npc2B                IYMHAT-VSGITIP-ID-IPNP--NACSG--HGLS-----CPLKSGETVELSMVLEVEAK
Aiptasia_Npc2C                IDINAI-IAGSPIH-VH-IPNP--NACDG--HGLK-----CPLEKGKKVELVVSQVIRRS
Aiptasia_Npc2D                IYVYAI-IGIIPIP-LP-IPNP--DACTG--HGLT-----CPLASGKDVELVVKQSIDST
N_vectensis_Npc2C             VLAYAI-FGLIPVP-LP-LPNS--DGCKG--YGLT-----CPLKSGKQVELVFEHYIDQT
A_viridis_Npc2D               VYMYAI-IGFIPVP-LP-LPNT--DGCKG--YGLT-----CPLKSGKPDELVFSHSIDST
M_faveolata_Npc2B             LQVYGF-IEGIKTP-FP-LEQP--DACKE--HGLE-----CPLKSGVTYSLEITLAIKPA
D_melanogaster_2A             TVVHGK-VLGIEMP-FP-LANP--DACVD--SGLK-----CPLEKDESYRYTATLPVLRS
M_faveolata_Npc2A             TVVYGI-IEGVQVP-FP-VDNP--EVCKE--HGIT-----CPMPAEKTQTFKATLPVKSE
A_digitifera_Npc2B            VVIHGI-IEGVRFP-FP-FPHP--NGCKE--HGLE-----CPLKPNKEYTFKATLPVKRT
A_digitifera_Npc2A            VVVHGI-IAGVPVP-FP-ISQP--NGCED--HGLD-----CPLQPNKEYTFKATLPVKSA
Human_Npc2a                   AVVHGI-LMGVPVP-FP-IPEP--DGCKS---GIN-----CPIQKDKTYSYLNKLPVKSE
Mouse_Npc2A                   ALVHGI-LEGIRVP-FP-IPEP--DGCKS---GIN-----CPIQKDKVYSYLNKLPVKNE
Aiptasia_Npc2A                SSVHGK-VMGFWVP-FP-LPNA--HACKD--SGVK-----CPLVAGSKYEYSSTLDIKSA
N_vectensis_Npc2A             AIVHGV-IAGIPVP-FP-LPNA--DVCKN--SGLK-----CPLEPGTKYVYQSSLEVKTM
A_viridis_Npc2A               SVVHGE-IGGFPVP-FP-LPNS--NCCKD--SGLT-----CPLKAGQKYVYTSALDVKSE
H_magnipapillata_Npc2D        TKVYGK--LLFWVPYYN-FGKE--DSCLD--NGIT-----CPVIEDEEYSYSQSLHISKL
H_magnipapillata_Npc2B        TVVKGK-IGPIWVP-FP-LSQP--DACNN--EGLT-----CPIKSSQKYTYQYSLPISES
H_magnipapillata_Npc2A        SVVKGK-IGPLWVP-FP-LSQP--DACQN--EGIT-----CPIKDGQSYLFSYDLPISTT
H_magnipapillata_Npc2C        TIVKGK-IGPLWVP-FP-LSQP--DGCLN--DGII-----CPVKTDQQYVYSYDLPISKS

A_digitifera_Npc2E            YPPLPI-DVEAYVENDNRK----ILC
A_digitifera_Npc2F            VPMGSY-QLQAVMKDQLGR--M-VLC
O_carmela_Npc2a               APKGPVEIITWELQAPSKE--D-VAC
A_digitifera_Npc2D            LPSSTR-EVKAKLVDQNGG--T-VVC
Aiptasia_Npc2E                FPKVKL-QLKGEVMDPQGN--M-LFC
N_vectensis_Npc2B             FPVGNL-TLKAAVTDSDTS--QVVFC
Aiptasia_Npc2B                FPRGKV-ILKTELKDQAKN--D-IFC
Aiptasia_Npc2C                APPGRY-RIRTELKEQYGI--D-VFC
Aiptasia_Npc2D                FPAGKV-TVKAELKDQVQN--N-VLC
N_vectensis_Npc2C             FPTGHL-TLKAELKDQDSD--V-VIC
A_viridis_Npc2D               FPAGTV-TLKGELKDQEEN--N-IFC
M_faveolata_Npc2B             YPSIQL-VAQMDFKLPDDG--Y-LFC
D_melanogaster_2A             YPKVSV-LVKWELQDQDGA--D-IIC
M_faveolata_Npc2A             YPALQL-DVKWELHDQDAK--V-VYC
A_digitifera_Npc2B            YQDVCM-I---RLL---------CSC
A_digitifera_Npc2A            YPDIKL-VVKWQLLDQNAN--S-VFC
Human_Npc2a                   YPSIKL-VVEWQLQDDKNQ--S-LFC
Mouse_Npc2A                   YPSIKL-VVEWKLEDDKKN--N-LFC
Aiptasia_Npc2A                YPAISV-VVKWQLQDGKGQ--D-LYC
N_vectensis_Npc2A             YPSLKL-VVRWEIQDNKNK--D-VLC
A_viridis_Npc2A               YPAIKV-VVKWEMQDKDNN--D-VFC
H_magnipapillata_Npc2D        NPKISI-PVKWLIQNEAEK--D-LVC
H_magnipapillata_Npc2B        YPKINL-PVSWELKDEKGE--S-LVC
H_magnipapillata_Npc2A        YPAISL-VVSWEIQDENGN--D-VVC
H_magnipapillata_Npc2C        YPAISV-VVSWELQDENGN--D-LVC
```

Figure S1 Alignments of Npc2 sequences from *Aiptasia* and other organisms. (A) Full-length alignment of selected Npc2-like proteins from *Aiptasia* sp. (this study), *A. viridis* (Ganot et al. 2011), human, and *D. melanogaster.* Red and green dots, amino acids that are identical (red) or similar (green: I,V,L; S,T; D,E; K,R; Q,N) between *Aiptasia* NpcD and human Npc2; pink shading,

amino acids whose mutation to alanine ablates the cholesterol-binding function of Npc2 in mammalian cells (Ko *et al.* 2003); blue shading, conserved cysteines used to identify conserved regions of the proteins for phylogenetic analysis; black underline, the conserved region used for phylogenetic analysis. (B, next page) The multiple-sequence alignment of the conserved regions used to produce the phylogenetic tree in Figure 2A.

A **Sequence alignment of *Aiptasia* peroxidasin-related proteins 1 and 2**

```
Apr2            TKTPESPMSSSKDPGKPYSCSHTKFFIVTFLSHTLLLCAGIVPLYITTNHRLVTVEDRLT 60
Apr1            ------------------------------------------------------------

Apr2            VHDMELNSCCLVKEGYGNFQDHEETKVTLNREKYVQTQFIDRVRRNTPYISRDVLNSIRM 120
Apr1            ------------------------------------------------------------

Apr2            EVRNHILNLTASQFCQVPDKICTRGAPGKRGLRGRRGSRGRRGRPGHKGIKGLPGKYGKQ 180
Apr1            ---------------------RQRGPPGPPGLPGKSGPRGSIGPQGPK------------ 27
                           Box 1 -->**.**  ** *: *.**  *  * *

Apr2            GLRGFPGQKGQKGDIGNRGPPGLPGPKGERGKEVTEPSVFISPSILTVTENQTATFHCNA 240
Apr1            ------GLAGKKGDIGRPGLPCKPLIIN------YPPKVSLPVGPIYVKEGDNLILSCHV 75
                      *  *:*****. * *  *    .Box 2 -->*.* :. . : *.*.:.  : *:.

Apr2            HGYPKPQITWKMGSGKQIDFGKTRIDKSAGLLEISNVSEKDTGNYTCSAKSVLGEDANTV 300
Apr1            TGYPKPKVTWSKVMDS---LPSKRSFITTNRLKVLSVQKQDSGLYVCAGSNTLGSAVETI 132
                 *****::**.   ..   :  ..*   ::. *:: .*.::*:* *.*:....**. .:*:

Apr2            SLLVKFPPRFTEVQKPFQTILQGSTVNLKCAALGYPPPIITWTKMLGSLPTKRSQQNGGK 360
Apr1            KVIVVSAPKFISTPPQQVNKNTCEKLTLDCQARGDPPAVITWSKEKGRLPTDRTQLINGR 192
                .::*  .*:* ..     .    ..:.*.* * * **.:***:*  * ***.*:*  .*:

Apr2            LTITRFQQSDSGSYQCEAVNSVGKNIFYTTLSFGACDDALGMQSKAIRDSQITASSSYSS 420
Apr1            LTITGMTTSDAGKYTCTAVSAGVATSKSVTRVTVKETGKLKFSRDSVTDYIVVKR----- 247
                **** :  **:*.* * **.:   .   .*       . * :. .:: *  :.
                                                  <-- Box 3

Apr2            AYLPYYGRLNIVLGYGGWLAKSNTKGQWIQVDLLQATRITAIATQGTSKYDEWTTSYSLQ 480
Apr1            -KLPAMARLTVCL----WMMTSKKNSVLISYAVPGSINEILLLDVG-KRLSVWLGDVSWD 301
                  **  .**.: *    *: .*:.:.  *.  :  : .   :   * .: . *  . * :

Apr2            YSYDGTSFRDYEGGKTLPGNSDRSTVVKNNLDPAIAARYIRLLPKTYHSYMVIRMELYGC 540
Apr1            SGVHVTDGQWHHICATWDNSAGQTILYKDGVRRAPSSTRSR------------------- 342
                 . . *. : :.   *  ..:.:: : *:.:  * ::   *

Apr2            QL 542
Apr1            --
```

B **Comparison of protein domains found in human peroxidasins and the *Aiptasia* peroxidasin-related proteins**

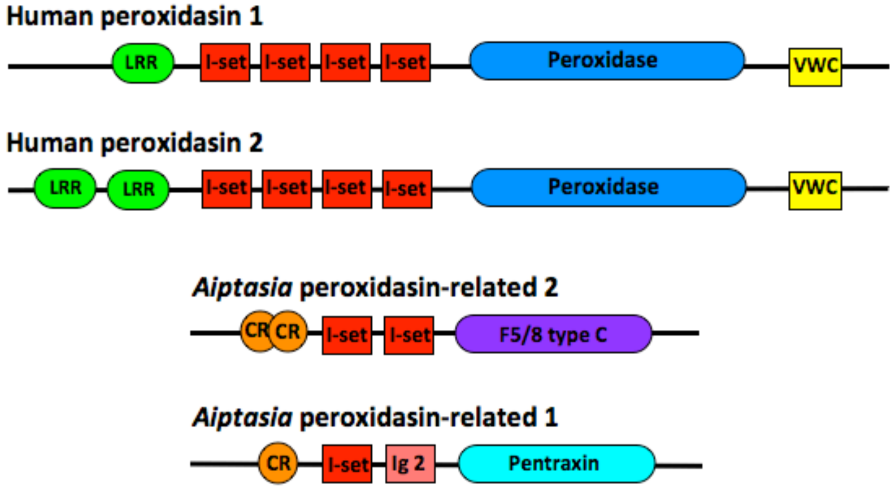

C **Sequence alignment of *Aiptasia* plasma-kallikrein homologs**

```
73922       --------------------------------------------LRSTPTIGIDIKALHF 16
84752       -------------------------------------------------LTG------DV 5
12789       TGDCSPGGSGYYMYAEASSPRRDMDVARLETSQKFSFDGGNKCLKFMYHMYGKDMGFMDV 120
                                                               *      ..

73922       VSNIRLEATTKKLKMTLG------------------------SLFIILTVASCVILRSSA 52
84752       VVS--VVVWTAGVSIG------------------------------SSFDVSCVSFASVS 33
12789       YVGNDLVWWRGGGSMGNTWHSANIPVLISSGSYKIAFEAYRGYGYRSDIAIDEISLEDCT 180
              .  :       .:                                    . : : . :

73922       EES--------------------------------------VNATLGDIEDKVDMIETRV 74
84752       LVG---------------------------------------HVVEGSCGYKP---NVRI 51
12789       TATPQPPTQPPATQPPPPPSTQAPTPPPPTQAPTPPPPNTPPPPPSGSCGIRP---LTRI 237
                                                          *.   :     .*:

73922       IKGQNANLHEWPWQVSLEYNG---HHICGGSLVKRDWVLTAAHCFNRFRYVPSWRVVVGR 131
84752       VGGTEAPPGAWPWQVMIRTKSSFPGHFCGGSLIHPQWVVTAAHCLYGRTEN-DFFVRLGA 110
12789       VGGTDANHGDWPWQALLRYAS--GSQFCGGALVAPQWVVSASHCVSSLSAS-DIHIRMGA 294
            : * :*    ****. :.  .    ::***:*:  :**::*:**.       .  : :*

73922       HTIKGVTQFERRYQVRRIITHAYYHKTR-FINDVALMQLQASVVLSKQVNVITLPTHNNR 190
84752       HYRDSVMGTEQDIDIEKMIKHWSYGKPKSLSHDIALLKLKRPVRLSKDAGLVCLPRTYSN 170
12789       HKRTSSVGTEQDFKVIKKIMHESYQNPKQYSNDIALLKLEKPVTLDKYTNLVCLPPRAVD 354
            *   .    *:  .: : * *  * :.:   :*:**::*: .* *.* ..:: **

73922       VAAGSVCYITGWGLTHGLIKESAAHVLQEAPLTIAAFWHCSAVNNQLNIEIDEKTMVCAG 250
84752       VLQGTKCWVTGWGKLS--AGGADPRVLMQVSVPIVSRRKCQNS----YPGQIDDSMICAG 224
12789       IATDSKCWITGWGTLS--SGGSQPETLQQAEVPIVSPATCQNS----YPNMIDKTMVCAG 408
            :  .: *::****        : ...* :. :.*.:   *.           :.:*:***

73922       G--GGKGGCRGDSGGPLACNERGHWILRGVVSWGHAKCSAEEFYTVFARVSSFVDWIKKI 308
84752       YDQGGRDSCQNDSGGPLVCEQFGRFYLEGVVSWGEG-CAGRLKYGVYANVRYLKRWIYNN 283
12789       LKKGGVDACQGDSGGPMVCESNGAFYLHGATSWGYG-CAAPDKYGVYARVSHLRDWIDQK 467
               ** ..*:.*****:.*:. * : *.*..*** . *:.   * *:*.*  :  ** :

73922       TQQYAAKDGGCGGRSGIVCLNGGTQFCYNKFPTCRCRLGFSGKNCQTGPTNTVNRPSHAN 368
84752       MR---------------------------------------------------------- 285
12789       IASN-------------------------------------------------------- 471
```

Figure S2 Distinct but related genes whose products may be involved in host tolerance of the symbiont. The transcripts differentially expressed between symbiotic and aposymbiotic anemones included two whose top blastx hit in SwissProt was a human peroxidasin and three whose top blastx hit was a mammalian plasma kallikrein (Figure 6; Table S6). (A,B) The two *Aiptasia* peroxidasin-related proteins (Apr1 and Apr2) appear to represent distinct gene products with limited domain homology both to each other and to human peroxidasins. (A) ClustalW sequence alignment of the two *Aiptasia* proteins shows interspersed identical and different amino acids as expected from distinct gene products rather than from alternative splice products or misassembled contigs. Boxes show regions of sequence similarity between the *Aiptasia* proteins but not the human ones (Box 1) or among all four proteins (Boxes 2 and 3), as diagrammed in B. *, :, and . indicate identical, conserved, and semi-conserved amino acids, respectively. (B) Schematic diagram comparing protein domains found in human peroxidasins and the *Aiptasia* peroxidasin-related proteins using Pfam. LRR, leucine-rich repeat; I-set, immunoglobulin I-set; Peroxidase, domain with similarity to canonical peroxidases; VWC, von Willebrand factor type-C; CR, collagen triple-helix repeat; Ig 2, immunoglobulin; F5/8 type C domain (or discoidin domain), with cell-adhesion functions; Pentraxin, domain with similarity to pentraxin pattern-recognition receptors displaying Ca^{2+}-dependent ligand binding. (C) ClustalW sequence alignment of three *Aiptasia* plasma-kallikrein homologues shows interspersed identical and different amino acids as expected from distinct gene products. The box shows the region of sequence similarity representing the shared trypsin-like domain. Symbols are as in A.

Table S1 Correlation between RNA-Seq and RT-qPCR measurements of differential gene expression in symbiotic relative to aposymbiotic anemones.[a]

Locus #/ transcript #	Top Blast Hit	UniProt accession number	Read count [b]	Fold-change (RNA-Seq)	Fold-change (RT-qPCR)
58798/1	Bovine Na^+- and Cl^--dependent taurine transporter	Q9MZ34	61	∞	29
102514/1	Human Npc2 cholesterol transporter	P61916	269	1197	26
95010/1	Mouse tumor necrosis factor receptor superfamily member 27	Q8BX35	202	240	33
125065/1	*Drosophila* organic-cation (carnitine) transporter	Q9VCA2	255	131	57
77179/1	Human scavenger receptor class B member 1 (SRB1; CD36-related)	Q8WTV0	11	28	3.7
95925/1	*Bacteroides thetaiotaomicron* glutamate dehydrogenase	P94598	852	13	2.9
86800/1	Human facilitated glucose transporter (GLUT8)	Q9NY64	57	12	6.3
65589/1	Sheep aquaporin-5	Q866S3	71	11	2.2
70728/1	*C. elegans* NH_4^+ transporter 1 (AMT1-type)	P54145	1382	6.4	7.0
95114/1	Mouse aromatic-amino-acid transporter 1	Q3U9N9	54	5.9	6.2
101012/1	*Bacillus halodurans* isocitrate lyase	Q9K9H0	79	3.9	4.6
66644/1	Human carnitine O-palmitoyltransferase 1	P50416	1237	2.4	2.8
101000/1	*S. cerevisiae* delta(24(24(1)))-sterol reductase	P25340	40	2.0	∞
105631/1	Rat Na^+- and Cl^-dependent GABA transporter 1	P23978	1302	2.0	1.9
125822/1	*Cerberus rynchops* ficolin (collagen/fibrinogen domain containing lectin) 2	D8VNS9	187	1.7	1.8
27493/1	*Salmo salar* Golgi pH regulator	B5X1G3	61	1.2	1.3
12296/1	60*S* ribosomal protein L11	P46222	280	1.1	0.9
119098/1	Rat 40*S* ribosomal protein s7	Q9ZNS1	94	1.0	1.1
12335/1	*Dictyostelium* F-box/WD repeat-containing protein A-like protein	Q54N86	239	-1.0	-1.3
84201/1	*Metridium senile* cytochrome c oxidase	Q35101	1784	-1.4	-1.4
58671/1	*Coturnix japonica* glyceraldehyde-3-phosphate dehydrogenase	Q05025	237	-1.4	-1.1
77428/1	Superoxide dismutase	P81926	987	-1.6	-1.7
21845/2	Rat apoptosis-inducing factor mitochondrial	Q9JM53	769	-1.6	-1.1
59465/1	Rat calmodulin-like protein 3	Q5U206	679	-1.6	-1.5
13527/1	Rat monocarboxylate transporter 10	Q91Y77	47	-1.7	-1.8
12461/1	Rat mannan-binding lectin serine protease 1	Q8CHN8	223	-3.1	-2.8
431/2	Human Na^+/glucose cotransporter 4	Q2M3M2	67	-3.2	-2.0
1568/1	Mouse E2F transcription factor 2	P56931	136	-3.5	-2.1
20440/1	Zebrafish delta-like protein c	Q9IAT6	2	-∞	-1.8

[a] Transcripts are arranged (top to bottom) in order of their degree of expression in symbiotic relative to aposymbiotic anemones as determined by RNA-Seq. Only the data from RNA-Seq Experiment 1 are used, because its conditions matched more closely those of the RT-qPCR experiment (see Materials and Methods and Table 1).

[b] The baseMean expression value as calculated by DESeq (Anders and Huber 2010).

Table S2 Primer sequences and product sizes for RT-qPCR data. [a]

Locus #/ transcript #	Forward Primer	Reverse Primer	Product Size
58798/1	AAAGATCTGCTGGCTGACCCTGA	AACACCAACCAATTGCCTCACCC	134
102514/1	AAGTGACCCGTGCGTTCTCAAA	TGCGTTTGGGTTGGGAATGTGT	148
95010/1	TTTGACATGCTGCGCGAACTGCT	AATGGCCACGACGTGTTTGAAGG	225
125065/1	TGTCAGTGGCGTTGCACAGTCTT	ACATTGCCAATTCTTGCGCGGT	159
77179/1	GAAATGGCGGAAAAAGCATA	GGTGGAAATTGTGTCCCATC	225
95925/1	CAAAGCCTGGACATCGACGCAAA	CAATGACACAGGCCCGCAGAAA	194
86800/1	AGCTGGAGGGAAGGCACCAATAA	TGGGAGCTGTCAATCAACTTGGGA	110
65589/1	TTTGCCGGGAACACGTGCATT	TGAGCGCCGAGTGATGTAGGA	177
70728/1	ACCAACGGATTCCCATTCTCGTCA	TTTGCGGGCAGCAGTGTTGTT	110
95114/1	TGTCGCGCTGTTGCCTTTGTT	TGGCCAAAGCAAGGCGTTTGTGA	187
101012/1	GGTCAGCACGCATGAAAGCATTGT	AAGCAATCCAGATGGCAAAGGCAG	171
66644/1	TCCAAGACCAAGTGTTGGTGGACT	TGATCCAAGTCAGGGACAGGCAAA	110
101000/1	TCTGTCGTGGACACTGCTGTTGA	ATCCAACCGAACTTCTCCGTGGT	189
105631/1	ACCGTGAACACTTCTTGAGAGCCA	GCCTCGGTTGAATGCTTTGTTCGT	210
125822/1	ACCTCGCGCCTTGTCCTTATCAAA	AATGGGACTGTTAAGGCGGTTCGT	225
27493/1	GGTTTGCTGCATCTTCACAGGTCA	AGAAACAGCTGGCGACTAAGCTCT	133
12296/1	AGCCAAGGTCTTGGAGCAGCTTA	TTGGGCCTCTGACAGTACAGTGAACA	125
119098/1	ACTGCAGTCCACGATGCTATCCTT	GTCTGTTGTGCTTTGTCGAGATGC	125
12335/1	TGAAACCTCCTTTCAGCCTCCCA	TCACTTCACTCATCTCGGCAGCA	172
84201/1	AGCAGTTGGTAAGTCTGCACAA	GTAACCATGGTAGCAGCATGAA	105
58671/1	AACAGCTTTGGCAGCACCTGTAGA	TGCTTTCACAGCAACCCAGAAGAC	114
77428/1	AAGGCAAGCGGTAACGAGGTTT	TGCTTTCCTTCTGTCAGCCCAGT	177
21845/2	TCATGGCAAGGACGACGAGTGAA	TCACCCATGGCAGTAAAGAGCGA	156
59465/1	TCGGCAGGATTGTGTCCAAGTGA	AAACGAGCGACACAACGTCAGCA	197
13527/1	AGACACCCAACTGTTCCTTCCCA	ACACGCCGTAAGTAAACGCCAA	212
12461/1	AGCAAAGGGCACGAACAACCAAC	TTGACTCGCTATGGCCGCTAACA	125
431/2	TGGCCTTCAACAAACCTTCACGCT	ACGTTTGTAGTCCCAGCCAGTCA	238
1568/1	AAGTTCGTTGGAGGGTACTGCGA	CCACCAAAGACTTCACACAGCCA	110
20440/1	AATGGCGGAGTTTGTCAAGACGG	TGCCGATGCATTTGCCTGAGTT	118

[a] Transcripts are listed in the same order as in Table S1.

Table S3 Transport-related genes showing differential expression in symbiotic relative to aposymbiotic anemones. [a]

Line	Fold-change [b]	Read count[c]	Locus#/ transcript#	Best BLAST hit	UniProt accession number	BLAST-hit E-value
1	∞	78	58798/1	Bovine Na^+- and Cl^--dependent taurine transporter	Q9MZ34	1e-169
2	∞	81	36456/1	Rabbit Na^+/(glucose/*myo*-inositol) transporter 2	Q28728	3e-104
3	600	659	102514/1	Human Npc2 cholesterol transporter	P61916	2e-14
4	131	437	60777/1	Zebrafish NH_4^+ transporter rh type b	Q7T070	3e-98
5	44	150	125065/1	*Drosophila* organic-cation (carnitine) transporter	Q9VCA2	6e-35
6	28	11	77179/1	Human scavenger receptor class B member 1 (SRB1; CD36-related)	Q8WTV0	9e-65
7	13	70	65589/1	Sheep aquaporin-5	Q866S3	8e-37
8	11	52	86800/1	Human facilitated glucose transporter (GLUT8)	Q9NY64	9e-89
9	6.9	94	12006/1	*Xenopus* GABA and glycine transporter	Q6PF45	8e-60
10	5.9	881	70728/1	*C. elegans* NH_4^+ transporter 1 (AMT1-type)	P54145	6e-72
11	5.8	1667	45451/1	*Drosophila* lipid-droplet surface-binding protein 2	Q9VXY7	2e-08
12	4.9	45	95114/1	Mouse aromatic-amino-acid transporter 1	Q3U9N9	3e-65
13	4.3	198	84722/1	Fish (*Tribolodon*) carbonic anhydrase II	Q8UWA5	2e-36
14	4.3	288	2130/2	Pig aquaporin-3	A9Y006	1e-68
15	3.7	111	11708/1	Human facilitated glucose transporter (GLUT8)	Q9NY64	1e-88
16	3.6	2547	101327/1	Rat neutral- and basic-amino-acid transporter 1	P82252	2e-117
17	3.5	52	103419/1	Chicken monocarboxylate transporter 4 (slc16a3)	P57788	1e-28
18	3.1	71	37788/1	Rabbit hyperpolarization-activated cation channel 4	Q9TV66	9e-129
19	3.1	707	56440/1	Mouse Na^+-independent SO_4^- transporter	Q80ZD3	1e-126
20	2.9	241	97639/1	Bovine ABC subfamily f member 2	Q2KJA2	0
21	2.9	264	11677/1	*Arabidopsis* ABC transporter g family member 27	Q9FT51	7e-67
22	2.7	35	26261/3	*Dictyostelium* UDP-sugar transporter	Q54YK1	1e-32
23	2.6	108	49092/1	Mouse zinc transporter 1 (znt-type)	Q60738	4e-70

24	2.4	61	15916/1	Human major-facilitator-superfamily-domain-containing protein 12	Q6NUT3	9e-37
25	2.4	991	66644/1	Human carnitine *O*-palmitoyltransferase 1	P50416	0
26	2.2	35	119860/1	Mouse Na^+/(glucose/*myo*-inositol) cotransporter 2	Q8K0E3	4e-125
27	2.1	3879	76979/1	Human Na^+-dependent phosphate-transport protein 2b	O95436	2e-112
28	2.1	4804	109479/1	Human neutral- and basic-amino-acid transport protein	Q07837	1e-49
29	2.1	554	12947/1	Rat very-low-density-lipoprotein receptor	P98166	4e-162
30	2.1	53	37499/1	Human aromatic-amino-acid transporter 1	Q8TF71	6e-49
31	2.1	714	14877/3	Zebrafish pyrimidine-nucleotide carrier	Q6DG32	1e-69
32	2.1	243	16360/1	*Xenopus* monocarboxylate transporter 12 (slc16a12)	Q6P2X9	3e-35
33	2.0	88	22123/1	Rat chloride channel clic-like protein	Q9WU61	1e-16
34	2.0	61	120787/1	Mouse aromatic amino acid transporter 1	Q3U9N9	7e-34
35	2.0	989	105631/1	Rat Na^+- and Cl^--dependent GABA transporter 1	P23978	9e-124
36	2.0	231	2338/1	Bovine zinc transporter (zip-type)	A5D7L5	1e-43
37	2.0	582	49156/1	Human ABC subfamily b member 1	P08183	3e-108
38	1.9	1076	86906/1	Human lipid-transfer protein	Q9NQZ5	1e-52
39	1.8	218	120269/1	Mouse Na^+-dependent neutral-amino-acid transporter	O88576	2e-117
40	1.8	1272	36717/5	Rat neutral- and basic-amino-acid transporter 1	P82252	2e-116
41	1.8	381	19286/1	Rat v-ATPase subunit f	P50408	3e-41
42	1.8	211	81279/1	Rat Na^+-dependent phosphate transporter 1	Q9JJP0	2e-71
43	1.7	105	82158/1	*Columba livia* carnitine *O*-acetyltransferase	P52826	5e-151
44	1.7	176	12043/1	Mouse carnitine *O*-palmitoyltransferase 2	P52825	0
45	1.7	1020	70022/1	Rat neutral- and basic-amino-acid transporter 1	P82252	2e-128
46	1.7	193	126133/1	Zebrafish zinc transporter (znt-type)	Q5PQZ3	2e-112
47	1.6	1058	33916/1	Human transitional-ER ATPase	P55072	0
48	1.6	479	86475/1	*Xenopus* peptide transporter 4	Q68F72	1e-98
49	-1.6	164	22834/1	Chicken monocarboxylate transporter 3	Q90632	2e-30
50	-1.6	684	108875/1	Chicken low-density-lipoprotein receptor-	P98157	0

				related protein 1		
51	-1.6	404	28605/1	Rat TRP cation-channel subfamily a, member 1	Q6RI86	1e-115
52	-1.8	1323	33284/1	Mouse aromatic amino acid transporter 1	Q3U9N9	3e-34
53	-1.8	5989	71915/1	Rabbit non-specific lipid-transfer protein	O62742	0
54	-1.8	817	16745/1	Rat plasma membrane Ca^{2+}-transporting ATPase	P11505	3e-98
55	-2.0	39	13527/1	Rat monocarboxylate transporter 10 (aromatic amino acid transporter 1)	Q91Y77	6e-19
56	-2.1	33	122320/1	Human long-chain fatty acid transport protein 1	Q6PCB7	3e-96
57	-2.2	848	43841/1	Chicken ovotransferrin	P02789	3e-47
58	-2.3	4512	98994/1 [d]	Human Npc2 cholesterol transporter	P61916	5e-09
59	-2.4	5556	44110/1	Human low-density lipoprotein receptor-related protein 4	O75096	8e-61
60	-2.8	40	76106/1	*C. elegans* TRP-like cation channel protein 1	P34586	3e-55
61	-2.9	190	93152/1	Chimpanzee NH_4^+ transporter rh type c	Q3BCQ7	5e-111
62	-2.9	629	104248/1	Rat serotransferrin	P12346	2e-47
63	-3.0	506	76019/1	Carbonic anhydrase	P83299	1e-37
64	-3.5	1893	56973/1	Mouse organic cation carnitine transporter 3	Q9WTN6	5e-31
65	-3.5	301	432/1	Human Na^+/glucose cotransporter 4	Q2M3M2	2e-130
66	-3.8	325	129624/1	*E. coli* high-affinity choline-transport protein	P0ABD0	2e-78

[a] Putative small-molecule transporters and some proteins of related function (see text) are arranged in order of their degree of differential expression in symbiotic anemones relative to aposymbiotic anemones. Positive fold-changes, expression higher in symbiotic anemones; negative fold-changes, expression higher in aposymbiotic anemones.

[b] The arithmetic mean of the values from the two RNA-Seq experiments, except in line 6 (transcript 77179/1). ∞, expression was not detected in aposymbiotic animals. Transcript 77179/1 was not detected in aposymbiotic anemones in Experiment 2, giving a nominal ∞-fold change in expression. However, as the normalized read counts in both experiments were rather low, and the possible involvement of the 77179/1-encoded protein in lipid metabolism makes it likely to have been affected in its expression by the starvation conditions used in Experiment 2, we report in line 6 the more conservative value from Experiment 1 alone.

[c] Except for line 6, the average of the baseMean expression values (as calculated by DESeq (Anders and Huber 2010)) for Experiment 1 and Experiment 2. As explained in footnote b, for transcript 77179/1 (line 6), we show the value for Experiment 1 alone.

[d] Appears to represent a truncated version of transcript 98999/1, whose predicted protein product was used for the phylogenetic analysis of Figure 2A.

Table S4 Lipid-metabolism genes showing differential expression in symbiotic relative to aposymbiotic anemones. [a]

Line	Metabolic Process	Putative protein function (best BLAST hit)	UniProt accession number	BLAST-hit E-value	Locus #/ transcript #	Fold-change [b]
1	FA synthesis	ACC1: Acetyl-CoA carboxylase (chicken)	P11029	0	26166/1	3.9
2	FA synthesis	ELOVL4: Elongation-of-very-long-chain-fatty-acid protein 4 (mouse)	Q9EQC4	1e-23	4012/1	4.2
3	FA synthesis	Δ^5 fatty-acid desaturase (*Mortierella alpina*)	O74212	6e-39	120701/1	6.2
4	FA synthesis	Δ^6 fatty acid desaturase (human)	O95684	6e-46	92492/1	3.5
5	Lipid storage	DHAPAT: dihydroxyacetone phosphate acyltransferase (human)	O15228	1e-95	8091/1	1.4
6	Lipid storage	2-acylglycerol *O*-acyltransferase 2-a (*Xenopus*)	Q2KHS5	8e-75	10118/2	2.7
7	Lipid storage	2-acylglycerol *O*-acyltransferase 2-a (*Xenopus*)	Q2KHS5	4e-89	15365/1	-2.0
8	Lipid storage	2-acylglycerol *O*-acyltransferase 2-b (*Xenopus*)	Q5M7F4	9e-81	78512/1	2.4
9	Lipid storage	Diacyglycerol *O*-acyltransferase (*Mycobacterium tuberculosis*)	O06795	1e-19	76581/1	2.1
10	Lipid storage	AGPAT 1: 1-acyl-*sn*-glycerol-3-phosphate acyltransferase alpha (human)	Q99943	2e-23	67491/1	-1.4
11	Lipid storage regulation	Lipid-droplet surface-binding protein 2 (*Drosophila*)	Q9VX7	2e-08	45451/1	5.8
12	Lipase	HSL: Hormone-sensitive lipase (human)	Q05469	2e-84	13988/1	1.8
13	Lipase	ATGL: Adipose triglyceride lipase (mouse)	Q8BJ56	9e-62	16411/1	-3.3
14	FA transport	FATP1: Fatty-acid transport protein 1 (human)	Q6PCB7	3e-96	122320/1	-1.9
15	FA transport	FATP4: Long-fatty-acid transport protein 4 (orangutan)	Q5RDY4	8e-92	122313/8	-3.1
16	FA transport	SRB1: Scavenger receptor class B member 1 (human; CD36-related protein)	Q8WTV0	9e-65	77179/1	28
17	FA-CoA-ligase	ACSL4: Long-chain-fatty-acid ligase 4 (human)	O60488	0	89704/1	5.7
18	FA-CoA-ligase	ACSL5: Long-chain-fatty-acid ligase 5 (human)	Q9ULC5	5e-116	106694/1	2.9
19	FA β-oxidation	Organic cation (carnitine) transporter (*Drosophila*)	Q9VCA2	6e-35	125065/1	44
20	FA β-oxidation	CPT1: Carnitine *O*-palmitoyltransferase 1 (human)	P50416	0	66644/1	2.4
21	FA β-oxidation	CPT2: Carnitine *O*-palmitoyltransferase 2 (mouse)	P52825	0	12043/1	1.6
22	FA β-oxidation	CACT: Carnitine acylcarnitine carrier protein (bovine)	Q08DK7	2e-60	13918/1	n.s.
23	FA β-oxidation	VLCAD: Very-long-chain-specific acyl-CoA dehydrogenase (bovine)	P48818	0	108541/1	1.6
24	FA β-oxidation	MTP: Trifunctional enzyme (pig)	Q29554	0	33057/3	1.4
25	FA β-oxidation	MCAD: Medium-chain-specific acyl-CoA dehydrogenase (bovine)	Q3SZB4	5e-165	92556/1	n.s.
26	FA β-oxidation	SCAD: Short-branched-chain-specific acyl-CoA dehydrogenase (*Dictyostelium*)	Q54RR5	2e-121	127382/1	n.s.
27	FA β-oxidation	Crotonase: Short-chain enoyl-CoA hydratase (*Dictyostelium*)	Q1ZXF1	4e-67	117452/1	n.s.
28	FA β-oxidation	M/SCHAD: Medium and short-chain l-3-	Q16836	5e-29	34949/1	n.s.

		hydroxyacyl-CoA dehydrogenase (human)				
29	FA β-oxidation	DCI: Enoyl-Δ isomerase (human)	P42126	7e-33	55782/1	n.s.
30	FA β-oxidation	MCKAT: 3-ketoacyl-CoA thiolase (rat)	P13437	5e-145	56206/1	2.4
31	Glyoxylate cycle	Isocitrate lyase (*Bacillus halodurans*)	Q9K9H0	3e-164	101012/1	3.9
32	Glyoxylate cycle	Malate synthase (*Myxococcus xanthus*)	P95329	2e-141	22622/1	n.s.

[a] Genes encoding proteins putatively involved in lipid metabolism are arranged in groups by biological process (see Figure 3). FA, fatty acid.

[b] Because of the likelihood that the starvation conditions in Experiment 2 would affect lipid metabolism, the fold-change values from Experiment 1 are shown. Positive fold-changes, expression higher in symbiotic anemones; negative fold-changes, expression higher in aposymbiotic anemones. n.s., no significant differential expression observed.

Table S5 Presence or absence in the *Aiptasia* transcriptome of genes encoding the enzymes involved in the synthesis of particular amino acids. [a]

Line	Amino acid	Enzyme	UniProt accession number	*Aiptasia* locus #/transcript #
1	Gln	Glutamine synthetase	P32288	104234/1
2	Glu	Glutamate synthase	Q12680	60857/1
3	Glu/Pro	NADP-specific glutamate dehydrogenase [b]	Q9C8I0	3911/1
4	Glu/Pro	NADP-specific glutamate dehydrogenase 2 [b]	P39708	95925/1
5	Glu/Pro	NAD-specific glutamate dehydrogenase [c]	P33327	99746/1
6	Glu/Pro	Glutamate dehydrogenase 2 [c]	Q38946	7229/1
7	Met	MTHFR: Methylenetetrahydrofolate reductase	Q9WU20	15095/1
8	Met	MS: Methionine synthase (cobalamin-dependent)	Q99707	50131/1
9	Met	Methionine synthase (cobalamin-independent) [d]	P05694	55393/1
10	Met	BHMT: Betaine-homocysteine *S*-methyltransferase 1	Q93088	45257/1
11	Cys/SAM [e]	MAT: Methionine adenosyltransferase 1	Q91X83	140402/1
12	Cys	SAHH: *S*-adenosyl-L-homocysteine hydrolase	P27604	91092/1
13	Cys	CBS: Cystathionine β-synthase	P32582	98284/1
14	Cys	CGL: Cystathionine γ-lyase	P31373	7792/1
15	Met/Cys/Thr/Ile/ Lys	Aspartokinase/homoserine dehydrogenase	Q9SA18	
16	Met/Cys/Thr/ Ile/Lys	Aspartokinase	P10869	
17	Met/Cys/Thr/ Ile	Homoserine dehydrogenase	P31116	
18	Met/Cys	HAT: Homoserine *O*-acetyltransferase	P08465	70690/1
19	Met/Cys	CGL: Cystathionine γ-synthase	P47164	974/1
20	Met	Cystathionine β-lyase	P43623	
21	Met	Homocysteine *S*-methyltransferase 3	Q8LAX0	4396/6
22	Ser	D-3-phosphoglycerate dehydrogenase 1	P40054	294/1
23	Ser	Phosphoserine aminotransferase	P33330	57256/1
24	Ser	Phosphoserine phosphatase	P42941	122485/1
25	Ser	Catabolic L-serine/threonine dehydratase	P25379	21690/1
26	Ser/Gly	Serine hydroxymethyltransferase, mitochondrial	P37292	11787/1
27	Ser/Gly	Serine hydroxymethyltransferase, cytosolic	P37291	11787/1
28	Gly	Alanine-glyoxylate aminotransferase 1	P43567	47533/1
29	Gly	Serine-glyoxylate aminotransferase	Q56YA5	47531/1
30	Gly	Low specificity L-threonine aldolase	P37303	109186/1
31	Asp/Glu/Asn	Aspartate aminotransferase, mitochondrial	Q01802	23248/1
32	Asp/Glu/Asn	Aspartate aminotransferase, cytoplasmic	P46646	111366/1
33	Asn	Asparagine synthetase	P49089	51175/1
34	Ala	Alanine aminotransferase 1	P52893	89107/1
35	Pro	γ-glutamyl phosphate reductase	P54885	128220/1
36	Pro	Pyrroline-5-carboxylate reductase	P32263	115939/1
37	Arg	Caramoyl-phosphate synthetase	P31327	21357/1
38	Arg	Ornithine carbamoyltransferase	P00480	116500/1
39	Arg	Argininosuccinate synthetase	P22768	53174/1
40	Arg	Argininosuccinate lyase	P04076	29236/1
41	Arg	Arginase-1	P05089	118787/1
42	Arg	*N*-acetylglutamate synthase	Q8N159	19094/1
43	Arg	Acetylglutamate kinase	Q01217	
44	Arg	Ornithine acetyltransferase	Q04728	

45	Phe/Tyr	Aromatic/aminoadipate aminotransferase 1	P53090	109224/1
46	Tyr	Tyrosine aminotransferase	Q9LVY1	58220/1
47	Tyr	Phenylalanine 4-hydroxylase	P00439	37855/1
48	Phe/Trp	Class-II DAHP synthetase-like protein	Q9SK84	
49	Phe/Trp	Phospho-2-dehydro-3-deoxyheptonate aldolase, tyrosine-inhibited	P32449	
50	Phe/Trp	Pentafunctional AROM polypeptide	P08566	
51	Phe/Trp	Chorismate mutase	P32178	
52	Phe/Trp	Chorismate synthase	P28777	
53	Phe/Trp	Anthranilate synthase component 1	P00899	
54	Phe/Trp	Anthranilate phosphoribosyltransferase	P07285	
55	Trp	Tryptophan synthase	Q42529	
56	His	ATP phosphoribosyltransferase	P00498	
57	His	Imidazole glycerol phosphate synthase hisHF	P33734	
58	His	Histidinol-phosphate aminotransferase	P07172	
59	His	Histidine biosynthesis trifunctional protein	P00815	
60	His	Histidinol dehydrogenase	Q9C5U8	
61	Val/Leu/Ile	Acetolactate synthase	P07342	8385/1
62	Val/Leu/Ile	Ketol-acid reductoisomerase, mitochondrial	P06168	
63	Val/Leu/Ile	Dihydroxy-acid dehydratase, mitochondrial	P39522	127954/1
64	Val/Leu/Ile	Branched-chain-amino-acid aminotransferase, cytosolic	P47176	85088/1
65	Leu	2-isopropylmalate synthase	P06208	
66	Leu	3-isopropylmalate dehydratase	P07264	
67	Leu	3-isopropylmalate dehydrogenase	P04173	
68	Ile	Threonine dehydratase, mitochondrial	Q9ZSS6	57366/1
69	Lys	Homocitrate synthase, mitochondrial	Q12122	
70	Lys	Kynurenine/α-aminoadipate aminotransferase,mitochondrial	Q8N5Z0	
71	Lys	Homoaconitase, mitochondrial	P49367	
72	Lys	Homoisocitrate dehydrogenase, mitochondrial	P40495	
73	Lys	L-aminoadipate-semialdehyde dehydrogenase	P07702	127184/1
74	Lys	Saccharopine dehydrogenase [NADP(+), L-glutamate-forming]	P38999	1580/1
75	Lys	Saccharopine dehydrogenase [NAD(+), L-lysine-forming]	P38998	76032/1
76	Lys	4-hydroxy-tetrahydrodipicolinate synthase 2, chloroplastic	Q9FVC8	
77	Lys	Dihydrodipicolinate synthase	Q0WSN6	
78	Lys	Diaminopimelate decarboxylase 1	Q949X7	37096/1? [f]
79	Thr	Threonine synthase	P16120	10016/1

[a] The UniProt Accession Number shown is for the seed sequence used to identify the *Aiptasia* transcript. For an *Aiptasia* transcript to be listed, its best reciprocal BLAST hit (to the same species as the seed sequence) had to be the seed sequence itself or to a sequence encoding a paralogous protein. Where no transcript is listed, no *Aiptasia* homologue of the seed sequence could be identified with confidence.
[b] Downregulated (transcript 3911/1) and upregulated (95925/1) in symbiotic relative to aposymbiotic anemones (see Figure 4). Both proteins had a *Bacterioides thetaiotaomicron* NAD(P)-utilizing glutamate dehydrogenase (UniProt P94598) as their top BLAST hit.
[c] No significant differential expression in symbiotic relative to aposymbiotic anemones.
[d] In contrast to transcript 50131/1 (MS in Figure 5), transcript 55393/1 showed no differential expression in symbiotic *vs.* aposymbiotic anemones.
[e] *S*-adenosyl-methionine (see Figure 5).
[f] Although this *Aiptasia* transcript met the formal criterion for inclusion (footnote a), the number of genomic reads mapping to it barely exceeded our cut-off for calling a sequence cnidarian (see Table 2), so that it may represent a contaminant rather than an *Aiptasia* gene encoding a homologue of this typically bacterial and plant enzyme.

Table S6 Genes potentially involved in host tolerance of the symbiont that are differentially expressed between symbiotic and aposymbiotic anemones. [a]

Protein (from top BLAST hit)	UniProt accession number	Locus #/ transcript #	BLAST-hit E-value	Fold-Change [b]
A. Response to oxidative stress				
Catalase	P04040	100968/1	0	-4.7
ADAM (disintegrin and metalloproteinase domain-containing protein) 9	Q13443	123296/1	2e-62	-3
Transient receptor potential cation channel (subfamily M, member 2)	Q91YD4	125627/1	7e-16	-2.9
Peroxidasin-related protein 1	Q92626	99631/1	4e-06	-2.5
Dual oxidase 2	Q8HZK2	17080/2	5e-27	-2
Allene oxide synthase-lipoxygenase	O16025	9291/1	4e-38	-1.8
Soluble guanylate cyclase 88E	Q8INF0	7254/1	1e-171	1.9
Peroxidasin-related protein 2	A1KZ92	57146/1	7e-25	2.9
B. Inflammation/tissue remodeling/response to wounding				
Transmembrane serine protease 6 [c]	Q9DBI0	81296/1	3e-50	-6.1
Plasma kallikrein [d]	P14272	12789/1	7e-47	-4
Mannan-binding lectin serine peptidase 1 (MASP-1) [e]	Q8CHN8	36375/1	5e-12	-3
Plasminogen [f]	P00747	21286/1	3e-47	-3
Plasma kallikrein [d]	P03952	84752/1	1e-48	-2.9
Ephrin type-a receptor 3 [g]	P29320	6695/1	2e-63	-2.8
Phospholipase A2 (isoform 4) [h]	Q6T179	3740/5	1e-26	-2.4
Arachidonate 5-lipoxygenase [i]	P48999	55879/1	5e-28	-1.7
Plasma kallikrein [d]	P26262	73922/1	8e-40	2.1
Ficolin 2 [j]	Q15485	62279/1	1e-42	2.2
Vanin-I [k]	Q58CQ9	48344/1	8e-118	2.6
Discoidin, CUB, and LCCL domain containing 2 [l]	Q91ZV2	80843/1	3e-12	2.7
Hepatocyte nuclear factor 4 (alpha) [m]	P22449	34830/4	1e-113	3.6
Adenosine A2b receptor [n]	O13076	66307/1	2e-17	4.2
Scavenger receptor class B member 1 [o]	Q8WTV0	77179/1	9e-65	28
C. Apoptosis/cell death				
Transcription factor E2F2	P56931	1568/1	5e-07	-4.2
Receptor-binding cancer antigen expressed on SiSo cells	Q865S0	42283/1	4e-06	-2.7
Tumor protein p73	Q9JJP2	88973/1	2e-35	-2.4
Paired box protein Pax-3	P23760	46973/1	3e-43	-1.8
Apoptosis-inducing factor 1 (mitochondrial)	Q9JM53	21845/2	1e-171	-1.8
TNF (Tumor Necrosis Factor) receptor associated factor 3	Q13114	30586/1	2e-74	1.8
TNF superfamily member 12	O43508	18277/1	1e-07	1.9

Kruppel-like factor 11	O14901	58173/1	8e-51	2.6
G1 to S phase transition 1	P15170	25564/1	0	2.8
Growth arrest and DNA damage-inducible protein (GADD45 gamma)	Q9Z111	55453/1	6e-09	5.1
Ribonucleoside-diphosphate reductase (small chain C)	Q9LSD0	18748/1	1e-132	12
Organic cation transporter	Q9VCA2	88336/1	6e-35	44
TNF receptor superfamily member 27	Q8BX35	94982/1	9e-10	60

[a] The set of all transcripts displaying differential expression by RNA-Seq was analyzed to identify biological processes (based on GO terms) that were overrepresented in this set relative to the background transcriptome (see Materials and Methods). The sets of processes identified here (A, B, and C) emerged from this analysis and may be involved in host tolerance of the symbiont.

[b] In all but one case, the arithmetic mean of the values from the two RNA-Seq experiments is shown. For transcript 77179/1 (last line of section B), the value from Experiment 1 is shown for reasons explained in Table S3, footnote b. Positive fold-changes, expression higher in symbiotic anemones; negative fold-changes, expression higher in aposymbiotic anemones.

[c] Hydrolyzes a range of proteins including type I collagen, fibronectin, and fibrinogen and may play a role in matrix-remodeling processes (Hooper *et al.* 2003).

[d] Serine proteases activated by tissue injury or microbial invasion; they activate the release of potent pro-inflammatory cytokines that ultimately result in the release of effector molecules such as nitric oxide and tumor necrosis factor-α and can stimulate the complement innate-immunity system (Lalmanach *et al.* 2010; Moreau *et al.* 2005).

[e] Plays a role as an amplifier of the complement cascade, potentially via the activation of MASP-2 (Takahashi *et al.* 2008).

[f] The zymogen of plasmin; it can be activated via plasma kallikrein and functions in the breakdown of fibrin in fibrinolysis, the activation of proteases, and the modulation of cell adhesion (Li *et al.* 2003).

[g] A receptor tyrosine kinase that binds membrane-bound ephrin family ligands residing on adjacent cells and regulates cell-cell adhesion, cytoskeletal organization, and cell migration (Smith *et al.* 2004).

[h] Releases arachidonic acid from cellular membrane phospholipids, leading to its conversion to pro-inflammatory prostaglandins via arachidonate 5-lipoxygenase (Moreau *et al.* 2005).

[i] See note h.

[j] A lectin whose binding to microbial surface glycans can initiate activation of the complement pathway (Endo *et al.* 2007); it also appears to bind to cell-surface glycans of *Symbiodinium* (Logan *et al.* 2010).

[k] Hydrolyzes pantetheine to pantothenic acid and cysteamine, the latter of which can lead to acute and chronic epithelial inflammation (Martin *et al.* 2004).

[l] Thought to play a role in cell adhesion and wound healing (Kobuke *et al.* 2001).

[m] A transcriptional regulator that is decreased in inflammatory bowel disease and protects against chemically-induced colitis in mice (Darsigny *et al.* 2009).

[n] Its activation appears to result in inhibition of pro-inflammatory cytokine production, and mice deficient in A2b receptors are more susceptible to intestinal inflammation (Gessi *et al.* 2011).

[o] See text.

File S1

Supplementary Materials and Methods

Identification and Optimization of qPCR Standards for *Aiptasia*

Six housekeeping genes were selected as potential qPCR standards based on their prior use in coral studies. Gene names used here are those assigned to the *Aiptasia pallida* genes and differ in most cases from those used in the other organisms. The genes encoding 60*S* ribosomal protein L11 (*RPL11*), NADH-dehydrogenase subunit 5 (*NDH5*), and glyceraldehyde-3-phosphate-dehydrogenase (*GPD1*) were reported to be stable in *Porites astreoides* during heat stress, settlement induction, and metamorphosis (Kenkel *et al.* 2011). The genes encoding 40*S* ribosomal protein S7 (*RPS7*) and adenosylhomocysteinase (*AHC1*) were used as standards during studies of thermal stress in *Acropora aspera* (Leggat *et al.* 2011). The β-actin gene (*ACT1*) was used to explore modulation of host-gene expression (Rodriguez-Lanetty *et al.* 2006) and was used as the standard for early qPCR studies in our lab.

Primers were developed and tested for these six potential standard genes. The aposymbiotic *A. pallida* transcriptome (Lehnert *et al.* 2012) was searched using tblastx with sequences from *Porites lobata* for *NDH5*, *P. astreoides* for *RPL11*, *Urticina eques* for *GPD1*, *Acropora millepora* for *RPS7*, and *Nematostella vectensis* for *AHC1*. The loci identified in the *A. pallida* transcriptome were searched using blastx in NCBI and all top hits were indeed the genes of interest. The identified loci were then translated using ORFPredictor and the longest ORFs were used to identify conserved sequences by performing protein alignments in MacVector with sequences available from NCBI. Conserved sequences were then used to develop primers using PrimerQuest from Integrated DNA Technologies (IDT).

Primers were tested on *A. pallida* cDNA and gDNA. Primers that spanned an exon-intron junction were preferentially identified for further use (Table S7). PCR products were cloned into a TA cloning vector and electroporation-competent *E. coli* cells were transformed with the plasmids. Transformed cells were plated on Ampicillin/X-Gal plates and white/light-blue colonies were selected for colony PCR using M13 forward and reverse primers. PCR products were sequenced, and the sequences were aligned with the expected sequences from the transcriptome. All primer pairs accurately selected the sequences of interest.

Table S7 Primer sequences used for potential qPCR standards

Gene	Primer sequences
RPL11	F: AGCCAAGGTCTTGGAGCAGCTTA<br>R: TTGGGCCTCTGACAGTACAGTGAACA
RPS7	F: ACTGCAGTCCACGATGCTATCCTT<br>R: GTCTGTTGTGCTTTGTCGAGATGC
NDH5	F: AGCAGTTGGTAAGTCTGCACAA<br>R: GTAACCATGGTAGCAGCATGAA
GPD1	F: AACAGCTTTGGCAGCACCTGTAGA<br>R: TGCTTTCACAGCAACCCAGAAGAC
AHC1	F: CCATTACAGCAACAACACAGGCCA<br>R: GCATCAAACGTTGGCAGATGAAGC
ACT1	F: ACACCGTCTTGTCAGGAGGTTCAA<br>R: TCCACATCTGTTGGAAGGTGGACA

The six genes were then tested for their expression levels across 11 experimental conditions (Table S8). RNA was extracted from 3-4 medium-sized anemones from each condition using a Trizol/RNeasy hybrid protocol (details available upon request). RNA integrity was checked both by using a Nanodrop and by running samples on a 2% agarose gel. For all RNA samples used, 260/280 readings were >1.9, and two clear rRNA bands were visible. For each condition, 300 ng of RNA was reverse transcribed using the Maxima® First Strand cDNA-synthesis kit for RT-qPCR (Fermentas). 17 µL of RT product was then diluted with 23 µL of H_2O. 2 µL of this cDNA solution was then used for the qPCR reaction. Each qPCR well had 2 µL of cDNA, 2 µL of H_2O, 5 µL of *Power* SYBR® Green PCR Master Mix (Applied Biosystems), and 1 µL of a primer mix containing 1.5 µM forward (F) primer and 1.5 µM reverse (R) primer.

The primer efficiency of each primer pair was tested across a dilution series of 1:1, 1:10, 1:100, 1:1000, and 1:10000 cDNA; the calculated efficiencies were 95-105%. Possible gDNA contamination in RNA samples was tested by running RNA-only controls; these samples showed no amplification. Standard qPCR settings were used, and an additional dissociation stage was added to test for the presence of multiple products. The dissociation stage showed only one clear peak in every case.

Table S8 Experimental conditions used to test gene-expression levels by qPCR

Conditions [a]	CC7 Sym [b]	CC7 Apo [c]
Room Temperature (27ºC)	x	x
1 h heat shock (35ºC)	x	x
1.5 h heat shock (37ºC)	x	x
1 h cold shock (8ºC)	x	x
1 h incubation with 500 µg/mL dsRNA[d] (27ºC)	x	x
Kept in the dark for 1 month (27ºC)	x [e]	not done

[a] Except for the sample incubated in the dark, all anemones were incubated on a 12L:12D cycle with 25 µmol photons m^{-2} s^{-1} from Cool White fluorescent bulbs, and the manipulations indicated were performed during the light period.
[b] Symbiotic anemones (containing the endogenous population of Clade A *Symbiodinium*) of the CC7 clonal line of *Aiptasia* (Sunagawa *et al.* 2009).
[c] Aposymbiotic CC7 animals that had been cured of their endogenous *Symbiodinium* by a combination of cold shock, DCMU treatment, and extended growth in the dark (Lehnert *et al.* 2012). All anemones were screened for absence of dinoflagellates prior to use in these experiments.
[d] dsRNA (477 bp) synthesized for *A. pallida* nematogalectin gene knockdown.
[e] Represents a partially aposymbiotic condition.

Ct values for each of the six genes under each of the 11 conditions were analyzed using geNorm (Vandesompele *et al.* 2002) to determine the relative expression stabilities of the prospective standard genes; the M-values are inversely proportional to the stabilities of the genes (Table S9). *ACT1* (M = 0.625) and *AHC1* (M = 0.775) were considerably less stable in expression than the four genes shown in the table.

Statistical analysis of the qPCR results also indicates that *ACT1* should not be used as an expression standard in the study of symbiosis in *Aiptasia* due to the large expression difference between aposymbiotic and symbiotic animals: there was a significant ($p = 0.002$) up-regulation in *ACT1* expression in aposymbiotic (or mostly aposymbiotic) anemones compared to symbiotic anemones across all conditions. This was determined by normalizing qPCR Ct values with the two most stable standard genes (*RPL11* and *RPS7*) and performing a Mann-Whitney statistical test on *ACT1* expression levels in aposymbiotic and symbiotic anemones.

Table S9 Assessment of gene-expression stability under various conditions [a]

Gene	Protein encoded	geNorm M	Product Sequence	Product Length	Primer Efficiency
RPL11	Component of the 60S ribosomal subunit	0.357	AGCCAAGGTCTTGGAGCAGCTTACAGGC CAACAGCCTGTGTTTTCAAAAG **(INTRON – 236 bp)** CTCGCTACACTGTGAGATCTTT TGGAATCAGAAGGAACGAGAAGATCTCT GTTCACTGTACTGTCAGAGGCCCAA	cDNA 125 bp<br>gDNA 361 bp	98%
RPS7	Component of the 40S ribosomal subunit	0.380	ACTGCAGTCCACGATGCTATCCTTGAAGA TCTTGTCTTTCCTAGTGAAATTGTTGGCAA AAGGATAAGAGTTAAACTTGATGGTTCAC GTCTCGTTAAAGTG **(INTRON – 411 bp)** CATCTCGACAAAGCACAACAGAC	cDNA 125 bp<br>gDNA 536 bp	97%
NDH5	NADH-dehydrogenase subunit 5	0.423	AGCAGTTGGTAAGTCTGCACAATTAGGCT TACACACTTGGTTACCGGATGCAATGGAA GGT **(INTRON – 1729 bp)** CCAACTCCGG TGTCTGCCTTGATTCATGCTGCTACCATGG TTAC	cDNA 105 bp<br>gDNA 1834 bp	95%
GPD1	Glyceraldehyde-3-phosphate-dehydrogenase	0.530	AACAGCTTTGGCAGCACCTGTAGAGGCTG GGATGATATTCTGATTGGCACCTCTACCA TCACGCCATTTCT **(INTRON – 567 bp)** TCCCACTAGGTCCATCTACAGTCTTCTGGG TTGCTGTGAAAGCA	cDNA 114 bp<br>gDNA 681 bp	95%

[a] Tested across the 11 experimental conditions described in Table S8.

Accession numbers for the sequences used in developing the training and test sets for TopSort

Cnidarian dataset: *Nematostella vectensis* (AB126336.1-AB126336.1, AB450038.1-AB450044.1, AB479470.1-AB479474.1, AB495365.1-AB495368.1, AF020956.1-AF020964.1, AF085282.1-AF085283.1, AY286508.1-AY286510.1, AY339866.1-AY339873.1, AY391716.1-AY391717.1, AY465174.1-AY465182.1, AY496945.1-AY496946.1, AY496948.1-AY496949.1, AY530300.1-AY530301.1, AY687348.1-AY687350.1, AY725201.1-AY725205.1, AY730689.1-AY730697.1, DQ066724.1-DQ066725.1, DQ116032.1-DQ116034.1, DQ173687.1-DQ173698.1, DQ358699.1-DQ358704.1, DQ471325.1-DQ471326.1, DQ492688.1-DQ492689.1, DQ493899.1-DQ493901.1, DQ497246.1-DQ497247.1, DQ517920.1-DQ517928.1, DQ826414.1-DQ826417.1, DQ882654.1-DQ882656.1, EF068140.1-EF068151.1, EF173462.1-EF173463.1, EF424410.1-EF424412.1, EU092640.1-EU092641.1, EU162649.1-EU162655.1, EU394531.1-EU394532.1, EU422968.1-EU422972.1, EU877197.1-EU877198.1, FJ824849.1-FJ824851.1, GQ240844.1-GQ240851.1, GU320063.1-GU320067.1, HM004556.1-HM004558.1, HM754642.1-HM754644.1, XM_001617352.1-XM_001642094.1, U42728.2, FJ428244.1, EU289217.1, EF427936.1, DQ632751.1, DQ286294.1, DQ198160.1, AY792510.1, AY651960.1, AY534532.1, AY494080.1, AY457634.1, AY363391.1, AY226090.1, AY226076.1, AY226067.1, AY226056.1, AF540387.2, AF408421.1, AF327845.1, AB495363.1, AB274036.1, AB274034.1); *Hydra magnipapillata* (AB583744.1-AB583747.1, AM233901.1-AM233903.1, AM393878.1-AM393881.1, AY212265.1-AY212267.1, AY218839.1-AY218840.1, BK004161.1-BK004162.1, DQ073557.1-DQ073558.1, DQ127903.1-DQ127904.1, DQ449927.1-DQ449931.1, EU170504.1-EU170505.1, FJ156099.1-FJ156102.1, FJ177032.1-FJ177033.1, FJ196704.1-FJ196706.1, FJ200200.1-FJ200210.1, FJ205481.1-FJ205489.1, FJ236863.1-FJ236864.1, FJ496649.1-FJ496653.1, FJ517724.1-FJ517728.1, GQ856263.1-GQ856264.1, GU219979.1-GU219981.1, GU256274.1-GU256281.1, XM_002153740.1-XM_002153922.1, XM_002153924.1-XM_002154094.1, XM_002154096.1-XM_002154206.1, XM_002154208.1-XM_002154426.1, XM_002154428.1-XM_002154512.1, XM_002154514.1-XM_002154764.1, XM_002154766.1-XM_002154895.1, XM_002154897.1-XM_002154984.1, XM_002154986.1-XM_002155429.1, XM_002155431.1-XM_002155750.1, XM_002155752.1-XM_002156047.1, XM_002156049.1-XM_002156748.1, XM_002156750.1-XM_002157387.1, XM_002157389.1-XM_002157474.1, XM_002157476.1-XM_002158411.1, XM_002158413.1-XM_002158516.1, XM_002158518.1-XM_002158837.1, XM_002158839.1-XM_002159264.1, XM_002159266.1-XM_002159291.1, XM_002159293.1-XM_002159320.1, XM_002159322.1-XM_002159398.1, XM_002159400.1-XM_002159430.1, XM_002159432.1-XM_002159454.1, XM_002159456.1-XM_002159503.1, XM_002159505.1-XM_002159563.1, XM_002159565.1-XM_002159607.1, XM_002159609.1-XM_002159628.1, XM_002159630.1-XM_002159660.1, XM_002159662.1-XM_002159732.1, XM_002159734.1-XM_002159756.1, XM_002159758.1-XM_002159789.1, XM_002159791.1-XM_002159832.1, XM_002159834.1-XM_002159873.1, XM_002159875.1-XM_002159897.1, XM_002159899.1-XM_002159921.1, XM_002159923.1-XM_002159938.1, XM_002159940.1-XM_002159972.1, XM_002159974.1-XM_002159999.1, XM_002160001.1-XM_002160050.1, XM_002160052.1-XM_002160081.1, XM_002160083.1-XM_002160109.1, XM_002160111.1-XM_002160170.1, XM_002160172.1-XM_002160207.1, XM_002160209.1-XM_002160254.1, XM_002160256.1-XM_002160282.1, XM_002160284.1-XM_002160328.1, XM_002160330.1-XM_002160388.1, XM_002160390.1-XM_002160464.1, XM_002160466.1-XM_002160488.1, XM_002160490.1-XM_002160516.1, XM_002160518.1-XM_002160520.1, XM_002160522.1-XM_002160546.1, XM_002160548.1-XM_002160549.1, XM_002160551.1-XM_002160590.1, XM_002160592.1-XM_002160609.1, XM_002160611.1-XM_002160643.1, XM_002160645.1-XM_002160677.1, XM_002160679.1-XM_002160736.1, XM_002160738.1-XM_002160762.1, XM_002160764.1-XM_002160793.1, XM_002160795.1-XM_002160812.1, XM_002160814.1-XM_002160825.1, XM_002160827.1-XM_002160916.1, XM_002160918.1-XM_002160934.1, XM_002160936.1-XM_002160939.1, XM_002160941.1-XM_002160987.1, XM_002160989.1-XM_002161016.1, XM_002161018.1-XM_002161068.1, XM_002161070.1-XM_002161111.1, XM_002161113.1-XM_002161217.1, XM_002161219.1-XM_002161238.1, XM_002161240.1-XM_002161303.1, XM_002161305.1-XM_002161357.1, XM_002161359.1-XM_002161405.1, XM_002161407.1-XM_002161440.1, XM_002161442.1-XM_002161495.1, XM_002161497.1-XM_002161541.1, XM_002161543.1-XM_002161571.1, XM_002161573.1-XM_002161614.1, XM_002161616.1-XM_002161633.1, XM_002161635.1-XM_002161742.1, XM_002161744.1-XM_002162017.1, XM_002162019.1-XM_002163255.1, XM_002163257.1-XM_002163587.1, XM_002163589.1-XM_002164597.1, XM_002164599.1-XM_002165037.1, XM_002165039.1-XM_002165179.1, XM_002165181.1-XM_002165206.1, XM_002165208.1-XM_002165386.1, XM_002165388.1-XM_002165426.1, XM_002165428.1-XM_002165450.1, XM_002165452.1-XM_002165555.1, XM_002165557.1-XM_002165581.1, XM_002165583.1-XM_002165658.1, XM_002165660.1-XM_002165664.1, XM_002165666.1-XM_002165741.1, XM_002165743.1-XM_002165874.1, XM_002165876.1-XM_002165937.1, XM_002165939.1-XM_002165968.1, XM_002165970.1-XM_002165993.1, XM_002165995.1-XM_002166009.1, XM_002166011.1-XM_002166023.1, XM_002166025.1-XM_002166044.1, XM_002166046.1-XM_002166072.1, XM_002166074.1-XM_002166099.1, XM_002166101.1-XM_002166227.1, XM_002166229.1-XM_002166249.1, XM_002166251.1-XM_002166268.1, XM_002166270.1-XM_002166291.1, XM_002166293.1-XM_002166400.1, XM_002166402.1-XM_002166447.1, XM_002166449.1-XM_002166477.1, XM_002166479.1-XM_002166498.1, XM_002166500.1-XM_002166520.1, XM_002166522.1-XM_002166544.1, XM_002166546.1-XM_002166566.1, XM_002166568.1-XM_002166613.1, XM_002166615.1-XM_002166665.1, XM_002166667.1-XM_002166857.1, XM_002166859.1-XM_002167064.1, XM_002167066.1-XM_002167374.1, XM_002167376.1-XM_002167520.1, XM_002167522.1-XM_002167715.1, XM_002167717.1-XM_002167771.1, XM_002167773.1-

XM_002167799.1, XM_002167801.1-XM_002167815.1, XM_002167817.1-XM_002167831.1, XM_002167833.1-XM_002167845.1, XM_002167847.1-XM_002167857.1, XM_002167859.1-XM_002167904.1, XM_002167906.1-XM_002167937.1, XM_002167939.1-XM_002168075.1, XM_002168077.1-XM_002168736.1, XM_002168738.1-XM_002168749.1, XM_002168751.1-XM_002169356.1, XM_002169358.1-XM_002169408.1, XM_002169410.1-XM_002169425.1, XM_002169427.1-XM_002169976.1, XM_002169978.1-XM_002169985.1, XM_002169987.1-XM_002169990.1, XM_002169993.1-XM_002169995.1, XM_002169997.1-XM_002170003.1, XM_002170005.1-XM_002170008.1, XM_002170010.1-XM_002170017.1, XM_002170019.1-XM_002170029.1, XM_002170031.1-XM_002170034.1, XM_002170036.1-XM_002170123.1, XM_002170125.1-XM_002170370.1, XM_002170372.1-XM_002170971.1, XM_002170973.1-XM_002171130.1, XM_002171132.1-XM_002171275.1, X70839.1, X70839.1, X67590.1, X67590.1, U53444.1, U53444.1, U36781.1, U36781.1, HQ184466.1, HQ184466.1, GU199337.1, GU199337.1, GQ983384.1, GQ983384.1, GQ856264.1, FN257513.1, FN257513.1, FJ823136.1, FJ823136.1, FJ222238.1, FJ222238.1, FJ154842.1, FJ154842.1, EU877199.1, EU877199.1, EU787490.1, EU787490.1, EU442372.1, EU442372.1, EU178740.1, EU015880.1, EU015880.1, EF370474.1, EF370474.1, EF010985.1, EF010985.1, DQ518873.1, DQ518873.1, DQ073560.1, DQ073560.1, DQ072591.1, DQ072591.1, AY841903.1, AY841903.1, AY458134.1, AY458134.1, AY422083.1, AY422083.1, AY372112.1, AY372112.1, AY332609.1, AY332609.1, AY225467.1, AY225467.1, AY216501.1, AY216501.1, AY213094.1, AY213094.1, AM233513.2, AM182483.1, AF307098.1, AF307098.1, AF188478.1, AF188478.1, AF043907.1, AF043907.1)

Fungal dataset: *Schizosaccharomyces pombe* (gi|301736437-301750575|); *Aspergillus niger* (AJ239738.1-AJ239987.1, BE758760.1-BE760957.1, CK769166.1-CK769173.1, DR697868.1-DR710686.1, EY187740.1-EY188372.1, EY223258.1-EY254202.1); *Neurospora crassa (*AA574464.1-AA574465.1, AA601776.1-AA601777.1, AA738494.1-AA738501.1, AA774383.1-AA774387.1, AA897792.1-AA899039.1, AA901496.1-AA902101.1, AA908001.1-AA908006.1, AI318697.1-AI320510.1, AI320569.1-AI322045.1, AI328149.1-AI330327.1, AI391954.1-AI391955.1, AI391957.1-AI392604.1, AI397485.1-AI399633.1, AI416404.1-AI416428.1, AW708018.1-AW719192.1, AW721859.1-AW725138.1, BE900092.1-BE900100.1, BF072409.1-BF072839.1, BF739420.1-BF739760.1, BG278041.1-BG280722.1, FK707478.1-FK707538.1, GE917356.1-GE999999.1, GH000001.1-GH158787.1); *Saccharomyces cerevisiae* (AA417440.1-AA417500.1, AA417502.1-AA417537.1, DB636784.1-DB668630.1, EG999314.1-T17502.1, T17635.1-T36312.1, T39110.1-X78018.1, EH038222.1)

Dinoflagellate dataset: *Alexandrium tamarense* (CF751845.1-CF751962.1, CF774560.1-CF774855.1, CF947047.1-CF948546.1, CK431405.1-CK433904.1, CK782344.1-CK786698.1, CV553867.1-CV555405.1), *Alexandrium catenella* (EX454357.1-EX464203.1, AB212072.1), *Alexandrium ostenteldii* (HO658038.1-HO663459.1,HO652585.1-HO658036.1), *Alexandrium mitum* (GW792032.1-GW792241.1, GW792243.1-GW792256.1, GW792258.1-GW792278.1, GW792280.1-GW792403.1, GW792405.1-GW792489.1, GW792491.1-GW792620.1, GW792634.1-GW792636.1, GW792645.1-GW792648.1, GW792652.1-GW792654.1, GW792655.1-GW792657.1, GW792662.1-GW792666.1, GW792680.1-GW792682.1, GW792706.1-GW792708.1, GW792710.1-GW792769.1, GW792771.1-GW792774.1, GW792776.1-GW792787.1, GW792789.1-GW792804.1, GW792805.1-GW792807.1, GW792820.1-GW792821.1, GW792823.1-GW792861.1, GW792863.1-GW792865.1, GW792871.1-GW792976.1, GW792980.1-GW792985.1, GW792988.1-GW793010.1, GW793012.1-GW793017.1, GW793019.1-GW793113.1, GW793115.1-GW793179.1, GW793182.1-GW793185.1, GW793187.1-GW793190.1, GW793193.1-GW793227.1, GW793229.1-GW793255.1, GW793257.1-GW793268.1, GW793270.1-GW793275.1, GW793277.1-GW793281.1, GW793283.1-GW793359.1, GW793361.1-GW793364.1, GW793366.1-GW793367.1, GW793369.1-GW793376.1, GW793411.1-GW793413.1, GW793552.1-GW793554.1, GW793755.1-GW793757.1, GW793766.1-GW793768.1, GW793832.1-GW793834.1, GW793846.1-GW793851.1, GW793853.1-GW793894.1, GW793896.1-GW793925.1, GW793927.1-GW793942.1, GW793944.1-GW793946.1, GW793952.1-GW793954.1, GW793962.1-GW793964.1, GW794043.1-GW794045.1, GW794143.1-GW794145.1, GW794188.1-GW794190.1, GW794217.1-GW794219.1, GW794223.1-GW794225.1, GW794307.1-GW794309.1, GW794319.1-GW794321.1, GW794331.1-GW794334.1, GW794357.1-GW794359.1, GW794412.1-GW794414.1, GW794415.1-GW794417.1, GW794430.1-GW794441.1, GW794443.1-GW794448.1, GW794450.1-GW794460.1, GW794462.1-GW794488.1, GW794490.1-GW794530.1, GW794532.1-GW794623.1, GW794625.1-GW794643.1, GW794645.1-GW794711.1, GW794713.1-GW794894.1, GW794896.1-GW794988.1, GW794990.1-GW795077.1, GW795079.1-GW795089.1, GW795091.1-GW795182.1, GW795184.1-GW795186.1, GW795188.1-GW795246.1, GW795248.1-GW795278.1, GW795280.1-GW795375.1, GW795377.1-GW795395.1, GW795398.1-GW795406.1, GW795410.1-GW795412.1, GW795414.1-GW795415.1, GW795417.1-GW795419.1, GW795422.1-GW795431.1, GW795434.1-GW795444.1, GW795446.1-GW795454.1, GW795458.1-GW795466.1, GW795469.1-GW795479.1, GW795500.1-GW795502.1, GW795513.1-GW795515.1, GW795520.1-GW795543.1, GW795545.1-GW795554.1, GW795557.1-GW795566.1, GW795569.1-GW795612.1, GW795614.1-GW795637.1, GW795640.1-GW795650.1, GW795652.1-GW795662.1, GW795664.1-GW795680.1, GW795682.1-GW795752.1, GW795754.1-GW795761.1, GW795763.1-GW795999.1, GW796001.1-GW796184.1, GW796186.1-GW796252.1, GW796257.1-GW796262.1, GW796264.1-GW796293.1, GW796295.1-GW796353.1, GW796355.1-GW796486.1, GW796488.1-GW796575.1, GW796608.1-GW796610.1, GW796612.1-GW796614.1, GW796616.1-GW796618.1, GW796633.1-GW796635.1, GW796656.1-GW796658.1, GW796729.1-GW796731.1, GW796752.1-GW796754.1, GW796796.1-GW796885.1, GW796573.1, GW795518.1, GW795477.1, GW794428.1, GW793940.1, GW793844.1, GW793374.1, GW792936.1, GW792940.1, GW792960.1, GW792962.1, GW792964.1, GW792883.1, GW792885.1, GW792966.1, GW792835.1, GW792839.1, GW792851.1, GW792854.1, GW792817.1, GW792794.1, GW792704.1, GW792689.1, GW792695.1, GW792676.1, GW792678.1, GW792628.1, GW792630.1, GW792618.1); *Karlodinium micrum* (EC147064.1-EC163595.1); *Karenia brevis* (CO059029.1-CO065717.1, CO517335.1-

CO517390.1, CV173737.1-CV173976.1, EX864807.1-EX878969.1, EX956452.1-EX980006.1, CV179548.1); *Symbiodinium* strain KB8 (FE537410.1-FE540062.1).

Bacterial dataset: *Escherichia coli* strain MS 175-1 (gi|EFJ63866-EFJ68735|); *Salmonella enterica* (EDZ33444-EDZ37920)

Files S2-S5

Available for download at http://www.g3journal.org/lookup/suppl/doi:10.1534/g3.113.009084/-/DC1

File S2 Expression Data Experiment 1

File S3 Expression Data Shared

File S4 Transcriptome data

File S5 Predicted species of origin of each contig

Supporting References

Anders, S., and W. Huber, 2010 Differential expression analysis for sequence count data. Genome Biol. 11: R106.

Darsigny, M., J.-P. Babeu, A.-A. Dupuis, E. E. Furth, E. G. Seidman *et al.*, 2009 Loss of hepatocyte-nuclear-factor-4α affects colonic ion transport and causes chronic inflammation resembling inflammatory bowel disease in mice. PLoS ONE 4: e7609.

Endo, Y., M. Matsushita, and T. Fujita, 2007 Role of ficolin in innate immunity and its molecular basis. Immunobiology 212: 371–379.

Ganot, P., A. Moya, V. Magnone, D. Allemand, P. Furla *et al.*, 2011 Adaptations to endosymbiosis in a cnidarian-dinoflagellate association: differential gene expression and specific gene duplications. PLoS Genet. 7: e1002187.

Gessi, S., S. Merighi, D. Fazzi, A. Stefanelli, K. Varani *et al.*, 2011 Adenosine receptor targeting in health and disease. Expert Opin. Investig. Drugs 20: 1591–1609.

Hooper, J. D., L. Campagnolo, G. Goodarzi, T. N. Truong, H. Stuhlmann *et al.*, 2003 Mouse matriptase-2: identification, characterization and comparative mRNA expression analysis with mouse hepsin in adult and embryonic tissues. Biochem. J. 373: 689–702.

Kenkel, C. D., M. R. Traylor, J. Wiedenmann, A. Salih, and M. V Matz, 2011 Fluorescence of coral larvae predicts their settlement response to crustose coralline algae and reflects stress. Proc. R. Soc. B 278: 2691–2697.

Ko, D. C., J. Binkley, A. Sidow, and M. P. Scott, 2003 The integrity of a cholesterol-binding pocket in Niemann-Pick C2 protein is necessary to control lysosome cholesterol levels. Proc. Natl. Acad. Sci. USA 100: 2518–2525.

Kobuke, K., Y. Furukawa, M. Sugai, K. Tanigaki, N. Ohashi *et al.*, 2001 ESDN, a novel neuropilin-like membrane protein cloned from vascular cells with the longest secretory signal sequence among eukaryotes, is up-regulated after vascular injury. J. Biol. Chem. 276: 34105–34114.

Lalmanach, G., C. Naudin, F. Lecaille, and H. Fritz, 2010 Kininogens: More than cysteine protease inhibitors and kinin precursors. Biochimie 92: 1568–1579.

Leggat, W., F. Seneca, K. Wasmund, L. Ukani, D. Yellowlees *et al.*, 2011 Differential responses of the coral host and their algal symbiont to thermal stress. PLoS ONE 6: e26687.

Lehnert, E. M., M. S. Burriesci, and J. R. Pringle, 2012 Developing the anemone *Aiptasia* as a tractable model for cnidarian-dinoflagellate symbiosis: the transcriptome of aposymbiotic *A. pallida*. BMC Genomics 13: 271.

Li, W.-Y., S. S. N. Chong, E. Y. Huang, and T.-L. Tuan, 2003 Plasminogen activator/plasmin system: A major player in wound healing? Wound Repair Regen. 11: 239–247.

Logan, D. D. K., A. C. LaFlamme, V. M. Weis, and S. K. Davy, 2010 Flow-cytometric characterization of the cell-surface glycans of symbiotic dinoflagellates (*Symbiodinium* spp.). J. Phycol. 46: 525–533.

Martin, F., M.-F. Penet, F. Malergue, H. Lepidi, A. Dessein *et al.*, 2004 Vanin-1(-/-) mice show decreased NSAID- and Schistosoma-induced intestinal inflammation associated with higher glutathione stores. J. Clin. Invest. 113: 591–597.

Moreau, M. E., N. Garbacki, G. Molinaro, N. J. Brown, F. Marceau *et al.*, 2005 The kallikrein-kinin system: current and future pharmacological targets. J. Pharmacol. Sci. 99: 6–38.

Rodriguez-Lanetty, M., W. S. Phillips, and V. M. Weis, 2006 Transcriptome analysis of a cnidarian-dinoflagellate mutualism reveals complex modulation of host gene expression. BMC Genomics 7: 23.

Smith, F. M., C. Vearing, M. Lackmann, H. Treutlein, J. Himanen *et al.*, 2004 Dissecting the EphA3/Ephrin-A5 interactions using a novel functional mutagenesis screen. J. Biol. Chem. 279: 9522–9531.

Sunagawa, S., E. C. Wilson, M. Thaler, M. L. Smith, C. Caruso *et al.*, 2009 Generation and analysis of transcriptomic resources for a model system on the rise: the sea anemone *Aiptasia pallida* and its dinoflagellate endosymbiont. BMC Genomics 10: 258.

Takahashi, M., D. Iwaki, K. Kanno, J. Xiong, M. Matsushita *et al.*, 2008 Mannose-binding lectin (MBL)-associated serine protease (MASP)-1 contributes to activation of the lectin complement pathway. J. Immunol. 180: 6132–6138.

Vandesompele, J., K. De Preter, F. Pattyn, B. Poppe, N. Van Roy *et al.*, 2002 Accurate normalization of real-time quantitative RT-PCR data by geometric averaging of multiple internal control genes. Genome Biol 3: RESEARCH0034.
