# Supplementary material for: Extensive Differences in Gene Expression Between Symbiotic and Aposymbiotic Cnidarians
Source: G3 (Bethesda). 2013 Dec 24;4(2):277–95. doi: 10.1534/g3.113.009084 (PMC3931562; doi:10.1534/g3.113.009084)
Supplement: Supporting Information [file supp_g3.113.009084_FigureS1.pdf]

A Full-length alignments of *Aiptasia* and other Npc2 proteins

```
Aiptasia Npc2D        MKPAISLALVVIIAAVTSIQAMKLKFKDCGSQVGE--IVSLDVTPCTSDPCSLKRGGTNA
Anemonia Npc2D        MKFLVLLLCLQIIWSLS--EARKLSFKDCGSKVGK--LVSFDLSPCSQDPCIIKR-GSNA
Human Npc2            MRFLAATFLLLALSTAA--QAEPVQFKDCGSVDGV--IKEVNVSPCPTQPCQLSK-GQSY
Drosophila Npc2A      MLRYAVIACAALVVF-----AGALEFSDCGSKTGKFTRVAIEGCDTTKAECILKR-NTTV
Aiptasia Npc2A        ---------------------------------------------------MKRK-ELKS
Anemonia Npc2A        MAKFFLIACMLYVLSLA--GAEVVDFDDCSGGKGKGEIEKLEIIPCPTQPCQLKK-GSKV

Aiptasia Npc2D        TVTINFKPHEQVTQSKIYVYAIIGIIPIPLPIPNPDACTGHGLTCPLASGKDVELVVKQS
Anemonia Npc2D        TGTVTFIPSEEVTSSKVYMYAIIGFIPVPLPLPNTDGCKGYGLTCPLKSGKPDELVFSHS
Human Npc2            SVNVTFTSNIQSKSSKAVVHGILMGVPVPFPIPEPDGCKS-GINCPIQKDKTYSYLNKLP
Drosophila Npc2A      SFSIDFALAEEATAVKTVVHGKVLGIEMPFPLANPDACVDSGLKCPLEKDESYRYTATLP
Aiptasia Npc2A        SVKRTFIPHENVTDAESSVHGKVMGFWVPFPLPNAHACKDSGVKCPLVAGSKYEYSSTLD
Anemonia Npc2A        QIKVTFVPHEDLTEATSVVHGEIGGFPVPFPLPNSNCCKDSGLTCPLKAGQKYVYTSALD

Aiptasia Npc2D        IDSTFPAGKVTVKAELKDQVQNNVLCGEVTLTLM---
Anemonia Npc2D        IDSTFPAGTVTLKGELKDQEENNIFCGKISLTLQ---
Human Npc2            VKSEYPSIKLVVEWQLQDDKNQSLFCWEIPVQIVSHL
Drosophila Npc2A      VLRSYPKVSVLVKWELQDQDGADIICVEIPAKIQ---
Aiptasia Npc2A        IKSAYPAISVVVKWQLQDGKGQDLYCFEVSAKIVS--
Anemonia Npc2A        VKSEYPAIKVVVKWEMQDKDNNDVFCFKVATQIVS--
```

B Multiple-sequence alignment used to generate Npc2 phylogenetic tree

```
A_digitifera_Npc2E              -KNCT--KNDDVTVESLDIN---PCSE-EP---CIFHK-GSTVSVTVAF-TPLEEVKSGE
A_digitifera_Npc2F              -KNCA--SRKYALPLKVAIN---PCTK-QP---CTLHP-GKKASIAVVV-KPLVTIRRGT
O_carmela_Npc2a                 -SNCTSNPGPSTLGKTVNVTAVPPCDT-AP---CVVHQ-GESLNVTVTF-VPNVAIENFT
A_digitifera_Npc2D              -QTC---DKPSGRLNSVDVT---PCNG-NP---CVFKR-GTNETITVTF-TPNEVVSKGK
Aiptasia_Npc2E                  -KDC---GSKGATIVRLDIS---PCEE-EP---CNFKT-GTTVTGTLTF-VAKEYFTSGR
N_vectensis_Npc2B               -RDC---GSQEGEIVGMDIS---PCDS-EP---CVLKR-GTSVDGSLTF-IPHEDLKRAK
Aiptasia_Npc2B                  VVVL---VVVVGIVVVVDVD---QCTSDDP---CSLKR-GTNVTSTATM-IPLEEVTQAT
Aiptasia_Npc2C                  -TDC---GSYLGEIHSLEVN---PCTS-DP---CVLKR-GDNMTSVISF-TPHEQVSAAK
Aiptasia_Npc2D                  -KDC---GSQVGEIVSLDVT---PCTS-DP---CSLKRGGTNATVTINF-KPHEQVTQSK
N_vectensis_Npc2C               -QDC---GSKKGELISVDLT---PCSS-DP---CVIKR-GANASGVITF-IPHEVVTSSK
A_viridis_Npc2D                 -KDC---GSKVGKLVSFDLS---PCSQ-DP---CIIKR-GSNATGTVTF-IPSEEVTSSK
M_faveolata_Npc2B               -ANCSDVTALEGKLISVDLT---PCPS-QP---CVFHK-GTNVTATIKF-SPEEMVTDGT
D_melanogaster_Npc2A            -SDC---GSKTGKFTRVAIE---GCDT-TK-AECILKR-NTTVSFSIDF-ALAEEATAVK
M_faveolata_Npc2A               -ADC---GSL-AKINFVDVS---PCVM-EP---CELKK-GTNESIEIQF-IPNSNITEGK
A_digitifera_Npc2B              -RDC---GNKELSPAQVIIT---PCPA-EP---CQLKK-GVNESVEVIF-KPTEVVTSAK
A_digitifera_Npc2A              -SYI---GSKESSISQVIVT---PCPA-EP---CQLKK-GVNESIEVIF-KPGEVVTSSK
Human_Npc2a                     -KDC---GSVDGVIKEVNVS---PCPT-QP---CQLSK-GQSYSVNVTF-TSNIQSKSSK
Mouse_Npc2A                     -KDC---GSKVGVIKEVNVS---PCPT-DP---CQLHK-GQSYSVNITF-TSGTQSQNST
Aiptasia_Npc2A                  ------------------------------------K-ELKSSVKRTF-IPHENVTDAE
N_vectensis_Npc2A               -KDCSG-GKGEGEIVELDIS---PCPT-QP---CTLHK-GTTVSVNITF-VPHVTLDSGK
A_viridis_Npc2A                 -DDCSG-GKGKGEIEKLEII---PCPT-QP---CQLKK-GSKVQIKVTF-VPHEDLTEAT
H_magnipapillata_Npc2D          -QNC---GHLDSNTI-VSIT---PCEK-EP---CTLVR-GSNATLEIQF-KAKHFSKQLK
H_magnipapillata_Npc2B          -KPC----DMSSTVGDVAIS---PCDK-QP---CAFQR-GGSANIEISF-TAAKDADKLT
H_magnipapillata_Npc2A          -KKCTS-PASSAVIGDVIIT---PCDS-LP---CSFKR-GGSGNIKINF-QATKNNSELT
H_magnipapillata_Npc2C          -KKCSS-PASSAVVGDVVIS---PCDN-QP---CQFIR-GGNANIQIHF-QAKKDNSNIT

A_digitifera_Npc2E              LSVDAI-AFGHRLP-M--VRKE--NICEG--HGVT-----CPLEKGKKQTFTINQKVERY
A_digitifera_Npc2F              LELYGIHWLGIKFP-LS-VPNP--DICHG--YGTR-----CPMIANSRVVLSISQTLPSF
O_carmela_Npc2A                 VVVHAS-VGIIHVP-YP-VTDP--NGCDTAVTGVT-----CPLKANVAVEWHHSFSVPSI
A_digitifera_Npc2D              ILLYAK-LVLGWIE-LS-LRNP--NICEG--YGLK-----CPLAKGVREELSVTERVPQV
Aiptasia_Npc2E                  VKAYAV-IEGVDLP-LP-IPT---DACQG--YGLT-----CPINNGQTANFVIKQEIQAD
N_vectensis_Npc2B               LSAHAI-IDKLPLP-LP-IPS---DACQG--YGLS-----CPVDSGVKSMFKIHQAIESE
Aiptasia_Npc2B                  IYMHAT-VSGITIP-ID-IPNP--NACSG--HGLS-----CPLKSGETVELSMVLEVEAK
Aiptasia_Npc2C                  IDINAI-IAGSPIH-VH-IPNP--NACDG--HGLK-----CPLEKGKKVELVVSQVIRRS
Aiptasia_Npc2D                  IYVYAI-IGIIPIP-LP-IPNP--DACTG--HGLT-----CPLASGKDVELVVKQSIDST
N_vectensis_Npc2C               VLAYAI-FGLIPVP-LP-LPNS--DGCKG--YGLT-----CPLKSGKQVELVFEHYIDQT
A_viridis_Npc2D                 VYMYAI-IGFIPVP-LP-LPNT--DGCKG--YGLT-----CPLKSGKPDELVFSHSIDST
M_faveolata_Npc2B               LQVYGF-IEGIKTP-FP-LEQP--DACKE--HGLE-----CPLKSGVTYSLEITLAIKPA
D_melanogaster_2A               TVVHGK-VLGIEMP-FP-LANP--DACVD--SGLK-----CPLEKDESYRYTATLPVLRS
M_faveolata_Npc2A               TVVYGI-IEGVQVP-FP-VDNP--EVCKE--HGIT-----CPMPAEKTQTFKATLPVKSE
A_digitifera_Npc2B              VVIHGI-IEGVRFP-FP-FPHP--NGCKE--HGLE-----CPLKPNKEYTFKATLPVKRT
A_digitifera_Npc2A              VVVHGI-IAGVPVP-FP-ISQP--NGCED--HGLD-----CPLQPNKEYTFKATLPVKSA
Human_Npc2a                     AVVHGI-LMGVPVP-FP-IPEP--DGCKS---GIN-----CPIQKDKTYSYLNKLPVKSE
Mouse_Npc2A                     ALVHGI-LEGIRVP-FP-IPEP--DGCKS---GIN-----CPIQKDKVYSYLNKLPVKNE
Aiptasia_Npc2A                  SSVHGK-VMGFWVP-FP-LPNA--HACKD--SGVK-----CPLVAGSKYEYSSTLDIKSA
N_vectensis_Npc2A               AIVHGV-IAGIPVP-FP-LPNA--DVCKN--SGLK-----CPLEPGTKYVYQSSLEVKTM
A_viridis_Npc2A                 SVVHGE-IGGFPVP-FP-LPNS--NCCKD--SGLT-----CPLKAGQKYVYTSALDVKSE
H_magnipapillata_Npc2D          TKVYGK--LLFWVPYYN-FGKE--DSCLD--NGIT-----CPVIEDEEYSYSQSLHISKL
H_magnipapillata_Npc2B          TVVKGK-IGPIWVP-FP-LSQP--DACNN--EGLT-----CPIKSSQKYTYQYSLPISES
H_magnipapillata_Npc2A          SVVKGK-IGPLWVP-FP-LSQP--DACQN--EGIT-----CPIKDGQSYLFSYDLPISTT
H_magnipapillata_Npc2C          TIVKGK-IGPLWVP-FP-LSQP--DGCLN--DGII-----CPVKTDQQYVYSYDLPISKS

A_digitifera_Npc2E              YPPLPI-DVEAYVENDNRK----ILC
A_digitifera_Npc2F              VPMGSY-QLQAVMKDQLGR--M-VLC
O_carmela_Npc2a                 APKGPVEIITWELQAPSKE--D-VAC
A_digitifera_Npc2D              LPSSTR-EVKAKLVDQNGG--T-VVC
Aiptasia_Npc2E                  FPKVKL-QLKGEVMDPQGN--M-LFC
N_vectensis_Npc2B               FPVGNL-TLKAAVTDSDTS--QVVFC
Aiptasia_Npc2B                  FPRGKV-ILKTELKDQAKN--D-IFC
Aiptasia_Npc2C                  APPGRY-RIRTELKEQYGI--D-VFC
Aiptasia_Npc2D                  FPAGKV-TVKAELKDQVQN--N-VLC
N_vectensis_Npc2C               FPTGHL-TLKAELKDQDSD--V-VIC
A_viridis_Npc2D                 FPAGTV-TLKGELKDQEEN--N-IFC
M_faveolata_Npc2B               YPSIQL-VAQMDFKLPDDG--Y-LFC
D_melanogaster_2A               YPKVSV-LVKWELQDQDGA--D-IIC
M_faveolata_Npc2A               YPALQL-DVKWELHDQDAK--V-VYC
A_digitifera_Npc2B              YQDVCM-I---RLL---------CSC
A_digitifera_Npc2A              YPDIKL-VVKWQLLDQNAN--S-VFC
Human_Npc2a                     YPSIKL-VVEWQLQDDKNQ--S-LFC
Mouse_Npc2A                     YPSIKL-VVEWKLEDDKKN--N-LFC
Aiptasia_Npc2A                  YPAISV-VVKWQLQDGKGQ--D-LYC
N_vectensis_Npc2A               YPSLKL-VVRWEIQDNKNK--D-VLC
A_viridis_Npc2A                 YPAIKV-VVKWEMQDKDNN--D-VFC
H_magnipapillata_Npc2D          NPKISI-PVKWLIQNEAEK--D-LVC
H_magnipapillata_Npc2B          YPKINL-PVSWELKDEKGE--S-LVC
H_magnipapillata_Npc2A          YPAISL-VVSWEIQDENGN--D-VVC
H_magnipapillata_Npc2C          YPAISV-VVSWELQDENGN--D-LVC
```

Figure S1 Alignments of Npc2 sequences from *Aiptasia* and other organisms. (A) Full-length alignment of selected Npc2-like proteins from *Aiptasia* sp. (this study), *A. viridis* (Ganot et al. 2011), human, and *D. melanogaster.* Red and green dots, amino acids that are identical (red) or similar (green: I,V,L; S,T; D,E; K,R; Q,N) between *Aiptasia* NpcD and human Npc2; pink shading,

amino acids whose mutation to alanine ablates the cholesterol-binding function of Npc2 in mammalian cells (Ko *et al.* 2003); blue shading, conserved cysteines used to identify conserved regions of the proteins for phylogenetic analysis; black underline, the conserved region used for phylogenetic analysis. (B, next page) The multiple-sequence alignment of the conserved regions used to produce the phylogenetic tree in Figure 2A.
