# Supplementary material for: Extensive Differences in Gene Expression Between Symbiotic and Aposymbiotic Cnidarians
Source: G3 (Bethesda). 2013 Dec 24;4(2):277–95. doi: 10.1534/g3.113.009084 (PMC3931562; doi:10.1534/g3.113.009084)
Supplement: Supporting Information [file supp_g3.113.009084_FigureS2.pdf]

A **Sequence alignment of *Aiptasia* peroxidasin-related proteins I and 2**

```
Apr2         TKTPESPMSSSKDPGKPYSCSHTKFFIVTFLSHTLLLCAGIVPLYITTNHRLVTVEDRLT 60
Apr1         ------------------------------------------------------------

Apr2        VHDMELNSCCLVKEGYGNFQDHEETKVTLNREKYVQTQFIDRVRRNTPYISRDVLNSIRM 120
Apr1        ------------------------------------------------------------

Apr2        EVRNHILNLTASQFCQVPDKICTRGAPGKRGLRGRRGSRGRRGRPGHKGIKGLPGKYGKQ 180
Apr1        ---------------------RQRGPPGPPGLPGKSGPRGSIGPQGPK------------ 27
                         Box I → **.**  ** *: *.**  *  * *

Apr2        GLRGFPGQKGQKGDIGNRGPPGLPGPKGERGKEVTEPSVFISPSILTVTENQTATFHCNA 240
Apr1        ------GLAGKKGDIGRPGLPCKPLIIN------YPPKVSLPVGPIYVKEGDNLILSCHV 75
                  *  *:*****. * *  *   .Box 2 → *.* :. . : *.*.:.  : *:.

Apr2        HGYPKPQITWKMGSGKQIDFGKTRIDKSAGLLEISNVSEKDTGNYTCSAKSVLGEDANTV 300
Apr1        TGYPKPKVTWSKVMDS---LPSKRSFITTNRLKVLSVQKQDSGLYVCAGSNTLGSAVETI 132
             *****::**.   ..  : ..*   ::. *:: .*.::*:* *.*:....**. .:*:

Apr2        SLLVKFPPRFTEVQKPFQTILQGSTVNLKCAALGYPPPIITWTKMLGSLPTKRSQQNGGK 360
Apr1        KVIVVSAPKFISTPPQQVNKNTCEKLTLDCQARGDPPAVITWSKEKGRLPTDRTQLINGR 192
            .::*  .*:* ..    .    ..:.*.* * * **.:***:*  * ***.*:*  .*:

Apr2        LTITRFQQSDSGSYQCEAVNSVGKNIFYTTLSFGACDDALGMQSKAIRDSQITASSSYSS 420
Apr1        LTITGMTTSDAGKYTCTAVSAGVATSKSVTRVTVKETGKLKFSRDSVTDYIVVKR----- 247
            **** :  **:*.* * **.:    .   .* ← . * :. .:: *  :.
                                              Box 3

Apr2        AYLPYYGRLNIVLGYGGWLAKSNTKGQWIQVDLLQATRITAIATQGTSKYDEWTTSYSLQ 480
Apr1        -KLPAMARLTVCL----WMMTSKKNSVLISYAVPGSINEILLLDVG-KRLSVWLGDVSWD 301
              **  .**.: *    *: .*:.:.  *.  :  : .   :   * .: . *  . * :

Apr2        YSYDGTSFRDYEGGKTLPGNSDRSTVVKNNLDPAIAARYIRLLPKTYHSYMVIRMELYGC 540
Apr1        SGVHVTDGQWHHICATWDNSAGQTILYKDGVRRAPSSTRSR------------------- 342
             . .*. : :.   *  ..:.:: : *:.:  * ::   *

Apr2        QL 542
Apr1        --
```

B **Comparison of protein domains found in human peroxidasins and the *Aiptasia* peroxidasin-related proteins**

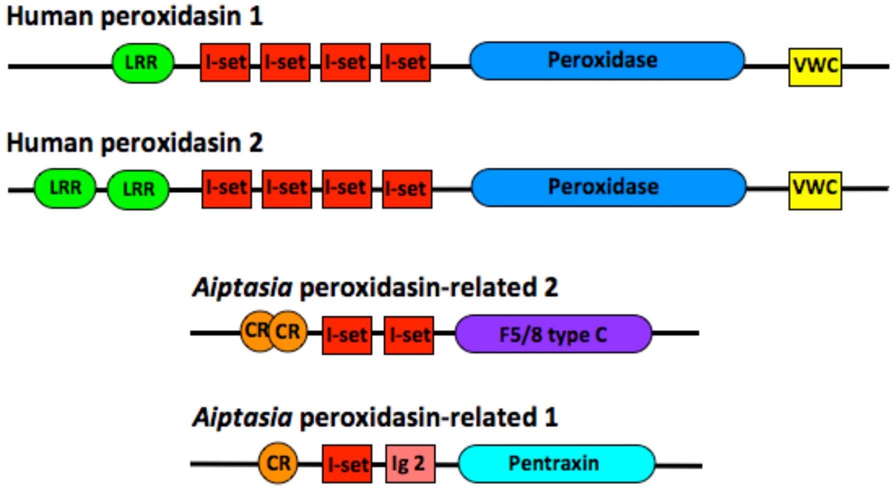

C Sequence alignment of *Aiptasia* plasma-kallikrein homologs

```
73922      ---------------------------------------------LRSTPTIGIDIKALHF 16
84752      -------------------------------------------------LTG------DV 5
12789      TGDCSPGGSGYYMYAEASSPRRDMDVARLETSQKFSFDGGNKCLKFMYHMYGKDMGFMDV 120
                                                              *      ..

73922      VSNIRLEATTKKLKMTLG------------------------SLFIILTVASCVILRSSA 52
84752      VVS--VVVWTAGVSIG------------------------------SSFDVSCVSFASVS 33
12789      YVGNDLVWWRGGGSMGNTWHSANIPVLISSGSYKIAFEAYRGYGYRSDIAIDEISLEDCT 180
             .  :       .:                                    . : : . :

73922      EES-------------------------------------VNATLGDIEDKVDMIETRV 74
84752      LVG---------------------------------------HVVEGSCGYKP---NVRI 51
12789      TATPQPPTQPPATQPPPPPSTQAPTPPPPTQAPTPPPPNTPPPPPSGSCGIRP---LTRI 237
                                                         *.   :     .*:

73922      IKGQNANLHEWPWQVSLEYNG---HHICGGSLVKRDWVLTAAHCFNRFRYVPSWRVVVGR 131
84752      VGGTEAPPGAWPWQVMIRTKSSFPGHFCGGSLIHPQWVVTAAHCLYGRTEN-DFFVRLGA 110
12789      VGGTDANHGDWPWQALLRYAS--GSQFCGGALVAPQWVVSASHCVSSLSAS-DIHIRMGA 294
           : * :*    ****. :.  .    ::***:*:  :**::*:**.       .  : :*

73922      HTIKGVTQFERRYQVRRIITHAYYHKTR-FINDVALMQLQASVVLSKQVNVITLPTHNNR 190
84752      HYRDSVMGTEQDIDIEKMIKHWSYGKPKSLSHDIALLKLKRPVRLSKDAGLVCLPRTYSN 170
12789      HKRTSSVGTEQDFKVIKKIMHESYQNPKQYSNDIALLKLEKPVTLDKYTNLVCLPPRAVD 354
           *   .    *:  .: : * *  *  :.:   :*:**::*: .* *.* ..:: **

73922      VAAGSVCYITGWGLTHGLIKESAAHVLQEAPLTIAAFWHCSAVNNQLNIEIDEKTMVCAG 250
84752      VLQGTKCWVTGWGKLS--AGGADPRVLMQVSVPIVSRRKCQNS----YPGQIDDSMICAG 224
12789      IATDSKCWITGWGTLS--SGGSQPETLQQAEVPIVSPATCQNS----YPNMIDKTMVCAG 408
           :  .: *::****        : ...* :. :.*.:   *.           :.:*:***

73922      G--GGKGGCRGDSGGPLACNERGHWILRGVVSWGHAKCSAEEFYTVFARVSSFVDWIKKI 308
84752      YDQGGRDSCQNDSGGPLVCEQFGRFYLEGVVSWGEG-CAGRLKYGVYANVRYLKRWIYNN 283
12789      LKKGGVDACQGDSGGPMVCESNGAFYLHGATSWGYG-CAAPDKYGVYARVSHLRDWIDQK 467
              ** ..*:.*****:.*:. * : *.*..*** . *:.   * *:*.*  :  ** :

73922      TQQYAAKDGGCGGRSGIVCLNGGTQFCYNKFPTCRCRLGFSGKNCQTGPTNTVNRPSHAN 368
84752      MR---------------------------------------------------------- 285
12789      IASN-------------------------------------------------------- 471
```

Figure S2 Distinct but related genes whose products may be involved in host tolerance of the symbiont. The transcripts differentially expressed between symbiotic and aposymbiotic anemones included two whose top blastx hit in SwissProt was a human peroxidasin and three whose top blastx hit was a mammalian plasma kallikrein (Figure 6; Table S6). (A,B) The two *Aiptasia* peroxidasin-related proteins (Apr1 and Apr2) appear to represent distinct gene products with limited domain homology both to each other and to human peroxidasins. (A) ClustalW sequence alignment of the two *Aiptasia* proteins shows interspersed identical and different amino acids as expected from distinct gene products rather than from alternative splice products or misassembled contigs. Boxes show regions of sequence similarity between the *Aiptasia* proteins but not the human ones (Box 1) or among all four proteins (Boxes 2 and 3), as diagrammed in B. *, :, and . indicate identical, conserved, and semi-conserved amino acids, respectively. (B) Schematic diagram comparing protein domains found in human peroxidasins and the *Aiptasia* peroxidasin-related proteins using Pfam. LRR, leucine-rich repeat; I-set, immunoglobulin I-set; Peroxidase, domain with similarity to canonical peroxidases; VWC, von Willebrand factor type-C; CR, collagen triple-helix repeat; Ig 2, immunoglobulin; F5/8 type C domain (or discoidin domain), with cell-adhesion functions; Pentraxin, domain with similarity to pentraxin pattern-recognition receptors displaying Ca^{2+}-dependent ligand binding. (C) ClustalW sequence alignment of three *Aiptasia* plasma-kallikrein homologues shows interspersed identical and different amino acids as expected from distinct gene products. The box shows the region of sequence similarity representing the shared trypsin-like domain. Symbols are as in A.
