# Supplementary material for: Extensive Differences in Gene Expression Between Symbiotic and Aposymbiotic Cnidarians
Source: G3 (Bethesda). 2013 Dec 24;4(2):277–95. doi: 10.1534/g3.113.009084 (PMC3931562; doi:10.1534/g3.113.009084)
Supplement: Supporting Information [file supp_g3.113.009084_TableS1.pdf]

Table S1 Correlation between RNA-Seq and RT-qPCR measurements of differential gene expression in symbiotic relative to aposymbiotic anemones.[a]

Locus #/ transcript #	Top Blast Hit	UniProt accession number	Read count [b]	Fold-change (RNA-Seq)	Fold-change (RT-qPCR)
58798/1	Bovine Na^+- and Cl^--dependent taurine transporter	Q9MZ34	61	∞	29
102514/1	Human Npc2 cholesterol transporter	P61916	269	1197	26
95010/1	Mouse tumor necrosis factor receptor superfamily member 27	Q8BX35	202	240	33
125065/1	*Drosophila* organic-cation (carnitine) transporter	Q9VCA2	255	131	57
77179/1	Human scavenger receptor class B member 1 (SRB1; CD36-related)	Q8WTV0	11	28	3.7
95925/1	*Bacteroides thetaiotaomicron* glutamate dehydrogenase	P94598	852	13	2.9
86800/1	Human facilitated glucose transporter (GLUT8)	Q9NY64	57	12	6.3
65589/1	Sheep aquaporin-5	Q866S3	71	11	2.2
70728/1	*C. elegans* NH_4^+ transporter 1 (AMT1-type)	P54145	1382	6.4	7.0
95114/1	Mouse aromatic-amino-acid transporter 1	Q3U9N9	54	5.9	6.2
101012/1	*Bacillus halodurans* isocitrate lyase	Q9K9H0	79	3.9	4.6
66644/1	Human carnitine O-palmitoyltransferase 1	P50416	1237	2.4	2.8
101000/1	*S. cerevisiae* delta(24(24(1)))-sterol reductase	P25340	40	2.0	∞
105631/1	Rat Na^+- and Cl^-dependent GABA transporter 1	P23978	1302	2.0	1.9
125822/1	*Cerberus rynchops* ficolin (collagen/fibrinogen domain containing lectin) 2	D8VNS9	187	1.7	1.8
27493/1	*Salmo salar* Golgi pH regulator	B5X1G3	61	1.2	1.3
12296/1	60*S* ribosomal protein L11	P46222	280	1.1	0.9
119098/1	Rat 40*S* ribosomal protein s7	Q9ZNS1	94	1.0	1.1
12335/1	*Dictyostelium* F-box/WD repeat-containing protein A-like protein	Q54N86	239	-1.0	-1.3
84201/1	*Metridium senile* cytochrome c oxidase	Q35101	1784	-1.4	-1.4
58671/1	*Coturnix japonica* glyceraldehyde-3-phosphate dehydrogenase	Q05025	237	-1.4	-1.1
77428/1	Superoxide dismutase	P81926	987	-1.6	-1.7
21845/2	Rat apoptosis-inducing factor mitochondrial	Q9JM53	769	-1.6	-1.1
59465/1	Rat calmodulin-like protein 3	Q5U206	679	-1.6	-1.5
13527/1	Rat monocarboxylate transporter 10	Q91Y77	47	-1.7	-1.8
12461/1	Rat mannan-binding lectin serine protease 1	Q8CHN8	223	-3.1	-2.8
431/2	Human Na^+/glucose cotransporter 4	Q2M3M2	67	-3.2	-2.0
1568/1	Mouse E2F transcription factor 2	P56931	136	-3.5	-2.1
20440/1	Zebrafish delta-like protein c	Q9IAT6	2	-∞	-1.8

[a] Transcripts are arranged (top to bottom) in order of their degree of expression in symbiotic relative to aposymbiotic anemones as determined by RNA-Seq. Only the data from RNA-Seq Experiment 1 are used, because its conditions matched more closely those of the RT-qPCR experiment (see Materials and Methods and Table 1).

[b] The baseMean expression value as calculated by DESeq (Anders and Huber 2010).
