# Supplementary material for: Extensive Differences in Gene Expression Between Symbiotic and Aposymbiotic Cnidarians
Source: G3 (Bethesda). 2013 Dec 24;4(2):277–95. doi: 10.1534/g3.113.009084 (PMC3931562; doi:10.1534/g3.113.009084)
Supplement: Supporting Information [file supp_g3.113.009084_TableS2.pdf]

Table S2 Primer sequences and product sizes for RT-qPCR data. [a]

Locus #/ transcript #	Forward Primer	Reverse Primer	Product Size
58798/1	AAAGATCTGCTGGCTGACCCTGA	AACACCAACCAATTGCCTCACCC	134
102514/1	AAGTGACCCGTGCGTTCTCAAA	TGCGTTTGGGTTGGGAATGTGT	148
95010/1	TTTGACATGCTGCGCGAACTGCT	AATGGCCACGACGTGTTTGAAGG	225
125065/1	TGTCAGTGGCGTTGCACAGTCTT	ACATTGCCAATTCTTGCGCGGT	159
77179/1	GAAATGGCGGAAAAAGCATA	GGTGGAAATTGTGTCCCATC	225
95925/1	CAAAGCCTGGACATCGACGCAAA	CAATGACACAGGCCCGCAGAAA	194
86800/1	AGCTGGAGGGAAGGCACCAATAA	TGGGAGCTGTCAATCAACTTGGGA	110
65589/1	TTTGCCGGGAACACGTGCATT	TGAGCGCCGAGTGATGTAGGA	177
70728/1	ACCAACGGATTCCCATTCTCGTCA	TTTGCGGGCAGCAGTGTTGTT	110
95114/1	TGTCGCGCTGTTGCCTTTGTT	TGGCCAAAGCAAGGCGTTTGTGA	187
101012/1	GGTCAGCACGCATGAAAGCATTGT	AAGCAATCCAGATGGCAAAGGCAG	171
66644/1	TCCAAGACCAAGTGTTGGTGGACT	TGATCCAAGTCAGGGACAGGCAAA	110
101000/1	TCTGTCGTGGACACTGCTGTTGA	ATCCAACCGAACTTCTCCGTGGT	189
105631/1	ACCGTGAACACTTCTTGAGAGCCA	GCCTCGGTTGAATGCTTTGTTCGT	210
125822/1	ACCTCGCGCCTTGTCCTTATCAAA	AATGGGACTGTTAAGGCGGTTCGT	225
27493/1	GGTTTGCTGCATCTTCACAGGTCA	AGAAACAGCTGGCGACTAAGCTCT	133
12296/1	AGCCAAGGTCTTGGAGCAGCTTA	TTGGGCCTCTGACAGTACAGTGAACA	125
119098/1	ACTGCAGTCCACGATGCTATCCTT	GTCTGTTGTGCTTTGTCGAGATGC	125
12335/1	TGAAACCTCCTTTCAGCCTCCCA	TCACTTCACTCATCTCGGCAGCA	172
84201/1	AGCAGTTGGTAAGTCTGCACAA	GTAACCATGGTAGCAGCATGAA	105
58671/1	AACAGCTTTGGCAGCACCTGTAGA	TGCTTTCACAGCAACCCAGAAGAC	114
77428/1	AAGGCAAGCGGTAACGAGGTTT	TGCTTTCCTTCTGTCAGCCCAGT	177
21845/2	TCATGGCAAGGACGACGAGTGAA	TCACCCATGGCAGTAAAGAGCGA	156
59465/1	TCGGCAGGATTGTGTCCAAGTGA	AAACGAGCGACACAACGTCAGCA	197
13527/1	AGACACCCAACTGTTCCTTCCCA	ACACGCCGTAAGTAAACGCCAA	212
12461/1	AGCAAAGGGCACGAACAACCAAC	TTGACTCGCTATGGCCGCTAACA	125
431/2	TGGCCTTCAACAAACCTTCACGCT	ACGTTTGTAGTCCCAGCCAGTCA	238
1568/1	AAGTTCGTTGGAGGGTACTGCGA	CCACCAAAGACTTCACACAGCCA	110
20440/1	AATGGCGGAGTTTGTCAAGACGG	TGCCGATGCATTTGCCTGAGTT	118

[a] Transcripts are listed in the same order as in Table S1.
