# Supplementary material for: Extensive Differences in Gene Expression Between Symbiotic and Aposymbiotic Cnidarians
Source: G3 (Bethesda). 2013 Dec 24;4(2):277–95. doi: 10.1534/g3.113.009084 (PMC3931562; doi:10.1534/g3.113.009084)
Supplement: Supporting Information [file supp_g3.113.009084_TableS3.pdf]

Table S3 Transport-related genes showing differential expression in symbiotic relative to aposymbiotic anemones. [a]

Line	Fold-change [b]	Read count[c]	Locus#/ transcript#	Best BLAST hit	UniProt accession number	BLAST-hit E-value
1	∞	78	58798/1	Bovine Na^+- and Cl^--dependent taurine transporter	Q9MZ34	1e-169
2	∞	81	36456/1	Rabbit Na^+/(glucose/*myo*-inositol) transporter 2	Q28728	3e-104
3	600	659	102514/1	Human Npc2 cholesterol transporter	P61916	2e-14
4	131	437	60777/1	Zebrafish NH_4^+ transporter rh type b	Q7T070	3e-98
5	44	150	125065/1	*Drosophila* organic-cation (carnitine) transporter	Q9VCA2	6e-35
6	28	11	77179/1	Human scavenger receptor class B member 1 (SRB1; CD36-related)	Q8WTV0	9e-65
7	13	70	65589/1	Sheep aquaporin-5	Q866S3	8e-37
8	11	52	86800/1	Human facilitated glucose transporter (GLUT8)	Q9NY64	9e-89
9	6.9	94	12006/1	*Xenopus* GABA and glycine transporter	Q6PF45	8e-60
10	5.9	881	70728/1	*C. elegans* NH_4^+ transporter 1 (AMT1-type)	P54145	6e-72
11	5.8	1667	45451/1	*Drosophila* lipid-droplet surface-binding protein 2	Q9VXY7	2e-08
12	4.9	45	95114/1	Mouse aromatic-amino-acid transporter 1	Q3U9N9	3e-65
13	4.3	198	84722/1	Fish (*Tribolodon*) carbonic anhydrase II	Q8UWA5	2e-36
14	4.3	288	2130/2	Pig aquaporin-3	A9Y006	1e-68
15	3.7	111	11708/1	Human facilitated glucose transporter (GLUT8)	Q9NY64	1e-88
16	3.6	2547	101327/1	Rat neutral- and basic-amino-acid transporter 1	P82252	2e-117
17	3.5	52	103419/1	Chicken monocarboxylate transporter 4 (slc16a3)	P57788	1e-28
18	3.1	71	37788/1	Rabbit hyperpolarization-activated cation channel 4	Q9TV66	9e-129
19	3.1	707	56440/1	Mouse Na^+-independent SO_4^- transporter	Q80ZD3	1e-126
20	2.9	241	97639/1	Bovine ABC subfamily f member 2	Q2KJA2	0
21	2.9	264	11677/1	*Arabidopsis* ABC transporter g family member 27	Q9FT51	7e-67
22	2.7	35	26261/3	*Dictyostelium* UDP-sugar transporter	Q54YK1	1e-32
23	2.6	108	49092/1	Mouse zinc transporter 1 (znt-type)	Q60738	4e-70

24	2.4	61	15916/1	Human major-facilitator-superfamily-domain-containing protein 12	Q6NUT3	9e-37
25	2.4	991	66644/1	Human carnitine *O*-palmitoyltransferase 1	P50416	0
26	2.2	35	119860/1	Mouse Na^{+}/(glucose/*myo*-inositol) cotransporter 2	Q8K0E3	4e-125
27	2.1	3879	76979/1	Human Na^{+}-dependent phosphate-transport protein 2b	O95436	2e-112
28	2.1	4804	109479/1	Human neutral- and basic-amino-acid transport protein	Q07837	1e-49
29	2.1	554	12947/1	Rat very-low-density-lipoprotein receptor	P98166	4e-162
30	2.1	53	37499/1	Human aromatic-amino-acid transporter 1	Q8TF71	6e-49
31	2.1	714	14877/3	Zebrafish pyrimidine-nucleotide carrier	Q6DG32	1e-69
32	2.1	243	16360/1	*Xenopus* monocarboxylate transporter 12 (slc16a12)	Q6P2X9	3e-35
33	2.0	88	22123/1	Rat chloride channel clic-like protein	Q9WU61	1e-16
34	2.0	61	120787/1	Mouse aromatic amino acid transporter 1	Q3U9N9	7e-34
35	2.0	989	105631/1	Rat Na^{+}- and Cl^{-}-dependent GABA transporter 1	P23978	9e-124
36	2.0	231	2338/1	Bovine zinc transporter (zip-type)	A5D7L5	1e-43
37	2.0	582	49156/1	Human ABC subfamily b member 1	P08183	3e-108
38	1.9	1076	86906/1	Human lipid-transfer protein	Q9NQZ5	1e-52
39	1.8	218	120269/1	Mouse Na^{+}-dependent neutral-amino-acid transporter	O88576	2e-117
40	1.8	1272	36717/5	Rat neutral- and basic-amino-acid transporter 1	P82252	2e-116
41	1.8	381	19286/1	Rat v-ATPase subunit f	P50408	3e-41
42	1.8	211	81279/1	Rat Na^{+}-dependent phosphate transporter 1	Q9JJP0	2e-71
43	1.7	105	82158/1	*Columba livia* carnitine *O*-acetyltransferase	P52826	5e-151
44	1.7	176	12043/1	Mouse carnitine *O*-palmitoyltransferase 2	P52825	0
45	1.7	1020	70022/1	Rat neutral- and basic-amino-acid transporter 1	P82252	2e-128
46	1.7	193	126133/1	Zebrafish zinc transporter (znt-type)	Q5PQZ3	2e-112
47	1.6	1058	33916/1	Human transitional-ER ATPase	P55072	0
48	1.6	479	86475/1	*Xenopus* peptide transporter 4	Q68F72	1e-98
49	-1.6	164	22834/1	Chicken monocarboxylate transporter 3	Q90632	2e-30
50	-1.6	684	108875/1	Chicken low-density-lipoprotein receptor-	P98157	0

				related protein 1		
51	-1.6	404	28605/1	Rat TRP cation-channel subfamily a, member 1	Q6RI86	1e-115
52	-1.8	1323	33284/1	Mouse aromatic amino acid transporter 1	Q3U9N9	3e-34
53	-1.8	5989	71915/1	Rabbit non-specific lipid-transfer protein	O62742	0
54	-1.8	817	16745/1	Rat plasma membrane Ca^{2+-}transporting ATPase	P11505	3e-98
55	-2.0	39	13527/1	Rat monocarboxylate transporter 10 (aromatic amino acid transporter 1)	Q91Y77	6e-19
56	-2.1	33	122320/1	Human long-chain fatty acid transport protein 1	Q6PCB7	3e-96
57	-2.2	848	43841/1	Chicken ovotransferrin	P02789	3e-47
58	-2.3	4512	98994/1 [d]	Human Npc2 cholesterol transporter	P61916	5e-09
59	-2.4	5556	44110/1	Human low-density lipoprotein receptor-related protein 4	O75096	8e-61
60	-2.8	40	76106/1	*C. elegans* TRP-like cation channel protein 1	P34586	3e-55
61	-2.9	190	93152/1	Chimpanzee NH_4^+ transporter rh type c	Q3BCQ7	5e-111
62	-2.9	629	104248/1	Rat serotransferrin	P12346	2e-47
63	-3.0	506	76019/1	Carbonic anhydrase	P83299	1e-37
64	-3.5	1893	56973/1	Mouse organic cation carnitine transporter 3	Q9WTN6	5e-31
65	-3.5	301	432/1	Human Na^+/glucose cotransporter 4	Q2M3M2	2e-130
66	-3.8	325	129624/1	*E. coli* high-affinity choline-transport protein	P0ABD0	2e-78

[a] Putative small-molecule transporters and some proteins of related function (see text) are arranged in order of their degree of differential expression in symbiotic anemones relative to aposymbiotic anemones. Positive fold-changes, expression higher in symbiotic anemones; negative fold-changes, expression higher in aposymbiotic anemones.

[b] The arithmetic mean of the values from the two RNA-Seq experiments, except in line 6 (transcript 77179/1). ∞, expression was not detected in aposymbiotic animals. Transcript 77179/1 was not detected in aposymbiotic anemones in Experiment 2, giving a nominal ∞-fold change in expression. However, as the normalized read counts in both experiments were rather low, and the possible involvement of the 77179/1-encoded protein in lipid metabolism makes it likely to have been affected in its expression by the starvation conditions used in Experiment 2, we report in line 6 the more conservative value from Experiment 1 alone.

[c] Except for line 6, the average of the baseMean expression values (as calculated by DESeq (Anders and Huber 2010)) for Experiment 1 and Experiment 2. As explained in footnote b, for transcript 77179/1 (line 6), we show the value for Experiment 1 alone.

[d] Appears to represent a truncated version of transcript 98999/1, whose predicted protein product was used for the phylogenetic analysis of Figure 2A.
