# Supplementary material for: Extensive Differences in Gene Expression Between Symbiotic and Aposymbiotic Cnidarians
Source: G3 (Bethesda). 2013 Dec 24;4(2):277–95. doi: 10.1534/g3.113.009084 (PMC3931562; doi:10.1534/g3.113.009084)
Supplement: Supporting Information [file supp_g3.113.009084_TableS4.pdf]

Table S4 Lipid-metabolism genes showing differential expression in symbiotic relative to aposymbiotic anemones. [a]

Line	Metabolic Process	Putative protein function (best BLAST hit)	UniProt accession number	BLAST-hit E-value	Locus #/ transcript #	Fold-change [b]
1	FA synthesis	ACC1: Acetyl-CoA carboxylase (chicken)	P11029	0	26166/1	3.9
2	FA synthesis	ELOVL4: Elongation-of-very-long-chain-fatty-acid protein 4 (mouse)	Q9EQC4	1e-23	4012/1	4.2
3	FA synthesis	Δ^5 fatty-acid desaturase (*Mortierella alpina*)	O74212	6e-39	120701/1	6.2
4	FA synthesis	Δ^6 fatty acid desaturase (human)	O95684	6e-46	92492/1	3.5
5	Lipid storage	DHAPAT: dihydroxyacetone phosphate acyltransferase (human)	O15228	1e-95	8091/1	1.4
6	Lipid storage	2-acylglycerol *O*-acyltransferase 2-a (*Xenopus*)	Q2KHS5	8e-75	10118/2	2.7
7	Lipid storage	2-acylglycerol *O*-acyltransferase 2-a (*Xenopus*)	Q2KHS5	4e-89	15365/1	-2.0
8	Lipid storage	2-acylglycerol *O*-acyltransferase 2-b (*Xenopus*)	Q5M7F4	9e-81	78512/1	2.4
9	Lipid storage	Diacyglycerol *O*-acyltransferase (*Mycobacterium tuberculosis*)	O06795	1e-19	76581/1	2.1
10	Lipid storage	AGPAT 1: 1-acyl-*sn*-glycerol-3-phosphate acyltransferase alpha (human)	Q99943	2e-23	67491/1	-1.4
11	Lipid storage regulation	Lipid-droplet surface-binding protein 2 (*Drosophila*)	Q9VX7	2e-08	45451/1	5.8
12	Lipase	HSL: Hormone-sensitive lipase (human)	Q05469	2e-84	13988/1	1.8
13	Lipase	ATGL: Adipose triglyceride lipase (mouse)	Q8BJ56	9e-62	16411/1	-3.3
14	FA transport	FATP1: Fatty-acid transport protein 1 (human)	Q6PCB7	3e-96	122320/1	-1.9
15	FA transport	FATP4: Long-fatty-acid transport protein 4 (orangutan)	Q5RDY4	8e-92	122313/8	-3.1
16	FA transport	SRB1: Scavenger receptor class B member 1 (human; CD36-related protein)	Q8WTV0	9e-65	77179/1	28
17	FA-CoA-ligase	ACSL4: Long-chain-fatty-acid ligase 4 (human)	O60488	0	89704/1	5.7
18	FA-CoA-ligase	ACSL5: Long-chain-fatty-acid ligase 5 (human)	Q9ULC5	5e-116	106694/1	2.9
19	FA β-oxidation	Organic cation (carnitine) transporter (*Drosophila*)	Q9VCA2	6e-35	125065/1	44
20	FA β-oxidation	CPT1: Carnitine *O*-palmitoyltransferase 1 (human)	P50416	0	66644/1	2.4
21	FA β-oxidation	CPT2: Carnitine *O*-palmitoyltransferase 2 (mouse)	P52825	0	12043/1	1.6
22	FA β-oxidation	CACT: Carnitine acylcarnitine carrier protein (bovine)	Q08DK7	2e-60	13918/1	n.s.
23	FA β-oxidation	VLCAD: Very-long-chain-specific acyl-CoA dehydrogenase (bovine)	P48818	0	108541/1	1.6
24	FA β-oxidation	MTP: Trifunctional enzyme (pig)	Q29554	0	33057/3	1.4
25	FA β-oxidation	MCAD: Medium-chain-specific acyl-CoA dehydrogenase (bovine)	Q3SZB4	5e-165	92556/1	n.s.
26	FA β-oxidation	SCAD: Short-branched-chain-specific acyl-CoA dehydrogenase (*Dictyostelium*)	Q54RR5	2e-121	127382/1	n.s.
27	FA β-oxidation	Crotonase: Short-chain enoyl-CoA hydratase (*Dictyostelium*)	Q1ZXF1	4e-67	117452/1	n.s.
28	FA β-oxidation	M/SCHAD: Medium and short-chain l-3-	Q16836	5e-29	34949/1	n.s.

		hydroxyacyl-CoA dehydrogenase (human)				
29	FA β-oxidation	DCI: Enoyl-Δ isomerase (human)	P42126	7e-33	55782/1	n.s.
30	FA β-oxidation	MCKAT: 3-ketoacyl-CoA thiolase (rat)	P13437	5e-145	56206/1	2.4
31	Glyoxylate cycle	Isocitrate lyase (*Bacillus halodurans*)	Q9K9H0	3e-164	101012/1	3.9
32	Glyoxylate cycle	Malate synthase (*Myxococcus xanthus*)	P95329	2e-141	22622/1	n.s.

[a] Genes encoding proteins putatively involved in lipid metabolism are arranged in groups by biological process (see Figure 3). FA, fatty acid.

[b] Because of the likelihood that the starvation conditions in Experiment 2 would affect lipid metabolism, the fold-change values from Experiment 1 are shown. Positive fold-changes, expression higher in symbiotic anemones; negative fold-changes, expression higher in aposymbiotic anemones. n.s., no significant differential expression observed.
