# Supplementary material for: Extensive Differences in Gene Expression Between Symbiotic and Aposymbiotic Cnidarians
Source: G3 (Bethesda). 2013 Dec 24;4(2):277–95. doi: 10.1534/g3.113.009084 (PMC3931562; doi:10.1534/g3.113.009084)
Supplement: Supporting Information [file supp_g3.113.009084_TableS5.pdf]

Table S5 Presence or absence in the *Aiptasia* transcriptome of genes encoding the enzymes involved in the synthesis of particular amino acids. [a]

Line	Amino acid	Enzyme	UniProt accession number	*Aiptasia* locus #/transcript #
1	Gln	Glutamine synthetase	P32288	104234/1
2	Glu	Glutamate synthase	Q12680	60857/1
3	Glu/Pro	NADP-specific glutamate dehydrogenase [b]	Q9C8I0	3911/1
4	Glu/Pro	NADP-specific glutamate dehydrogenase 2 [b]	P39708	95925/1
5	Glu/Pro	NAD-specific glutamate dehydrogenase [c]	P33327	99746/1
6	Glu/Pro	Glutamate dehydrogenase 2 [c]	Q38946	7229/1
7	Met	MTHFR: Methylenetetrahydrofolate reductase	Q9WU20	15095/1
8	Met	MS: Methionine synthase (cobalamin-dependent)	Q99707	50131/1
9	Met	Methionine synthase (cobalamin-independent) [d]	P05694	55393/1
10	Met	BHMT: Betaine-homocysteine *S*-methyltransferase 1	Q93088	45257/1
11	Cys/SAM [e]	MAT: Methionine adenosyltransferase 1	Q91X83	140402/1
12	Cys	SAHH: *S*-adenosyl-L-homocysteine hydrolase	P27604	91092/1
13	Cys	CBS: Cystathionine β-synthase	P32582	98284/1
14	Cys	CGL: Cystathionine γ-lyase	P31373	7792/1
15	Met/Cys/Thr/Ile/ Lys	Aspartokinase/homoserine dehydrogenase	Q9SA18	
16	Met/Cys/Thr/ Ile/Lys	Aspartokinase	P10869	
17	Met/Cys/Thr/ Ile	Homoserine dehydrogenase	P31116	
18	Met/Cys	HAT: Homoserine *O*-acetyltransferase	P08465	70690/1
19	Met/Cys	CGL: Cystathionine γ-synthase	P47164	974/1
20	Met	Cystathionine β-lyase	P43623	
21	Met	Homocysteine *S*-methyltransferase 3	Q8LAX0	4396/6
22	Ser	D-3-phosphoglycerate dehydrogenase 1	P40054	294/1
23	Ser	Phosphoserine aminotransferase	P33330	57256/1
24	Ser	Phosphoserine phosphatase	P42941	122485/1
25	Ser	Catabolic L-serine/threonine dehydratase	P25379	21690/1
26	Ser/Gly	Serine hydroxymethyltransferase, mitochondrial	P37292	11787/1
27	Ser/Gly	Serine hydroxymethyltransferase, cytosolic	P37291	11787/1
28	Gly	Alanine-glyoxylate aminotransferase 1	P43567	47533/1
29	Gly	Serine-glyoxylate aminotransferase	Q56YA5	47531/1
30	Gly	Low specificity L-threonine aldolase	P37303	109186/1
31	Asp/Glu/Asn	Aspartate aminotransferase, mitochondrial	Q01802	23248/1
32	Asp/Glu/Asn	Aspartate aminotransferase, cytoplasmic	P46646	111366/1
33	Asn	Asparagine synthetase	P49089	51175/1
34	Ala	Alanine aminotransferase 1	P52893	89107/1
35	Pro	γ-glutamyl phosphate reductase	P54885	128220/1
36	Pro	Pyrroline-5-carboxylate reductase	P32263	115939/1
37	Arg	Caramoyl-phosphate synthetase	P31327	21357/1
38	Arg	Ornithine carbamoyltransferase	P00480	116500/1
39	Arg	Argininosuccinate synthetase	P22768	53174/1
40	Arg	Argininosuccinate lyase	P04076	29236/1
41	Arg	Arginase-1	P05089	118787/1
42	Arg	*N*-acetylglutamate synthase	Q8N159	19094/1
43	Arg	Acetylglutamate kinase	Q01217	
44	Arg	Ornithine acetyltransferase	Q04728	

45	Phe/Tyr	Aromatic/aminoadipate aminotransferase 1	P53090	109224/1
46	Tyr	Tyrosine aminotransferase	Q9LVY1	58220/1
47	Tyr	Phenylalanine 4-hydroxylase	P00439	37855/1
48	Phe/Trp	Class-II DAHP synthetase-like protein	Q9SK84	
49	Phe/Trp	Phospho-2-dehydro-3-deoxyheptonate aldolase, tyrosine-inhibited	P32449	
50	Phe/Trp	Pentafunctional AROM polypeptide	P08566	
51	Phe/Trp	Chorismate mutase	P32178	
52	Phe/Trp	Chorismate synthase	P28777	
53	Phe/Trp	Anthranilate synthase component 1	P00899	
54	Phe/Trp	Anthranilate phosphoribosyltransferase	P07285	
55	Trp	Tryptophan synthase	Q42529	
56	His	ATP phosphoribosyltransferase	P00498	
57	His	Imidazole glycerol phosphate synthase hisHF	P33734	
58	His	Histidinol-phosphate aminotransferase	P07172	
59	His	Histidine biosynthesis trifunctional protein	P00815	
60	His	Histidinol dehydrogenase	Q9C5U8	
61	Val/Leu/Ile	Acetolactate synthase	P07342	8385/1
62	Val/Leu/Ile	Ketol-acid reductoisomerase, mitochondrial	P06168	
63	Val/Leu/Ile	Dihydroxy-acid dehydratase, mitochondrial	P39522	127954/1
64	Val/Leu/Ile	Branched-chain-amino-acid aminotransferase, cytosolic	P47176	85088/1
65	Leu	2-isopropylmalate synthase	P06208	
66	Leu	3-isopropylmalate dehydratase	P07264	
67	Leu	3-isopropylmalate dehydrogenase	P04173	
68	Ile	Threonine dehydratase, mitochondrial	Q9ZSS6	57366/1
69	Lys	Homocitrate synthase, mitochondrial	Q12122	
70	Lys	Kynurenine/α-aminoadipate aminotransferase,mitochondrial	Q8N5Z0	
71	Lys	Homoaconitase, mitochondrial	P49367	
72	Lys	Homoisocitrate dehydrogenase, mitochondrial	P40495	
73	Lys	L-aminoadipate-semialdehyde dehydrogenase	P07702	127184/1
74	Lys	Saccharopine dehydrogenase [NADP(+), L-glutamate-forming]	P38999	1580/1
75	Lys	Saccharopine dehydrogenase [NAD(+), L-lysine-forming]	P38998	76032/1
76	Lys	4-hydroxy-tetrahydrodipicolinate synthase 2, chloroplastic	Q9FVC8	
77	Lys	Dihydrodipicolinate synthase	Q0WSN6	
78	Lys	Diaminopimelate decarboxylase 1	Q949X7	37096/1? [f]
79	Thr	Threonine synthase	P16120	10016/1

[a] The UniProt Accession Number shown is for the seed sequence used to identify the *Aiptasia* transcript. For an *Aiptasia* transcript to be listed, its best reciprocal BLAST hit (to the same species as the seed sequence) had to be the seed sequence itself or to a sequence encoding a paralogous protein. Where no transcript is listed, no *Aiptasia* homologue of the seed sequence could be identified with confidence.

[b] Downregulated (transcript 3911/1) and upregulated (95925/1) in symbiotic relative to aposymbiotic anemones (see Figure 4). Both proteins had a *Bacterioides thetaiotaomicron* NAD(P)-utilizing glutamate dehydrogenase (UniProt P94598) as their top BLAST hit.

[c] No significant differential expression in symbiotic relative to aposymbiotic anemones.

[d] In contrast to transcript 50131/1 (MS in Figure 5), transcript 55393/1 showed no differential expression in symbiotic *vs.* aposymbiotic anemones.

[e] *S*-adenosyl-methionine (see Figure 5).

[f] Although this *Aiptasia* transcript met the formal criterion for inclusion (footnote a), the number of genomic reads mapping to it barely exceeded our cut-off for calling a sequence cnidarian (see Table 2), so that it may represent a contaminant rather than an *Aiptasia* gene encoding a homologue of this typically bacterial and plant enzyme.
