# Supplementary material for: Extensive Differences in Gene Expression Between Symbiotic and Aposymbiotic Cnidarians
Source: G3 (Bethesda). 2013 Dec 24;4(2):277–95. doi: 10.1534/g3.113.009084 (PMC3931562; doi:10.1534/g3.113.009084)
Supplement: Supporting Information [file supp_g3.113.009084_TableS6.pdf]

Table S6 Genes potentially involved in host tolerance of the symbiont that are differentially expressed between symbiotic and aposymbiotic anemones. [a]

Protein (from top BLAST hit)	UniProt accession number	Locus #/ transcript #	BLAST-hit E-value	Fold-Change [b]
A. Response to oxidative stress				
Catalase	P04040	100968/1	0	-4.7
ADAM (disintegrin and metalloproteinase domain-containing protein) 9	Q13443	123296/1	2e-62	-3
Transient receptor potential cation channel (subfamily M, member 2)	Q91YD4	125627/1	7e-16	-2.9
Peroxidasin-related protein 1	Q92626	99631/1	4e-06	-2.5
Dual oxidase 2	Q8HZK2	17080/2	5e-27	-2
Allene oxide synthase-lipoxygenase	O16025	9291/1	4e-38	-1.8
Soluble guanylate cyclase 88E	Q8INF0	7254/1	1e-171	1.9
Peroxidasin-related protein 2	A1KZ92	57146/1	7e-25	2.9
B. Inflammation/tissue remodeling/response to wounding				
Transmembrane serine protease 6 [c]	Q9DBI0	81296/1	3e-50	-6.1
Plasma kallikrein [d]	P14272	12789/1	7e-47	-4
Mannan-binding lectin serine peptidase 1 (MASP-1) [e]	Q8CHN8	36375/1	5e-12	-3
Plasminogen [f]	P00747	21286/1	3e-47	-3
Plasma kallikrein [d]	P03952	84752/1	1e-48	-2.9
Ephrin type-a receptor 3 [g]	P29320	6695/1	2e-63	-2.8
Phospholipase A2 (isoform 4) [h]	Q6T179	3740/5	1e-26	-2.4
Arachidonate 5-lipoxygenase [i]	P48999	55879/1	5e-28	-1.7
Plasma kallikrein [d]	P26262	73922/1	8e-40	2.1
Ficolin 2 [j]	Q15485	62279/1	1e-42	2.2
Vanin-I [k]	Q58CQ9	48344/1	8e-118	2.6
Discoidin, CUB, and LCCL domain containing 2 [l]	Q91ZV2	80843/1	3e-12	2.7
Hepatocyte nuclear factor 4 (alpha) [m]	P22449	34830/4	1e-113	3.6
Adenosine A2b receptor [n]	O13076	66307/1	2e-17	4.2
Scavenger receptor class B member 1 [o]	Q8WTV0	77179/1	9e-65	28
C. Apoptosis/cell death				
Transcription factor E2F2	P56931	1568/1	5e-07	-4.2
Receptor-binding cancer antigen expressed on SiSo cells	Q865S0	42283/1	4e-06	-2.7
Tumor protein p73	Q9JJP2	88973/1	2e-35	-2.4
Paired box protein Pax-3	P23760	46973/1	3e-43	-1.8
Apoptosis-inducing factor 1 (mitochondrial)	Q9JM53	21845/2	1e-171	-1.8
TNF (Tumor Necrosis Factor) receptor associated factor 3	Q13114	30586/1	2e-74	1.8
TNF superfamily member 12	O43508	18277/1	1e-07	1.9

Kruppel-like factor 11	O14901	58173/1	8e-51	2.6
G1 to S phase transition 1	P15170	25564/1	0	2.8
Growth arrest and DNA damage-inducible protein (GADD45 gamma)	Q9Z111	55453/1	6e-09	5.1
Ribonucleoside-diphosphate reductase (small chain C)	Q9LSD0	18748/1	1e-132	12
Organic cation transporter	Q9VCA2	88336/1	6e-35	44
TNF receptor superfamily member 27	Q8BX35	94982/1	9e-10	60

[a] The set of all transcripts displaying differential expression by RNA-Seq was analyzed to identify biological processes (based on GO terms) that were overrepresented in this set relative to the background transcriptome (see Materials and Methods). The sets of processes identified here (A, B, and C) emerged from this analysis and may be involved in host tolerance of the symbiont.

[b] In all but one case, the arithmetic mean of the values from the two RNA-Seq experiments is shown. For transcript 77179/1 (last line of section B), the value from Experiment 1 is shown for reasons explained in Table S3, footnote b. Positive fold-changes, expression higher in symbiotic anemones; negative fold-changes, expression higher in aposymbiotic anemones.

[c] Hydrolyzes a range of proteins including type I collagen, fibronectin, and fibrinogen and may play a role in matrix-remodeling processes (Hooper *et al.* 2003).

[d] Serine proteases activated by tissue injury or microbial invasion; they activate the release of potent pro-inflammatory cytokines that ultimately result in the release of effector molecules such as nitric oxide and tumor necrosis factor-α and can stimulate the complement innate-immunity system (Lalmanach *et al.* 2010; Moreau *et al.* 2005).

[e] Plays a role as an amplifier of the complement cascade, potentially via the activation of MASP-2 (Takahashi *et al.* 2008).

[f] The zymogen of plasmin; it can be activated via plasma kallikrein and functions in the breakdown of fibrin in fibrinolysis, the activation of proteases, and the modulation of cell adhesion (Li *et al.* 2003).

[g] A receptor tyrosine kinase that binds membrane-bound ephrin family ligands residing on adjacent cells and regulates cell-cell adhesion, cytoskeletal organization, and cell migration (Smith *et al.* 2004).

[h] Releases arachidonic acid from cellular membrane phospholipids, leading to its conversion to pro-inflammatory prostaglandins via arachidonate 5-lipoxygenase (Moreau *et al.* 2005).

[i] See note h.

[j] A lectin whose binding to microbial surface glycans can initiate activation of the complement pathway (Endo *et al.* 2007); it also appears to bind to cell-surface glycans of *Symbiodinium* (Logan *et al.* 2010).

[k] Hydrolyzes pantetheine to pantothenic acid and cysteamine, the latter of which can lead to acute and chronic epithelial inflammation (Martin *et al.* 2004).

[l] Thought to play a role in cell adhesion and wound healing (Kobuke *et al.* 2001).

[m] A transcriptional regulator that is decreased in inflammatory bowel disease and protects against chemically-induced colitis in mice (Darsigny *et al.* 2009).

[n] Its activation appears to result in inhibition of pro-inflammatory cytokine production, and mice deficient in A2b receptors are more susceptible to intestinal inflammation (Gessi *et al.* 2011).

[o] See text.
