# Supplementary material for: Extensive Differences in Gene Expression Between Symbiotic and Aposymbiotic Cnidarians
Source: G3 (Bethesda). 2013 Dec 24;4(2):277–95. doi: 10.1534/g3.113.009084 (PMC3931562; doi:10.1534/g3.113.009084)
Supplement: Supporting Information [file supp_g3.113.009084_SupportingReferences.pdf]

Supporting References

Anders, S., and W. Huber, 2010 Differential expression analysis for sequence count data. Genome Biol. 11: R106.

Darsigny, M., J.-P. Babeu, A.-A. Dupuis, E. E. Furth, E. G. Seidman *et al.*, 2009 Loss of hepatocyte-nuclear-factor-4α affects colonic ion transport and causes chronic inflammation resembling inflammatory bowel disease in mice. PLoS ONE 4: e7609.

Endo, Y., M. Matsushita, and T. Fujita, 2007 Role of ficolin in innate immunity and its molecular basis. Immunobiology 212: 371–379.

Ganot, P., A. Moya, V. Magnone, D. Allemand, P. Furla *et al.*, 2011 Adaptations to endosymbiosis in a cnidarian-dinoflagellate association: differential gene expression and specific gene duplications. PLoS Genet. 7: e1002187.

Gessi, S., S. Merighi, D. Fazzi, A. Stefanelli, K. Varani *et al.*, 2011 Adenosine receptor targeting in health and disease. Expert Opin. Investig. Drugs 20: 1591–1609.

Hooper, J. D., L. Campagnolo, G. Goodarzi, T. N. Truong, H. Stuhlmann *et al.*, 2003 Mouse matriptase-2: identification, characterization and comparative mRNA expression analysis with mouse hepsin in adult and embryonic tissues. Biochem. J. 373: 689–702.

Kenkel, C. D., M. R. Traylor, J. Wiedenmann, A. Salih, and M. V Matz, 2011 Fluorescence of coral larvae predicts their settlement response to crustose coralline algae and reflects stress. Proc. R. Soc. B 278: 2691–2697.

Ko, D. C., J. Binkley, A. Sidow, and M. P. Scott, 2003 The integrity of a cholesterol-binding pocket in Niemann-Pick C2 protein is necessary to control lysosome cholesterol levels. Proc. Natl. Acad. Sci. USA 100: 2518–2525.

Kobuke, K., Y. Furukawa, M. Sugai, K. Tanigaki, N. Ohashi *et al.*, 2001 ESDN, a novel neuropilin-like membrane protein cloned from vascular cells with the longest secretory signal sequence among eukaryotes, is up-regulated after vascular injury. J. Biol. Chem. 276: 34105–34114.

Lalmanach, G., C. Naudin, F. Lecaille, and H. Fritz, 2010 Kininogens: More than cysteine protease inhibitors and kinin precursors. Biochimie 92: 1568–1579.

Leggat, W., F. Seneca, K. Wasmund, L. Ukani, D. Yellowlees *et al.*, 2011 Differential responses of the coral host and their algal symbiont to thermal stress. PLoS ONE 6: e26687.

Lehnert, E. M., M. S. Burriesci, and J. R. Pringle, 2012 Developing the anemone *Aiptasia* as a tractable model for cnidarian-dinoflagellate symbiosis: the transcriptome of aposymbiotic *A. pallida*. BMC Genomics 13: 271.

Li, W.-Y., S. S. N. Chong, E. Y. Huang, and T.-L. Tuan, 2003 Plasminogen activator/plasmin system: A major player in wound healing? Wound Repair Regen. 11: 239–247.

Logan, D. D. K., A. C. LaFlamme, V. M. Weis, and S. K. Davy, 2010 Flow-cytometric characterization of the cell-surface glycans of symbiotic dinoflagellates (*Symbiodinium* spp.). J. Phycol. 46: 525–533.

Martin, F., M.-F. Penet, F. Malergue, H. Lepidi, A. Dessein *et al.*, 2004 Vanin-1(-/-) mice show decreased NSAID- and Schistosoma-induced intestinal inflammation associated with higher glutathione stores. J. Clin. Invest. 113: 591–597.

Moreau, M. E., N. Garbacki, G. Molinaro, N. J. Brown, F. Marceau *et al.*, 2005 The kallikrein-kinin system: current and future pharmacological targets. J. Pharmacol. Sci. 99: 6–38.

Rodriguez-Lanetty, M., W. S. Phillips, and V. M. Weis, 2006 Transcriptome analysis of a cnidarian-dinoflagellate mutualism reveals complex modulation of host gene expression. BMC Genomics 7: 23.

Smith, F. M., C. Vearing, M. Lackmann, H. Treutlein, J. Himanen *et al.*, 2004 Dissecting the EphA3/Ephrin-A5 interactions using a novel functional mutagenesis screen. J. Biol. Chem. 279: 9522–9531.

Sunagawa, S., E. C. Wilson, M. Thaler, M. L. Smith, C. Caruso *et al.*, 2009 Generation and analysis of transcriptomic resources for a model system on the rise: the sea anemone *Aiptasia pallida* and its dinoflagellate endosymbiont. BMC Genomics 10: 258.

Takahashi, M., D. Iwaki, K. Kanno, J. Xiong, M. Matsushita *et al.*, 2008 Mannose-binding lectin (MBL)-associated serine protease (MASP)-1 contributes to activation of the lectin complement pathway. J. Immunol. 180: 6132–6138.

Vandesompele, J., K. De Preter, F. Pattyn, B. Poppe, N. Van Roy *et al.*, 2002 Accurate normalization of real-time quantitative RT-PCR data by geometric averaging of multiple internal control genes. Genome Biol 3: RESEARCH0034.
