# Supplementary material for: Extensive Differences in Gene Expression Between Symbiotic and Aposymbiotic Cnidarians
Source: G3 (Bethesda). 2013 Dec 24;4(2):277–95. doi: 10.1534/g3.113.009084 (PMC3931562; doi:10.1534/g3.113.009084)
Supplement: Supporting Information [file supp_g3.113.009084_FileS1.pdf]

File S1

Supplementary Materials and Methods

Identification and Optimization of qPCR Standards for *Aiptasia*

Six housekeeping genes were selected as potential qPCR standards based on their prior use in coral studies. Gene names used here are those assigned to the *Aiptasia pallida* genes and differ in most cases from those used in the other organisms. The genes encoding 60*S* ribosomal protein L11 (*RPL11*), NADH-dehydrogenase subunit 5 (*NDH5*), and glyceraldehyde-3-phosphate-dehydrogenase (*GPD1*) were reported to be stable in *Porites astreoides* during heat stress, settlement induction, and metamorphosis (Kenkel *et al.* 2011). The genes encoding 40*S* ribosomal protein S7 (*RPS7*) and adenosylhomocysteinase (*AHC1*) were used as standards during studies of thermal stress in *Acropora aspera* (Leggat *et al.* 2011). The β-actin gene (*ACT1*) was used to explore modulation of host-gene expression (Rodriguez-Lanetty *et al.* 2006) and was used as the standard for early qPCR studies in our lab.

Primers were developed and tested for these six potential standard genes. The aposymbiotic *A. pallida* transcriptome (Lehnert *et al.* 2012) was searched using tblastx with sequences from *Porites lobata* for *NDH5*, *P. astreoides* for *RPL11*, *Urticina eques* for *GPD1*, *Acropora millepora* for *RPS7*, and *Nematostella vectensis* for *AHC1*. The loci identified in the *A. pallida* transcriptome were searched using blastx in NCBI and all top hits were indeed the genes of interest. The identified loci were then translated using ORFPredictor and the longest ORFs were used to identify conserved sequences by performing protein alignments in MacVector with sequences available from NCBI. Conserved sequences were then used to develop primers using PrimerQuest from Integrated DNA Technologies (IDT).

Primers were tested on *A. pallida* cDNA and gDNA. Primers that spanned an exon-intron junction were preferentially identified for further use (Table S7). PCR products were cloned into a TA cloning vector and electroporation-competent *E. coli* cells were transformed with the plasmids. Transformed cells were plated on Ampicillin/X-Gal plates and white/light-blue colonies were selected for colony PCR using M13 forward and reverse primers. PCR products were sequenced, and the sequences were aligned with the expected sequences from the transcriptome. All primer pairs accurately selected the sequences of interest.

Table S7 Primer sequences used for potential qPCR standards

Gene	Primer sequences
RPL11	F: AGCCAAGGTCTTGGAGCAGCTTA<br>R: TTGGGCCTCTGACAGTACAGTGAACA
RPS7	F: ACTGCAGTCCACGATGCTATCCTT<br>R: GTCTGTTGTGCTTTGTCGAGATGC
NDH5	F: AGCAGTTGGTAAGTCTGCACAA<br>R: GTAACCATGGTAGCAGCATGAA
GPD1	F: AACAGCTTTGGCAGCACCTGTAGA<br>R: TGCTTTCACAGCAACCCAGAAGAC
AHC1	F: CCATTACAGCAACAACACAGGCCA<br>R: GCATCAAACGTTGGCAGATGAAGC
ACT1	F: ACACCGTCTTGTCAGGAGGTTCAA<br>R: TCCACATCTGTTGGAAGGTGGACA

The six genes were then tested for their expression levels across 11 experimental conditions (Table S8). RNA was extracted from 3-4 medium-sized anemones from each condition using a Trizol/RNeasy hybrid protocol (details available upon request). RNA integrity was checked both by using a Nanodrop and by running samples on a 2% agarose gel. For all RNA samples used, 260/280 readings were >1.9, and two clear rRNA bands were visible. For each condition, 300 ng of RNA was reverse transcribed using the Maxima® First Strand cDNA-synthesis kit for RT-qPCR (Fermentas). 17 µL of RT product was then diluted with 23 µL of H_2O. 2 µL of this cDNA solution was then used for the qPCR reaction. Each qPCR well had 2 µL of cDNA, 2 µL of H_2O, 5 µL of *Power* SYBR® Green PCR Master Mix (Applied Biosystems), and 1 µL of a primer mix containing 1.5 µM forward (F) primer and 1.5 µM reverse (R) primer.

The primer efficiency of each primer pair was tested across a dilution series of 1:1, 1:10, 1:100, 1:1000, and 1:10000 cDNA; the calculated efficiencies were 95-105%. Possible gDNA contamination in RNA samples was tested by running RNA-only controls; these samples showed no amplification. Standard qPCR settings were used, and an additional dissociation stage was added to test for the presence of multiple products. The dissociation stage showed only one clear peak in every case.

Table S8 Experimental conditions used to test gene-expression levels by qPCR

Conditions [a]	CC7 Sym [b]	CC7 Apo [c]
Room Temperature (27ºC)	x	x
1 h heat shock (35ºC)	x	x
1.5 h heat shock (37ºC)	x	x
1 h cold shock (8ºC)	x	x
1 h incubation with 500 µg/mL dsRNA[d] (27ºC)	x	x
Kept in the dark for 1 month (27ºC)	x [e]	not done

[a] Except for the sample incubated in the dark, all anemones were incubated on a 12L:12D cycle with 25 µmol photons m^{-2} s^{-1} from Cool White fluorescent bulbs, and the manipulations indicated were performed during the light period.
[b] Symbiotic anemones (containing the endogenous population of Clade A *Symbiodinium*) of the CC7 clonal line of *Aiptasia* (Sunagawa *et al.* 2009).
[c] Aposymbiotic CC7 animals that had been cured of their endogenous *Symbiodinium* by a combination of cold shock, DCMU treatment, and extended growth in the dark (Lehnert *et al.* 2012). All anemones were screened for absence of dinoflagellates prior to use in these experiments.
[d] dsRNA (477 bp) synthesized for *A. pallida* nematogalectin gene knockdown.
[e] Represents a partially aposymbiotic condition.

Ct values for each of the six genes under each of the 11 conditions were analyzed using geNorm (Vandesompele *et al.* 2002) to determine the relative expression stabilities of the prospective standard genes; the M-values are inversely proportional to the stabilities of the genes (Table S9). *ACT1* (M = 0.625) and *AHC1* (M = 0.775) were considerably less stable in expression than the four genes shown in the table.

Statistical analysis of the qPCR results also indicates that *ACT1* should not be used as an expression standard in the study of symbiosis in *Aiptasia* due to the large expression difference between aposymbiotic and symbiotic animals: there was a significant ($p = 0.002$) up-regulation in *ACT1* expression in aposymbiotic (or mostly aposymbiotic) anemones compared to symbiotic anemones across all conditions. This was determined by normalizing qPCR Ct values with the two most stable standard genes (*RPL11* and *RPS7*) and performing a Mann-Whitney statistical test on *ACT1* expression levels in aposymbiotic and symbiotic anemones.

Table S9 Assessment of gene-expression stability under various conditions [a]

Gene	Protein encoded	geNorm M	Product Sequence	Product Length	Primer Efficiency
RPL11	Component of the 60S ribosomal subunit	0.357	AGCCAAGGTCTTGGAGCAGCTTACAGGC CAACAGCCTGTGTTTTCAAAAG **(INTRON – 236 bp)** CTCGCTACACTGTGAGATCTTT TGGAATCAGAAGGAACGAGAAGATCTCT GTTCACTGTACTGTCAGAGGCCCAA	cDNA 125 bp<br>gDNA 361 bp	98%
RPS7	Component of the 40S ribosomal subunit	0.380	ACTGCAGTCCACGATGCTATCCTTGAAGA TCTTGTCTTTCCTAGTGAAATTGTTGGCAA AAGGATAAGAGTTAAACTTGATGGTTCAC GTCTCGTTAAAGTG **(INTRON – 411 bp)** CATCTCGACAAAGCACAACAGAC	cDNA 125 bp<br>gDNA 536 bp	97%
NDH5	NADH-dehydrogenase subunit 5	0.423	AGCAGTTGGTAAGTCTGCACAATTAGGCT TACACACTTGGTTACCGGATGCAATGGAA GGT **(INTRON – 1729 bp)** CCAACTCCGG TGTCTGCCTTGATTCATGCTGCTACCATGG TTAC	cDNA 105 bp<br>gDNA 1834 bp	95%
GPD1	Glyceraldehyde-3-phosphate-dehydrogenase	0.530	AACAGCTTTGGCAGCACCTGTAGAGGCTG GGATGATATTCTGATTGGCACCTCTACCA TCACGCCATTTCT **(INTRON – 567 bp)** TCCCACTAGGTCCATCTACAGTCTTCTGGG TTGCTGTGAAAGCA	cDNA 114 bp<br>gDNA 681 bp	95%

[a] Tested across the 11 experimental conditions described in Table S8.

Accession numbers for the sequences used in developing the training and test sets for TopSort

Cnidarian dataset: *Nematostella vectensis* (AB126336.1-AB126336.1, AB450038.1-AB450044.1, AB479470.1-AB479474.1, AB495365.1-AB495368.1, AF020956.1-AF020964.1, AF085282.1-AF085283.1, AY286508.1-AY286510.1, AY339866.1-AY339873.1, AY391716.1-AY391717.1, AY465174.1-AY465182.1, AY496945.1-AY496946.1, AY496948.1-AY496949.1, AY530300.1-AY530301.1, AY687348.1-AY687350.1, AY725201.1-AY725205.1, AY730689.1-AY730697.1, DQ066724.1-DQ066725.1, DQ116032.1-DQ116034.1, DQ173687.1-DQ173698.1, DQ358699.1-DQ358704.1, DQ471325.1-DQ471326.1, DQ492688.1-DQ492689.1, DQ493899.1-DQ493901.1, DQ497246.1-DQ497247.1, DQ517920.1-DQ517928.1, DQ826414.1-DQ826417.1, DQ882654.1-DQ882656.1, EF068140.1-EF068151.1, EF173462.1-EF173463.1, EF424410.1-EF424412.1, EU092640.1-EU092641.1, EU162649.1-EU162655.1, EU394531.1-EU394532.1, EU422968.1-EU422972.1, EU877197.1-EU877198.1, FJ824849.1-FJ824851.1, GQ240844.1-GQ240851.1, GU320063.1-GU320067.1, HM004556.1-HM004558.1, HM754642.1-HM754644.1, XM_001617352.1-XM_001642094.1, U42728.2, FJ428244.1, EU289217.1, EF427936.1, DQ632751.1, DQ286294.1, DQ198160.1, AY792510.1, AY651960.1, AY534532.1, AY494080.1, AY457634.1, AY363391.1, AY226090.1, AY226076.1, AY226067.1, AY226056.1, AF540387.2, AF408421.1, AF327845.1, AB495363.1, AB274036.1, AB274034.1); *Hydra magnipapillata* (AB583744.1-AB583747.1, AM233901.1-AM233903.1, AM393878.1-AM393881.1, AY212265.1-AY212267.1, AY218839.1-AY218840.1, BK004161.1-BK004162.1, DQ073557.1-DQ073558.1, DQ127903.1-DQ127904.1, DQ449927.1-DQ449931.1, EU170504.1-EU170505.1, FJ156099.1-FJ156102.1, FJ177032.1-FJ177033.1, FJ196704.1-FJ196706.1, FJ200200.1-FJ200210.1, FJ205481.1-FJ205489.1, FJ236863.1-FJ236864.1, FJ496649.1-FJ496653.1, FJ517724.1-FJ517728.1, GQ856263.1-GQ856264.1, GU219979.1-GU219981.1, GU256274.1-GU256281.1, XM_002153740.1-XM_002153922.1, XM_002153924.1-XM_002154094.1, XM_002154096.1-XM_002154206.1, XM_002154208.1-XM_002154426.1, XM_002154428.1-XM_002154512.1, XM_002154514.1-XM_002154764.1, XM_002154766.1-XM_002154895.1, XM_002154897.1-XM_002154984.1, XM_002154986.1-XM_002155429.1, XM_002155431.1-XM_002155750.1, XM_002155752.1-XM_002156047.1, XM_002156049.1-XM_002156748.1, XM_002156750.1-XM_002157387.1, XM_002157389.1-XM_002157474.1, XM_002157476.1-XM_002158411.1, XM_002158413.1-XM_002158516.1, XM_002158518.1-XM_002158837.1, XM_002158839.1-XM_002159264.1, XM_002159266.1-XM_002159291.1, XM_002159293.1-XM_002159320.1, XM_002159322.1-XM_002159398.1, XM_002159400.1-XM_002159430.1, XM_002159432.1-XM_002159454.1, XM_002159456.1-XM_002159503.1, XM_002159505.1-XM_002159563.1, XM_002159565.1-XM_002159607.1, XM_002159609.1-XM_002159628.1, XM_002159630.1-XM_002159660.1, XM_002159662.1-XM_002159732.1, XM_002159734.1-XM_002159756.1, XM_002159758.1-XM_002159789.1, XM_002159791.1-XM_002159832.1, XM_002159834.1-XM_002159873.1, XM_002159875.1-XM_002159897.1, XM_002159899.1-XM_002159921.1, XM_002159923.1-XM_002159938.1, XM_002159940.1-XM_002159972.1, XM_002159974.1-XM_002159999.1, XM_002160001.1-XM_002160050.1, XM_002160052.1-XM_002160081.1, XM_002160083.1-XM_002160109.1, XM_002160111.1-XM_002160170.1, XM_002160172.1-XM_002160207.1, XM_002160209.1-XM_002160254.1, XM_002160256.1-XM_002160282.1, XM_002160284.1-XM_002160328.1, XM_002160330.1-XM_002160388.1, XM_002160390.1-XM_002160464.1, XM_002160466.1-XM_002160488.1, XM_002160490.1-XM_002160516.1, XM_002160518.1-XM_002160520.1, XM_002160522.1-XM_002160546.1, XM_002160548.1-XM_002160549.1, XM_002160551.1-XM_002160590.1, XM_002160592.1-XM_002160609.1, XM_002160611.1-XM_002160643.1, XM_002160645.1-XM_002160677.1, XM_002160679.1-XM_002160736.1, XM_002160738.1-XM_002160762.1, XM_002160764.1-XM_002160793.1, XM_002160795.1-XM_002160812.1, XM_002160814.1-XM_002160825.1, XM_002160827.1-XM_002160916.1, XM_002160918.1-XM_002160934.1, XM_002160936.1-XM_002160939.1, XM_002160941.1-XM_002160987.1, XM_002160989.1-XM_002161016.1, XM_002161018.1-XM_002161068.1, XM_002161070.1-XM_002161111.1, XM_002161113.1-XM_002161217.1, XM_002161219.1-XM_002161238.1, XM_002161240.1-XM_002161303.1, XM_002161305.1-XM_002161357.1, XM_002161359.1-XM_002161405.1, XM_002161407.1-XM_002161440.1, XM_002161442.1-XM_002161495.1, XM_002161497.1-XM_002161541.1, XM_002161543.1-XM_002161571.1, XM_002161573.1-XM_002161614.1, XM_002161616.1-XM_002161633.1, XM_002161635.1-XM_002161742.1, XM_002161744.1-XM_002162017.1, XM_002162019.1-XM_002163255.1, XM_002163257.1-XM_002163587.1, XM_002163589.1-XM_002164597.1, XM_002164599.1-XM_002165037.1, XM_002165039.1-XM_002165179.1, XM_002165181.1-XM_002165206.1, XM_002165208.1-XM_002165386.1, XM_002165388.1-XM_002165426.1, XM_002165428.1-XM_002165450.1, XM_002165452.1-XM_002165555.1, XM_002165557.1-XM_002165581.1, XM_002165583.1-XM_002165658.1, XM_002165660.1-XM_002165664.1, XM_002165666.1-XM_002165741.1, XM_002165743.1-XM_002165874.1, XM_002165876.1-XM_002165937.1, XM_002165939.1-XM_002165968.1, XM_002165970.1-XM_002165993.1, XM_002165995.1-XM_002166009.1, XM_002166011.1-XM_002166023.1, XM_002166025.1-XM_002166044.1, XM_002166046.1-XM_002166072.1, XM_002166074.1-XM_002166099.1, XM_002166101.1-XM_002166227.1, XM_002166229.1-XM_002166249.1, XM_002166251.1-XM_002166268.1, XM_002166270.1-XM_002166291.1, XM_002166293.1-XM_002166400.1, XM_002166402.1-XM_002166447.1, XM_002166449.1-XM_002166477.1, XM_002166479.1-XM_002166498.1, XM_002166500.1-XM_002166520.1, XM_002166522.1-XM_002166544.1, XM_002166546.1-XM_002166566.1, XM_002166568.1-XM_002166613.1, XM_002166615.1-XM_002166665.1, XM_002166667.1-XM_002166857.1, XM_002166859.1-XM_002167064.1, XM_002167066.1-XM_002167374.1, XM_002167376.1-XM_002167520.1, XM_002167522.1-XM_002167715.1, XM_002167717.1-XM_002167771.1, XM_002167773.1-

XM_002167799.1, XM_002167801.1-XM_002167815.1, XM_002167817.1-XM_002167831.1, XM_002167833.1-XM_002167845.1, XM_002167847.1-XM_002167857.1, XM_002167859.1-XM_002167904.1, XM_002167906.1-XM_002167937.1, XM_002167939.1-XM_002168075.1, XM_002168077.1-XM_002168736.1, XM_002168738.1-XM_002168749.1, XM_002168751.1-XM_002169356.1, XM_002169358.1-XM_002169408.1, XM_002169410.1-XM_002169425.1, XM_002169427.1-XM_002169976.1, XM_002169978.1-XM_002169985.1, XM_002169987.1-XM_002169990.1, XM_002169993.1-XM_002169995.1, XM_002169997.1-XM_002170003.1, XM_002170005.1-XM_002170008.1, XM_002170010.1-XM_002170017.1, XM_002170019.1-XM_002170029.1, XM_002170031.1-XM_002170034.1, XM_002170036.1-XM_002170123.1, XM_002170125.1-XM_002170370.1, XM_002170372.1-XM_002170971.1, XM_002170973.1-XM_002171130.1, XM_002171132.1-XM_002171275.1, X70839.1, X70839.1, X67590.1, X67590.1, U53444.1, U53444.1, U36781.1, U36781.1, HQ184466.1, HQ184466.1, GU199337.1, GU199337.1, GQ983384.1, GQ983384.1, GQ856264.1, FN257513.1, FN257513.1, FJ823136.1, FJ823136.1, FJ222238.1, FJ222238.1, FJ154842.1, FJ154842.1, EU877199.1, EU877199.1, EU787490.1, EU787490.1, EU442372.1, EU442372.1, EU178740.1, EU015880.1, EU015880.1, EF370474.1, EF370474.1, EF010985.1, EF010985.1, DQ518873.1, DQ518873.1, DQ073560.1, DQ073560.1, DQ072591.1, DQ072591.1, AY841903.1, AY841903.1, AY458134.1, AY458134.1, AY422083.1, AY422083.1, AY372112.1, AY372112.1, AY332609.1, AY332609.1, AY225467.1, AY225467.1, AY216501.1, AY216501.1, AY213094.1, AY213094.1, AM233513.2, AM182483.1, AF307098.1, AF307098.1, AF188478.1, AF188478.1, AF043907.1, AF043907.1)

Fungal dataset: *Schizosaccharomyces pombe* (gi|301736437-301750575|); *Aspergillus niger* (AJ239738.1-AJ239987.1, BE758760.1-BE760957.1, CK769166.1-CK769173.1, DR697868.1-DR710686.1, EY187740.1-EY188372.1, EY223258.1-EY254202.1); *Neurospora crassa* (AA574464.1-AA574465.1, AA601776.1-AA601777.1, AA738494.1-AA738501.1, AA774383.1-AA774387.1, AA897792.1-AA899039.1, AA901496.1-AA902101.1, AA908001.1-AA908006.1, AI318697.1-AI320510.1, AI320569.1-AI322045.1, AI328149.1-AI330327.1, AI391954.1-AI391955.1, AI391957.1-AI392604.1, AI397485.1-AI399633.1, AI416404.1-AI416428.1, AW708018.1-AW719192.1, AW721859.1-AW725138.1, BE900092.1-BE900100.1, BF072409.1-BF072839.1, BF739420.1-BF739760.1, BG278041.1-BG280722.1, FK707478.1-FK707538.1, GE917356.1-GE999999.1, GH000001.1-GH158787.1); *Saccharomyces cerevisiae* (AA417440.1-AA417500.1, AA417502.1-AA417537.1, DB636784.1-DB668630.1, EG999314.1-T17502.1, T17635.1-T36312.1, T39110.1-X78018.1, EH038222.1)

Dinoflagellate dataset: *Alexandrium tamarense* (CF751845.1-CF751962.1, CF774560.1-CF774855.1, CF947047.1-CF948546.1, CK431405.1-CK433904.1, CK782344.1-CK786698.1, CV553867.1-CV555405.1), *Alexandrium catenella* (EX454357.1-EX464203.1, AB212072.1), *Alexandrium ostenteldii* (HO658038.1-HO663459.1,HO652585.1-HO658036.1), *Alexandrium mitum* (GW792032.1-GW792241.1, GW792243.1-GW792256.1, GW792258.1-GW792278.1, GW792280.1-GW792403.1, GW792405.1-GW792489.1, GW792491.1-GW792620.1, GW792634.1-GW792636.1, GW792645.1-GW792648.1, GW792652.1-GW792654.1, GW792655.1-GW792657.1, GW792662.1-GW792666.1, GW792680.1-GW792682.1, GW792706.1-GW792708.1, GW792710.1-GW792769.1, GW792771.1-GW792774.1, GW792776.1-GW792787.1, GW792789.1-GW792804.1, GW792805.1-GW792807.1, GW792820.1-GW792821.1, GW792823.1-GW792861.1, GW792863.1-GW792865.1, GW792871.1-GW792976.1, GW792980.1-GW792985.1, GW792988.1-GW793010.1, GW793012.1-GW793017.1, GW793019.1-GW793113.1, GW793115.1-GW793179.1, GW793182.1-GW793185.1, GW793187.1-GW793190.1, GW793193.1-GW793227.1, GW793229.1-GW793255.1, GW793257.1-GW793268.1, GW793270.1-GW793275.1, GW793277.1-GW793281.1, GW793283.1-GW793359.1, GW793361.1-GW793364.1, GW793366.1-GW793367.1, GW793369.1-GW793376.1, GW793411.1-GW793413.1, GW793552.1-GW793554.1, GW793755.1-GW793757.1, GW793766.1-GW793768.1, GW793832.1-GW793834.1, GW793846.1-GW793851.1, GW793853.1-GW793894.1, GW793896.1-GW793925.1, GW793927.1-GW793942.1, GW793944.1-GW793946.1, GW793952.1-GW793954.1, GW793962.1-GW793964.1, GW794043.1-GW794045.1, GW794143.1-GW794145.1, GW794188.1-GW794190.1, GW794217.1-GW794219.1, GW794223.1-GW794225.1, GW794307.1-GW794309.1, GW794319.1-GW794321.1, GW794331.1-GW794334.1, GW794357.1-GW794359.1, GW794412.1-GW794414.1, GW794415.1-GW794417.1, GW794430.1-GW794441.1, GW794443.1-GW794448.1, GW794450.1-GW794460.1, GW794462.1-GW794488.1, GW794490.1-GW794530.1, GW794532.1-GW794623.1, GW794625.1-GW794643.1, GW794645.1-GW794711.1, GW794713.1-GW794894.1, GW794896.1-GW794988.1, GW794990.1-GW795077.1, GW795079.1-GW795089.1, GW795091.1-GW795182.1, GW795184.1-GW795186.1, GW795188.1-GW795246.1, GW795248.1-GW795278.1, GW795280.1-GW795375.1, GW795377.1-GW795395.1, GW795398.1-GW795406.1, GW795410.1-GW795412.1, GW795414.1-GW795415.1, GW795417.1-GW795419.1, GW795422.1-GW795431.1, GW795434.1-GW795444.1, GW795446.1-GW795454.1, GW795458.1-GW795466.1, GW795469.1-GW795479.1, GW795500.1-GW795502.1, GW795513.1-GW795515.1, GW795520.1-GW795543.1, GW795545.1-GW795554.1, GW795557.1-GW795566.1, GW795569.1-GW795612.1, GW795614.1-GW795637.1, GW795640.1-GW795650.1, GW795652.1-GW795662.1, GW795664.1-GW795680.1, GW795682.1-GW795752.1, GW795754.1-GW795761.1, GW795763.1-GW795999.1, GW796001.1-GW796184.1, GW796186.1-GW796252.1, GW796257.1-GW796262.1, GW796264.1-GW796293.1, GW796295.1-GW796353.1, GW796355.1-GW796486.1, GW796488.1-GW796575.1, GW796608.1-GW796610.1, GW796612.1-GW796614.1, GW796616.1-GW796618.1, GW796633.1-GW796635.1, GW796656.1-GW796658.1, GW796729.1-GW796731.1, GW796752.1-GW796754.1, GW796796.1-GW796885.1, GW796573.1, GW795518.1, GW795477.1, GW794428.1, GW793940.1, GW793844.1, GW793374.1, GW792936.1, GW792940.1, GW792960.1, GW792962.1, GW792964.1, GW792883.1, GW792885.1, GW792966.1, GW792835.1, GW792839.1, GW792851.1, GW792854.1, GW792817.1, GW792794.1, GW792704.1, GW792689.1, GW792695.1, GW792676.1, GW792678.1, GW792628.1, GW792630.1, GW792618.1); *Karlodinium micrum* (EC147064.1-EC163595.1); *Karenia brevis* (CO059029.1-CO065717.1, CO517335.1-

CO517390.1, CV173737.1-CV173976.1, EX864807.1-EX878969.1, EX956452.1-EX980006.1, CV179548.1); *Symbiodinium* strain KB8 (FE537410.1-FE540062.1).

Bacterial dataset: *Escherichia coli* strain MS 175-1 (gi|EFJ63866-EFJ68735|); *Salmonella enterica* (EDZ33444-EDZ37920)
